# New world health organization guideline on anemia cut-off points: implications for children aged 6-35 months in Peru

**DOI:** 10.17843/rpmesp.2025.422.14028

**Published:** 2025-06-12

**Authors:** Miguel Campos-Sánchez, Luis Cordero-Muñoz, Enrique Velásquez-Hurtado, Nelly Baiocchi-Ureta, Marianella Miranda-Cuadros, María Inés Sánchez-Griñán, Walter Valdivia-Miranda

**Affiliations:** 1 Cayetano Heredia Peruvian University, Lima, Peru. Cayetano Heredia Peruvian University Cayetano Heredia Peruvian University Lima Peru; 2 National Institute of Health, Lima, Peru. National Institute of Health Lima Peru; 3 CERES-Nutrir, Lima, Peru. CERES-Nutrir Lima Peru; 4 Ministry of Development and Social Inclusion, Lima, Peru. Ministry of Development and Social Inclusion Lima Peru

**Keywords:** Hemoglobin, Anemia, Children, Peru

## Abstract

**Objectives.:**

To compare annual national and regional prevalence rates of anemia, using the 2001 guideline versus the new 2024 guideline in children aged 6 to 35 months residing in Peru between 2009 and 2023. To assess whether differences exist between guidelines vary by region, setting, or year.

**Materials and methods.:**

Secondary analysis of the Demographic and Family Health Survey (continuous national random sample, stratified and clustered). Hemoglobin was measured in capillary blood using Hemocue. We applied an equation (and/or table) for altitude adjustment and a cutoff point for each guideline. We calculated 95% confidence intervals [95% CI]. Differences were evaluated according to region, setting, and/or year using a generalized linear model, calculating extremes and quartiles. Estimates and models were weighted.

**Results.:**

We analyzed 120,711 children. The difference in prevalence was -6.3 [-6.6 to -6.0], p<0.001, varying by region (p<0.001), region-year (p=0.004), and region-setting (p<0.001), between -40.6 and 11.0. The percentage of children whose diagnosis differed was 11.0 [10.7 to 11.2], (p<0.001), varying between 0.0 and 40.6. The difference between the table and the equation was -3.8 [-4.0 to -3.6].

**Conclusions.:**

The prevalence differs with the new guideline (generally decreasing, but may increase), with variable differences according to region, setting, and year. The percentage with a different diagnosis also varies. These differences are of great importance for health, in some cases changing the problem from severe to moderate. The table calculation underestimates the equation calculation. Literature supports the direction of the correction, but not its magnitude.

## INTRODUCTION

Childhood anemia is a major problem ^1^^,^^2^, particularly because of its consequences for early development. In Peru, it has been and continues to be a top health priority ^2^^-^^4^. Its frequency is determined by comparing blood hemoglobin levels with cut-off points ^1^^,^^2^.

On March 6, 2024, the World Health Organization (WHO) published a new guideline ^5^^)^ on hemoglobin cut-off points for diagnosing anemia, updating the previous guideline ^6^^)^ and taking into account important recently published evidence ^7^^-^^9^. The new guideline has three main changes for young children: it lowers the cutoff point for the 6-23-month age group, modifies the altitude adjustment equation, lowering the cutoff point above 3000 meters above sea level (MASL), increasing it below that altitude, and recommends measuring venous blood with calibrated automated or portable hematology instruments, both under quality control, as the laboratory method. Peru has officially incorporated the guideline into its national guidelines ^10^.

The previous guideline ^6^ lacked solid evidence for its cutoff points, being essentially expert opinion based on a few studies. For some time now, there have been indications that the 2001 guideline and its predecessors overestimated the cutoff points in the first months of life ^11^ and in altitude adjustments ^9^, resulting in a probable overestimation of anemia prevalence.

There has been extensive international ^11^^,^^12^ and national ^3^^,^^4^ debate on this issue. Our interpretation of the consensus up to 2023 is to recognize that the evidence supported the need to modify the guidelines, as well as the direction in which adjustments to the cut-off points should be made, but did not support the magnitude of the corrections required.

The choice of normal hemoglobin values according to age and altitude could be supported by cohort studies in representative samples of large populations with complementary studies. The new guidelines instead use what is called a “statistical approach” (widely used in clinical pathology), i.e., the estimation of percentiles of the distribution in representative national surveys (and some non-representative studies) with variables (iron and inflammation markers and symptoms) that allow the selection of a “healthy” subset. This approach can fail crucially when the prevalence of some causes of anemia is not small enough to be ignored and diagnostic methods are not available to exclude them.

The cut-off points in the new 2024 guideline cite three articles ^7^^-^^9^^)^ (which, together, represent approximately just under 11% of the world population) and two other articles ^13^^,^^14^ that were not considered. There are also previous studies ^15^^-^^25^^)^ that show discrepancies with the 2001 guideline, a recent Peruvian study ^26^, and alternative proposals ^27^^-^^30^. We will review this evidence in more detail in the discussion section.

The implementation of the new WHO 2024 guideline will change prevalence figures. The magnitude of these changes, their effect on temporal trends, their homogeneity within the country, and their effects on decision-making are not yet clear. As a result of not knowing these magnitudes, our study aimed to (a) compare the annual national prevalence of anemia when using the WHO 2001 guideline versus the WHO 2024 guideline for the population of children aged 6 to 35 months residing in Peru between 2009 and 2023; (b) compare cumulative regional prevalence rates; and (c) assess whether the magnitude of the differences varies by region, setting (urban or rural), or calendar year, alone or in combination.

**KEY MESSAGES**

Motivation for the study. In 2024, the World Health Organization modified the cut-off points that define anemia. The magnitude of the change in the prevalence of anemia in children aged 6-35 months in Peru, compared to the 2001 guideline, is unknown.

Main findings. Between 2009 and 2023, we found significant and heterogeneous differences (a) nationally, (b) between and within regions, and (c) depending on the calculation technique (table or equation).

Implications. The rationale for the 2024 guideline, while much better than that for the 2001 guideline, is not sufficient. The new guideline should be adopted with caution, both in individual care and population-related decisions.

## MATERIALS AND METHODS

This is a diagnostic study that compared the prevalence of anemia under two guidelines, WHO 2001 and 2024, through a secondary analysis of data from the Demographic and Family Health Survey (ENDES) ^31^ 2009 to 2023, conducted by the National Institute of Statistics and Informatics (INEI), every five years since 1986 and annually since 2004.

### Population and sample

The ENDES ^31^^,^^32^ has a repeated cross-sectional design based on a national random sample. The framework, maintained by the INEI, groups the entire country into clusters of approximately 120-140 households, distributed in strata by region (the country’s first level of administrative subdivision) and socioeconomic level. The INEI calculates the sample size as a multipurpose survey, resulting in approximately 35,000 households each year. In the first stage, clusters are randomly selected from each stratum with probability proportional to size and distributed across the calendar months. During the second stage, field teams update the list of households in each cluster and randomly select the interviewees. Age (date of birth) and sex are obtained through self-reporting. Rural areas are defined as populated centers with fewer than 2,000 inhabitants. Altitude (MASL, meters above sea level) is defined by the INEI for each cluster (since 2016, the INEI has collected household altitude using GPS, but this data is not used here). In subsamples of various population subgroups, hemoglobin in capillary blood is measured using portable hemoglobinometers (staff are standardized at least once a year). The INEI continuously updates the framework by readjusting the definition and sampling techniques. The anonymized individual data and documentation are publicly available.

Individual data were analyzed and consolidated, adjusting the weighting for each year according to the national population projection ^33^ and redefining strata such as the 25 regions × 2 settings (urban and rural). We included children aged 6-35 months with complete hemoglobin, who had spent the previous night at home, for a total of 120,711 children.

### Variables

The following variables were obtained from the ENDES household questionnaire for each child, RECH0 file (and, where indicated, from the children’s modules in the individual questionnaire, files REC21 and RECH6): Age in months (RECH6.HC1), altitude of the cluster in meters above sea level (HV040), urban or rural area (HV025), administrative region (HV023, with the province of Callao separated and the province of Lima included in the Lima region), year of the interview (HV007), hemoglobin g/dl (Hb) in capillary blood measured with a Hemocue Hb201 portable device (RECH6.HC53), stratum (HV022), cluster (HV001), dwelling and household (HHID), child (RECH6.HC1), and expansion factor (HV005 except HV005X for 2015 and HV005A for 2020). The main analysis file is RECH6, to which the REC21 and RECH0 files were linked using CASEID and/or HHID.

### Analysis

The dichotomous condition of anemia was calculated for each child using both guidelines ^5^^,^^6^ by subtracting an altitude adjustment from the measured Hb before comparing it with a cutoff point. For the 2001 guideline, the adjustment was the Centers for Disease Control (CDC) equation ^23^^,^^34^ (from 1000 MASL starting in 2022) and the cutoff was 11 g/dL. For the 2024 guideline, the adjustment was the equation specified in the guideline ^8^^,^^9^ from 1 MASL, and the cutoff was 10.5 (age <24 months) or 11 (age 24-35 months) g/dL. An altitude adjustment variant was also calculated using the table, not the equation, specified in the 2024 guideline.

The estimates were adjusted to the sample design ^35^ with their respective 95% confidence intervals (95% CI). To illustrate the implications, adjusted annual prevalences were also estimated by combining three groupings: age (6-11, 12-23, and 24-35 months), altitude (0-999, 1000-2999, and 3000+ MASL), and setting (urban, rural).

The difference in diagnoses for each individual was calculated as the difference between their anemia diagnoses (coded 0 for no anemia and 1 for anemia) according to the 2024 guideline minus the 2001 guideline. The average difference in prevalence was estimated as the average of the individual diagnostic differences, with their respective 95% CI. By definition, these average differences are not equal to zero for age and altitude, and therefore we did not statistically compare these variables. Instead, we evaluated the average differences by region, setting, and/or year (which are a consequence of their age and altitude distributions). Therefore, we applied a GLM (generalized linear model, with normal distribution and adjusted to the sample design), with the unit of analysis being the surveyed child, the dependent variable being the individual difference, and the independent variables being the three categorical variables, in a saturated model (with all 2- and 3-term interactions). To assess variability, we calculated the extremes and quartiles of the distribution of estimated differences in the combinations of region, setting, and year. When prevalence is greater than 40%, the official WHO classification ^1^ changes from moderate to severe public health problem and recommends universal daily supplementation in the 6-23-month age range.

We also estimated the national percentage of children whose diagnosis differs under the two guidelines and the difference in prevalence when calculated using the WHO 2024 guideline in two ways (equation versus table).

The R program ^36^ version 4.4.2 was used with the tidyverse and survey packages and their dependencies. The program and consolidated pre-publication data are available at https://github.com/vipermcs/pdata/blob/main/CUTHBN32024.zip. A supplement with the ENDES sources, GLM models, and R program is attached.

### Ethical considerations

The study used only publicly available anonymized individual data, so we did not request review by an ethics committee.

## RESULTS

**Figure 1:**
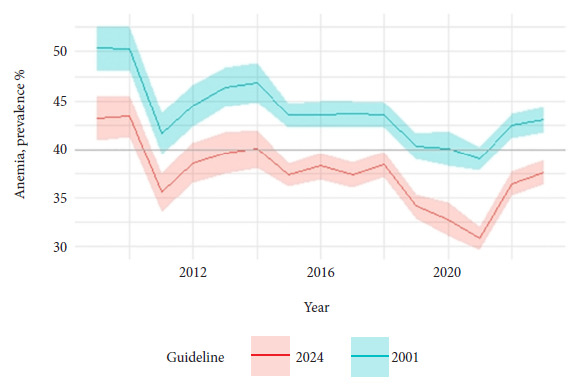
Annual prevalence of anemia in children aged 6-35 months, Peru, 2009-2023.

Figure 1 shows the trend in annual national prevalence under the two guidelines. This trend is similar under both guidelines (within the confidence margins) and that under the new guideline, prevalence is no longer considered a serious public health problem but rather a moderate one. Table 1 also shows the annual national prevalence figures from Figure 1 and adds estimates of the difference in prevalence. The magnitude of the national difference is similar over the years. Each year is different from 0 (p<0.001) with no differences between years (p=0.067).

The horizontal axis represents the calendar year. The vertical axis represents the prevalence of anemia. The lines connect the weighted annual point estimates. The bands connect the 95% confidence limits. The gray horizontal line separates severe and moderate public health problems, according to the WHO definition. The color corresponds to each WHO guideline; green is the previous guideline from 2001 and red is the new guideline for 2024.

**Table 1:** Annual percentage prevalence of anemia in children aged 6-35 months, Peru, 2009-2023.

Year	2001 guideline prev.	2024 guideline prev.	Diff. 2024 - 2001 Prev.
2009	50.4 (48.1 to 52.6)	43.2 (41.0 to 45.4)	-7.2 (-8.6 to -5.7)
2010	50.3 (48.1 to 52.5)	43.4 (41.2 to 45.6)	-6.9 (-8.2 to -5.6)
2011	41.6 (39.5 to 43.7)	35.6 (33.6 to 37.6)	-6.0 (-7.1 to -4.9)
2012	44.5 (42.3 to 46.6)	38.6 (36.5 to 40.7)	-5.9 (-7.1 to -4.6)
2013	46.4 (44.3 to 48.4)	39.7 (37.5 to 41.8)	-6.7 (-8.2 to -5.3)
2014	46.8 (44.8 to 48.9)	40.1 (38.1 to 42.1)	-6.8 (-8.1 to -5.4)
2015	43.5 (42.3 to 44.8)	37.4 (36.2 to 38.6)	-6.1 (-6.9 to -5.3)
2016	43.6 (42.2 to 45.0)	38.3 (36.9 to 39.6)	-5.3 (-6.1 to -4.5)
2017	43.6 (42.2 to 45.0)	37.4 (36.1 to 38.7)	-6.2 (-7.1 to -5.3)
2018	43.5 (42.3 to 44.8)	38.5 (37.2 to 39.7)	-5.1 (-5.9 to -4.3)
2019	40.3 (39.0 to 41.6)	34.2 (32.9 to 35.4)	-6.1 (-7.0 to -5.3)
2020	40.1 (38.3 to 41.9)	32.8 (31.1 to 34.5)	-7.3 (-8.4 to -6.2)
2021	39.0 (37.8 to 40.3)	30.9 (29.7 to 32.1)	-8.1 (-8.9 to -7.3)
2022	42.4 (41.2 to 43.7)	36.5 (35.3 to 37.7)	-5.9 (-6.8 to -5.1)
2023	43.1 (41.8 to 44.4)	37.7 (36.4 to 38.9)	-5.4 (-6.3 to -4.6)
2009-2023			-6.3 (-6.6 to -6.0)

Format: estimate (lower and upper limits of 95% confidence interval). Prev: prevalence. Diff: difference between prevalences in 2024 minus 2001.

Table 2 shows the regional prevalence rates, accumulated for the entire period 2009-2023, under the two guidelines. The variation in the magnitude of the differences is evident. Each of the regions, except Cajamarca and Moquegua, is different from 0 (p<0.001) with differences between regions (p<0.001). Table 2 also shows the percentages of children whose diagnosis changed for each region, also accumulated during 2009-2023. Each region is greater than 0 (p<0.001) with differences between regions (p<0.001).

**Table 2:** Regional percentage prevalence of anemia in children aged 6-35 months, Peru, 2009-2023.

Region	2001 guideline prev.	2024 guideline prev.	Diff. 2024 - 2001 Prev.	Perc. change in Dx. (anemia→normal or normal→anemia) between 2001 and 2024
Amazonas	46.1 (44.3 to 47.9)	48.9 (47.1 to 50.7)	+2.8 (+1.8 to +3.8)	8.8 (7.9 to 9.8)
Ancash	44.0 (42.2 to 45.9)	36.3 (34.6 to 38.1)	-7.7 (-8.8 to -6.6)	10.6 (9.5 to 11.7)
Apurimac	53.9 (51.8 to 55.9)	43.2 (41.2 to 45.2)	-10.7 (-12.0 to -9.3)	14.1 (12.8 to 15.3)
Arequipa	41.2 (39.2 to 43.3)	42.5 (40.4 to 44.6)	+1.3 (+0.1 to +2.4)	8.4 (7.5 to 9.4)
Ayacucho	50.4 (48.5 to 52.2)	44.2 (42.3 to 46.0)	-6.2 (-7.5 to -4.9)	11.7 (10.5 to 12.9)
Cajamarca	40.2 (38.2 to 42.2)	40.3 (38.4 to 42.3)	+0.1 (-1.0 to +1.3)	8.4 (7.3 to 9.4)
Callao	39.3 (36.8 to 41.9)	31.7 (29.4 to 34.0)	-7.6 (-9.0 to -6.2)	9.4 (7.8 to 11.0)
Cusco	56.6 (54.5 to 58.7)	43.6 (41.5 to 45.7)	-13.1 (-14.6 to -11.5)	15.0 (13.5 to 16.4)
Huancavelica	58.8 (56.7 to 60.9)	41.1 (38.8 to 43.3)	-17.7 (-19.4 to -16.0)	18.6 (17.0 to 20.3)
Huanuco	48.0 (46.1 to 49.9)	43.1 (41.2 to 44.9)	-5.0 (-6.5 to -3.5)	12.5 (11.3 to 13.7)
Ica	40.7 (39.0 to 42.4)	38.3 (36.6 to 40.1)	-2.3 (-3.3 to -1.4)	9.0 (8.2 to 9.9)
Junin	52.4 (50.5 to 54.3)	44.6 (42.6 to 46.5)	-7.8 (-9.1 to -6.5)	12.5 (11.4 to 13.7)
La Libertad	39.6 (37.7 to 41.5)	31.8 (30.0 to 33.6)	-7.8 (-9.0 to -6.6)	10.3 (9.2 to 11.4)
Lambayeque	37.4 (35.8 to 39.0)	31.0 (29.4 to 32.7)	-6.4 (-7.4 to -5.4)	10.1 (9.2 to 11.1)
Lima	34.8 (33.7 to 35.9)	29.5 (28.4 to 30.6)	-5.3 (-5.9 to -4.6)	9.2 (8.5 to 9.8)
Loreto	55.9 (54.4 to 57.5)	48.5 (46.9 to 50.1)	-7.4 (-8.3 to -6.5)	10.8 (9.9 to 11.7)
Madre de Dios	58.3 (56.6 to 60.0)	55.5 (53.8 to 57.1)	-2.8 (-3.8 to -1.8)	9.9 (8.9 to 10.9)
Moquegua	38.4 (36.2 to 40.5)	38.4 (36.3 to 40.5)	+0.0 (-1.2 to +1.3)	9.3 (8.2 to 10.3)
Pasco	56.5 (54.6 to 58.3)	43.6 (41.6 to 45.6)	-12.9 (-14.7 to -11.0)	18.8 (17.4 to 20.3)
Piura	41.1 (39.6 to 42.7)	35.6 (34.1 to 37.1)	-5.5 (-6.5 to -4.5)	10.2 (9.3 to 11.1)
Puno	73.8 (72.1 to 75.6)	47.2 (45.1 to 49.4)	-26.6 (-28.4 to -24.8)	27.2 (25.4 to 28.9)
San Martin	43.3 (41.6 to 45.0)	44.0 (42.2 to 45.8)	+0.7 (-0.2 to +1.7)	8.8 (8.0 to 9.7)
Tacna	38.0 (36.1 to 40.0)	37.5 (35.6 to 39.4)	-0.5 (-1.7 to +0.7)	9.0 (7.9 to 10.1)
Tumbes	47.2 (45.5 to 48.8)	40.0 (38.2 to 41.7)	-7.2 (-8.3 to -6.0)	10.6 (9.5 to 11.6)
Ucayali	58.3 (56.7 to 59.8)	52.2 (50.6 to 53.9)	-6.1 (-7.0 to -5.1)	10.7 (9.9 to 11.6)

Format: estimate (lower to upper). Prev: prevalence. Diff: difference between prevalences. 2024 guideline minus 2001 guideline. Perc: percentage. Dx: diagnosis.

The change in diagnosis (fifth column) includes those who go from anemic to non-anemic as well as those who go from non-anemic to anemic.

**Figure 2:**
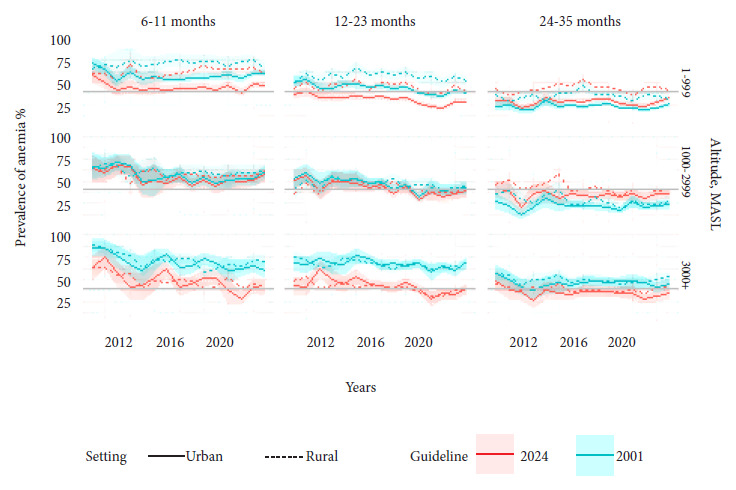
Annual prevalence of anemia in subdomains in children aged 6-35 months, Peru, 2009-2023.

Figure 2 shows the trend in prevalence over time with both guidelines for each combination of setting, age group, and altitude level. It shows that there are differences in the estimated prevalence of anemia between the guidelines due to the modification of the adjustment equation according to altitude in children aged 24 months or older, for whom the cutoff point for both guidelines is the same. These differences are not linear and even show an increase in the prevalence of anemia when the altitude is below 3000 m above sea level. In children under 24 months of age, the difference in the prevalence of anemia between the guidelines results both from the change in the adjustment equation according to altitude and from the reduction in the cutoff point at sea level in this age group.

Each panel corresponds to a combination of age group (top axis) and altitude group (right axis). The bottom axis is the calendar year. The left axis is the prevalence of anemia. The lines connect the weighted annual point estimates. The bands connect the 95% confidence limits. The line type corresponds to the setting, urban (solid line) or rural (dashed line). The gray horizontal lines separate severe and moderate public health problems, according to the WHO definition. The color corresponds to each WHO standard, green is the previous 2001 guideline and red is the new 2024 guideline.

The difference in national prevalence (2024 guideline minus 2001 guideline) for the entire period is -6.3% (95% CI -6.6 to -6.0, p<0.001). This difference varies significantly (Table 3) by year, region, region-year, and region-area, a variability reflected in its distribution across 735 combinations of region, setting, and year, ranging from -40.6 to 11.0, with quartiles at -9.9, -5.8, and -1.1, covering a wide range of differences with different practical implications.

**Table 3:** Modeling summary.

Outcome variable	1^st^ Model	2^nd^ Model
	Difference	Change
Number of individual observations	120711	120711
Deviance. null	12777	83576
Degrees of freedom. null	120710	120710
Akaike information criterion. AIC	68471	82742
Bayesian information criterion. BIC	20889	89665
Deviance	12266	81042
Degrees of freedom. residue	33386	33386
Squared pseudo-R. McFadden	0.040	0.030
Year. p	0.014	0.005
Region. p	<0.001	<0.001
Setting. p	0.708	<0.001
Year:Region. p	0.006	0.084
Year:Setting. p	0.251	0.405
Region:Setting. p	<0.001	<0.001
Year:Region:Setting. p	0.234	0.047

Generalized linear models. Difference: difference between individual diagnoses according to the 2001 guideline versus the 2024 guideline. Change: individual changes diagnosis according to the 2001 and 2024 guidelines. p: p-values for zero coefficient hypothesis. The symbol “:” denotes interaction.

The national percentage for the entire period of children whose diagnosis changed under the two guidelines is 11.0 (95% CI: 10.7 to 11.2, p<0.001). This percentage varies significantly (Table 3) according to year, region, setting, region-setting, and year-region-setting, a variability reflected in its distribution across 735 combinations of region, setting, and year, ranging from 0 to 40.6, with quartiles at 8.3, 10.7, and 14.1, also covering a wide range of changes.

**Figure 3:**
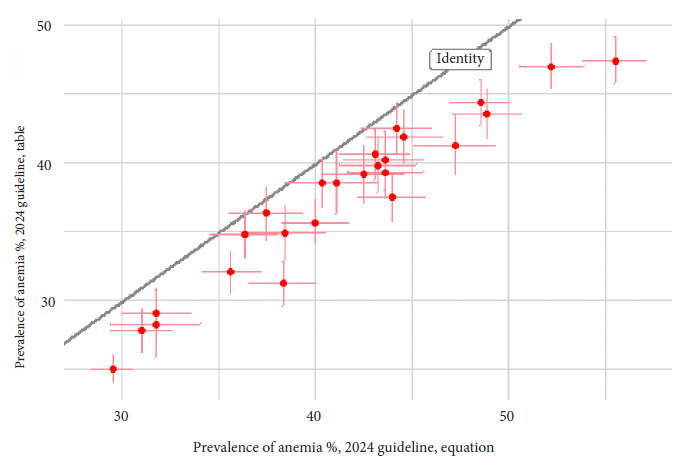
Regional prevalence of anemia according to type of altitude adjustment in children aged 6-35 months, Peru, 2009-2023.

The 2024 guideline describes two ways to obtain altitude adjustment: a classification table and an equation. The table does not define adjustment for altitudes of 5000 MASL or higher (the 2001 guideline presented both methods and did not apply any adjustment below 1000 MASL). When plotting adjustment versus altitude, the equation results in a continuous curve, on the other hand, for the table it is a stepped line. The national difference between the two methods is -3.8 (95% CI from -4.0 to -3.6). Figure 3 compares the regional prevalences (cumulative for the entire 2009-2023 period) of both methods. We found that the prevalence obtained with the 2024 guideline using both methods differs significantly. Using the table produces varying degrees of prevalence underestimation.

Each red dot is a weighted estimate of regional anemia prevalence. The horizontal axis is the estimate using the 2024 guideline equation. The vertical axis is the estimate using the 2024 guideline table. The pink vertical and horizontal lines are the 95% confidence intervals. The gray diagonal line is the expected value if both calculation techniques were equivalent.

## DISCUSSION

Our results show important and statistically significant differences with the new guideline, regarding prevalence (mostly reduction, but also increase) to varying degrees according to region, setting and year, with similar but not identical temporal trends. This results from the non-linearity of the norms, the internal distribution of the population by altitude and the closeness of the population positions to the cut-off points. It is logical that the new norm differs; what our analysis provides is an appreciation of its magnitude and variability in a real and diverse population.

In the following paragraphs, we review the articles that support the new guideline. One article ^7^^)^ is about ages from 6 months to 65 years. Its relevant data sources (1999-2019) are two surveys (US and Ecuador) and one cohort (Canada/TARGet Kids!). This analysis adjusts a curve in the second half of life, which better reflects physiology than the abrupt transition in the guidelines, which generates an artificial jump at 24 months.

Two articles are on children who live in altitude <60 months and women aged 15-49 ^9^ as well as school-age children ^8^. Their relevant data sources (1999-2019, BRINDA project) are surveys (Mexico, Colombia, Ecuador, Afghanistan, Nepal, Laos, Malawi, Papua New Guinea, Azerbaijan, Georgia, United Kingdom, and United States), one surveillance study (Guatemala/SIVESNU), and two assessments (Ghana/GIFTS and Bolivia/NIDI El Alto). In addition, the 2024 guideline describes an expansion of the analysis, which would add 5 years of data from the Peru/SIEN surveillance (the only source above 4000 MASL). In children <60 months, they report ^9^ some heterogeneity between countries. The equation from the 2024 guideline is almost identical to that proposed for schoolchildren ^8^.

There are two articles mentioned in the 2024 guideline but not considered due to different inclusion criteria. A review by BRINDA ^13^ is early evidence of the cut-off points for the 6-59-month age group as a whole and found wide heterogeneity and a common discrepancy from the 2001 guideline. A survey in India ^14^ with an intention and approach (but not method) similar to the WHO proposes cut-off points significantly lower than the 2001 and 2024 guidelines.

There are also studies supporting the existence of discrepancies with the 2001 guideline according to age ^15^^-^^21^^,^^11^ and altitude ^22^^,^^23^, as well as the heterogeneity of these discrepancies ^24^^,^^9^^,^^11^, but not their magnitude. Indications that altitude adjustment varies with evolutionary adaptation are of particular interest ^25^.

A recent Peruvian study ^26^, whose sample (6-8 months at sea level) is very small and whose representativeness is debatable (children from healthcare centers without documented random selection), included, in addition to Lima, the cities of Arequipa, Cusco, and Puno. Its exponential regression produces a different adjustment to the 2024 guideline. Other age groups are likely to be published in the near future.

Finally, there are alternative adjustment proposals ^27^^-^^30^ which provide very weak support due to their limited representativeness in terms of scope and ability to exclude pathology.

Our interpretation is that the current evidence, while strongly suggesting that the 2024 guideline applies corrections in the appropriate direction, is not sufficient to support it as the optimal and universal hemoglobin value. The most important concerns are the representativeness of the reference populations, the selection and exclusion criteria, the smoothness of the age adjustment function, and the heterogeneity between and within different countries.

While it is highly desirable to base decisions on scientifically sound evidence, in public health practice this does not always seem possible. Decision-making under uncertainty is necessary, but reasonable precautions must be taken to manage strategic and tactical failures in a timely manner. The signs that a correction is needed are growing stronger, but definitive evidence on the magnitude of that correction may take several years of research and will almost certainly result in further modifications.

Evidence gaps are challenges for short- and long-term research. Several of the studies that now provide partial evidence could be used as cohort baselines to measure the risk of different outcomes as a function of hemoglobin, age, altitude, and other covariates.

For this research, we applied official definitions ^37^ of the anemia indicator, obtaining annual national prevalence rates using the 2001 guideline that were identical to the official ones ^38^ to three significant digits, except for 2019-2021, when they differed by 0.1 to 0.2. We attribute the remaining discrepancies to complex sample software and minor documentation ambiguities ^37^^-^^39^. The official definitions are based on children who slept overnight the previous day, apply a special weighting for the years 2015 and 2020, apply altitude adjustment from 1000 MASL only from 2022, and convert meters to feet using a factor of 3.3. Our results on the comparison between guidelines are the same as in our preprint ^40^.

Regarding the literature related to our study, the WHO 2024 guideline ^5^^)^ was published in March, and INEI published the annual ENDES data ^32^^)^ in May. Since our preprint ^40^^)^ on May 29, after searching PubMed, BVS, and Google, we have only found three studies comparing the guidelines. The first ^41^^)^ used the SIEN 2012-2017 registry (combined years) for care of children aged 6-23 months in healthcare centers from Metropolitan Lima, finding a clear reduction in prevalence. The second ^42^^)^ used data from ENDES 2023 for women aged 15-49 years, and reported specific differences that varied significantly by region and setting. The third ^43^^)^ used the Demographic and Health Surveys (DHS) in a recent year from 48 countries (including ENDES 2022 from Peru, ENDI 2023 from Ecuador, and EDSA 2016 from Bolivia, countries with large populations at high altitudes) for children aged 6-59 months, finding that prevalence rates varied according to country, setting, and, for the three countries mentioned, subnational region, changing in many cases, but not all, the level of public health problem. These studies did not examine temporal trends or provide statistical evaluation of the summarized results. However, their findings are consistent with ours.

Regarding the practical importance of our findings, it is important to note that the level of the public health problem, using the WHO categories ^1^, changes from severe to moderate in many scenarios, thereby changing the recommendation for mass supplementation. The level of the problem is defined based on the prevalence value and is a different concept from the prevalence of severe, moderate, or mild anemia, which are degrees of severity of the individual diagnosis. Note also that the percentage of children whose diagnosis changes (i.e., the complement of the total predictive value) covers a wide and variable range, thereby changing clinical decisions regarding prevention and treatment ^10^. This variability means that a common, simple rule cannot be applied to all scenarios.

We have examined only the subgroup aged 6-35 months. Similar considerations may apply to other subgroups. We do not address two other critical issues here: etiology ^44^, particularly the actual contribution of iron deficiency, and instruments ^5^. The new guideline should be applied cautiously and gradually, accompanied by a critical review of the available evidence as it emerges. As our opinion, we offer the following recommendations, grouped into three themes:

On the specific issue of the cut-off point: 2024 guideline should be recognized as a provisional correction, not fully supported. Since we found discrepancies in the adjustment method for altitude, we believe it is better to use the equation, not the table, from sea level, and for the age adjustment also use its equation ^7^, even though this is not specified in the guideline, since the staggered changes would not be consistent with what is known about pathophysiology. These calculations should be interpreted only as a mitigation with remaining limitations. Take into account that the interpretation at medium and high altitudes is fragile. Periods with parallel indicators should be considered in the communication scenario, taking care to ensure transparency in methods and explanations.

When it comes to controlling childhood anemia in public health: given the unresolved uncertainty, the surveillance to detect under-coverage, leakage, and inequities (not only in hemoglobin indicators, but also in their determinants, including social, environmental, and interventions), as well as the consequences of anemia and its interventions (including adverse effects) should be strengthened. The strategy should be reviewed, reanalyzing the evidence with a margin for intuitive decisions, in order to modify the target populations, the set of interventions, and the priorities for rapid studies, if this is the technical consensus.

For the topic of preventive and restorative care for individual health: recognize that medical practice must apply its criteria to each unique patient. Individual management guidelines and algorithms should be updated ^10^, taking into account that changes are more noticeable in children under 24 months of age and/or at altitudes above 2000 MASL. Review health service management plans, insurance coverage, and care procedures regulated by the state, particularly MINSA and EsSalud.

It should be noted that the 2024 guideline is conceptually retroactive: it logically implies recognizing that we, the technical community, were partially mistaken in our diagnosis and subsequent management of anemia, both individually and at the population level. This reflection does not seek to question the scientific method, but rather to remind us that science is not a static entity; it evolves with criticism.

The ENDES data source has significant strengths: national and subnational representativeness, continuity for more than a decade, and the ecological, economic, social, and cultural diversity of Peru. It also has some limitations such as: (a) reweighting, which we do not believe affects the calculations, separated by year and without modeling; (b) design changes ^31^ in the ENDES triennial cycles, such as the definition of strata, which were redefined (we cannot exclude with certainty changes associated with design modifications); (c) accuracy, particularly in subgroups with smaller samples, so that small differences cannot be defined with certainty; (d) the measurement of capillary blood with portable HemoCue hemoglobinometers (mostly model 201); (e) the definition of a single altitude for each cluster, which may include households at quite different altitudes; (f) the limited adjustment of the GLM models on which statistical significance is based; and (g) the small discrepancy with official prevalences.

The conclusions of the study are the following: the new WHO 2024 guideline reduces the national prevalence by 6.3% in Peruvian children between 6 and 35 months of age, with the reduction varying according to administrative region, setting (urban or rural), and calendar year, ranging from -40.6 to 11. The national percentage of children whose diagnosis changes with the new guideline is 11.0%, with varying levels by region, setting, and year, ranging from 0.0 to 40.6. The reported changes in prevalence and individual diagnosis are statistically significant and of great public health importance, in some cases modifying the severity of the problem. The calculation technique, equation (which we recommend) or table, affects the estimated prevalence by a level of -3.8. The literature supports the direction of the correction, but not completely its magnitude.

## References

[1] World Health Organization (2017). Nutritional anaemias: tools for effective prevention and control.

[2] Ministerio de Salud (MINSA) (2024). Decreto Supremo N.° 002-2024-SA Aprobar el Plan Multisectorial para la Prevención y Reducción de la Anemia Materno Infantil en el Perú. Periodo 2024-2030, que como Anexo forma parte integrante del presente Decreto Supremo.

[3] Ochoa Woodell T, Baiocchi Ureta N, Gonzáles Rengifo GF, Huicho Oriundo L, Marull Espinoza MV, Universidad Peruana Cayetano Heredia, Grupo de Trabajo de Anemia (2020). Anemia Infantil: Retos para su Control en el Perú. Informe Final.

[4] Colegio Médico del Perú (2023). Informe del Seminario - La Problemática de la Anemia Infantil en el Perú: Situación y Retos, una nueva perspectiva.

[5] World Health Organization (2024). Guideline on haemoglobin cutoffs to define anaemia in individuals and populations.

[6] United Nations Children's Fund, United Nations University, World Health Organization (2001). Iron Deficiency Anaemia Assessment, Prevention, and Control: A guide for programme managers.

[7] Braat S, Fielding KL, Han J, Jackson VE, Zaloumis S, Xu JXH (2024). Haemoglobin thresholds to define anaemia from age 6 months to 65 years: estimates from international data sources. Lancet Haematol.

[8] Kanu FA, Jefferds MED, Williams AM, Addo OY, Suchdev PS, Sharma AJ (2023). Association between Hemoglobin and Elevation among School-aged Children A Verification of Proposed Adjustments. Am J Clin Nutr.

[9] Sharma AJ, Addo OY, Mei Z, Suchdev PS (2019). Reexamination of hemoglobin adjustments to define anemia altitude and smoking. Ann N Y Acad Sci.

[10] Perú, Ministerio de Salud (MINSA) (2024). Resolución Ministerial N.° 251-2024-MINSA Aprobar la NTS N° 213-MINSA/DGIESP-2024, Norma Técnica de Salud: Prevención y control de la anemia por deficiencia de hierro en el niño y la niña, adolescentes, mujeres en edad fértil, gestantes y puérperas, que como Anexo forma parte integrante de la presente Resolución Ministerial y que se publica en la sede digital del Ministerio de Salud.

[11] Jorgensen JM, Crespo-Bellido M, Dewey KG (2019). Variation in hemoglobin across the life cycle and between males and females. Ann N Y Acad Sci.

[12] Beutler E, Waalen J (2006). The definition of anemia: what is the lower limit of normal of the blood hemoglobin concentration?. Blood.

[13] Addo OY, Yu EX, Williams AM, Young MF, Sharma AJ, Mei Z (2021). Evaluation of Hemoglobin Cutoff Levels to Define Anemia Among Healthy Individuals. JAMA Netw Open.

[14] Sachdev HS, Porwal A, Acharya R, Ashraf S, Ramesh S, Khan N (2021). Haemoglobin thresholds to define anaemia in a national sample of healthy children and adolescents aged 1-19 years in India a population-based study. Lancet Glob Health.

[15] Hawkins WW, Speck E, Leonard VG (1954). Variation of the hemoglobin level with age and sex. Blood.

[16] Burman D (1972). Haemoglobin levels in normal infants aged 3 to 24 months, and the effect of iron. Arch Dis Child.

[17] Dallman PR, Siimes MA (1979). Percentile curves for hemoglobin and red cell volume in infancy and childhood. J Pediatr.

[18] Yip R, Johnson C, Dallman PR (1984). Age-related changes in laboratory values used in the diagnosis of anemia and iron deficiency. Am J Clin Nutr.

[19] Emond AM, Hawkins N, Pennock C, Golding J (1996). Haemoglobin and ferritin concentrations in infants at 8 months of age. Arch Dis Child.

[20] Sherriff A, Emond A, Hawkins N, Golding J (1999). Haemoglobin and ferritin concentrations in children aged 12 and 18 months ALSPAC Children in Focus Study Team. Arch Dis Child.

[21] Jopling J, Henry E, Wiedmeier SE, Christensen RD (2009). Reference ranges for hematocrit and blood hemoglobin concentration during the neonatal period data from a multihospital health care system. Pediatrics.

[22] Hurtado A, Merino C, Delgado E (1945). Influence of anoxemia on the hemopoietic activity. Arch Intern Med.

[23] Centers for Disease Control (1989). CDC criteria for anemia in children and childbearing-aged women. MMWR Morb Mortal Wkly Rep.

[24] Gassmann M, Mairbäurl H, Livshits L, Seide S, Hackbusch M, Malczyk M (2019). The increase in hemoglobin concentration with altitude varies among human populations. Ann N Y Acad Sci.

[25] Mairbäurl H, Gassmann M, Muckenthaler MU (2020). Geographical ancestry affects normal hemoglobin values in high-altitude residents. J Appl Physiol Bethesda Md 1985.

[26] Aparco JP, Santos-Antonio G, Bautista-Olortegui W, Alvis-Chirinos K, Velarde-Delgado P, Hinojosa-Mamani P (2023). Estado de hierro y propuesta de ajuste de hemoglobina por altitud en niños de 6 a 8 meses residentes en Lima, Arequipa, Cusco y Puno. Rev Peru Med Exp Salud Pública.

[27] Dirren H, Logman MH, Barclay DV, Freire WB (1994). Altitude correction for hemoglobin. Eur J Clin Nutr.

[28] Bartolo-Marchena M, Pajuelo-Ramírez J, Obregón-Cahuay C, Bonilla Untiveros C, Racacha-Valladares E, Bravo-Rebatta F (2017). Propuesta de factor de corrección a las mediciones de hemoglobina por pisos altitudinales en menores de 6 a 59 meses de edad, en el Perú. An Fac Med.

[29] Ocas-Córdova S, Tapia V, Gonzales GF (2018). Hemoglobin Concentration in Children at Different Altitudes in Peru Proposal for [Hb] Correction for Altitude to Diagnose Anemia and Polycythemia. High Alt Med Biol.

[30] Accinelli RA, Leon-Abarca JA (2020). Age and altitude of residence determine anemia prevalence in Peruvian 6 to 35 months old children. PloS One.

[31] Perú, Instituto Nacional de Estadística e Informática (INEI) (2023). Perú: Encuesta Demográfica y de Salud Familiar - ENDES 2022.

[32] Perú, Instituto Nacional de Estadística e Informática (2024). Perú: Encuesta Demográfica y de Salud Familiar - ENDES 2023.

[33] Perú, Instituto Nacional de Estadística e Informática (2009). Perú: Estimaciones y Proyecciones de Población, 1950-2050.

[34] Nestel P (2002). Adjusting Hemoglobin Values in Program Surveys.

[35] Lumley T (2010). Complex surveys: a guide to analysis using R..

[36] R Core Team (2024). R: A language and environment for statistical computing.

[37] Perú, Instituto Nacional de Estadística e Informática (2024). Metodología del indicador de anemia en niñas y niños de 6 a 59 meses.

[38] Perú Instituto Nacional de Estadística e Informática (2024). PERÚ: Indicadores de Resultados de los Programas Presupuestales 2023 - Encuesta Demográfica y de Salud Familiar.

[39] Perú Instituto Nacional de Estadística e Informática (2008). Encuesta Demográfica y de Salud Familiar - Sintaxis de Programas de los Indicadores Identificados en los Programas Estratégicos - ENDES Línea de Base.

[40] Campos Sánchez M, Cordero Muñoz L, Velásquez Hurtado E, Baiocchi Ureta N, Miranda Cuadros M, Sánchez Griñán MI (2024). New WHO guideline on the definition of anemia: implications for 6-35 months old children in Peru 2009-2023. medRxiv.

[41] Vásquez-Velásquez C, Tapia V, Gonzales GF (2024). La nueva guía sobre los puntos de corte de la hemoglobina para definir anemia en individuos y poblaciones. Rev Soc Peru Med Interna.

[42] Hernández-Vásquez A, Guerra Valencia J, Vargas-Fernández R (2024). ¿Cuánto ha cambiado la prevalencia de anemia en mujeres peruanas con los criterios de la OMS 2024? análisis de la ENDES 2023. Rev Peru Med Exp Salud Pública.

[43] Hernández-Vásquez A, Vargas-Fernández R, Guerra Valencia J (2024). Change in the prevalence of anemia in children aged 6-59 months using the new World Health Organization 2024 criteria. Ann N Y Acad Sci.

[44] Engle-Stone R, Aaron GJ, Huang J, Wirth JP, Namaste SM, Williams AM (2017). Predictors of anemia in preschool children Biomarkers Reflecting Inflammation and Nutritional Determinants of Anemia (BRINDA) project. Am J Clin Nutr.

