# Supplementary material for: New world health organization guideline on anemia cut-off points: implications for children aged 6-35 months in Peru
Source: Rev Peru Med Exp Salud Publica. 2025 Jun 12;42(2):115–25. doi: 10.17843/rpmesp.2025.422.14028 (PMC12377884; doi:10.17843/rpmesp.2025.422.14028)
Supplement: Supplementary material. — Available in the electronic version of the RPMESP. [file rpmesp-42-02-14028-s001.pdf]

# Suplemento para el artículo
# Nueva directriz OMS sobre los puntos de corte de anemia: implicancias en niños 6-35 meses de edad
# manuscrito Rev Per Med Exp 2025
# preprint medRxiv doi 10.1101/2024.05.28.24308069

M Campos, L Cordero, E Velásquez, N Baiocchi, M Miranda, M Sánchez-Griñán, W Valdivia

2025-Feb-16

## Contents

**Fuentes de ENDES** **2**

**GLM: Métodos y Diagnósticos** **4**
Métodos . . . 4
Resultados . . . 6
Dataset: . . . 6
Model: DNOR~HV007 m5 Prevalence Proportion Difference vs Year . . . 7
Model: DNOR~HV023 m6 Prevalence Proportion Difference vs Region . . . 14
Model: DCAM~HV023 m7 Diagnostic Change Proportion vs Region . . . 21
Model: DNOR~ m1 Prevalence Proportion Difference vs covars . . . 28
Model: DCAM~ m2 Diagnostic Change Proportion vs covars . . . 35
Comentarios . . . 42
Referencias . . . 42

**Programa R** **43**

**P2025021620.R MCNut24.R 2025-Feb-17 vipermcs@gmail.com et al 2021-Mar-08** **43**

## Fuentes de ENDES

```r
L=LALT$RZO
print(L,n=nrow(L))
```

```
## # A tibble: 18 x 8
##    VV007  NN06  NYA9  NDIN CIC   MMU         DMU                     DNO
##    <dbl> <int> <dbl> <int> <chr> <chr>       <chr>                   <chr>
##  1  2005  2764     0    NA 04-08 <NA>        <NA>                    <NA>
##  2  2007  2902     0    NA 04-08 <NA>        <NA>                    <NA>
##  3  2008  5081     0    NA 04-08 <NA>        <NA>                    <NA>
##  4  2009 10704  5260  4394 09-11 CNPV 2007   Estratificado Bietápico <NA>
##  5  2010  9812  4906  4434 09-11 CNPV 2007   Estratificado Bietápico <NA>
##  6  2011  9582  4848  4481 09-11 CNPV 2007   Estratificado Bietápico <NA>
##  7  2012 10231  5100  4677 12-14 CNPV 2007   Estratificado Bietápico +Hogares Secundarios
##  8  2013  9574  4726  4333 12-14 CNPV 2007   Estratificado Bietápico +Hogares Secundarios
##  9  2014 10222  4997  4586 12-14 CNPV 2007   Estratificado Bietápico +Hogares Secundarios
## 10  2015 25527 13169 12182 15-17 CNPV+SISFOH Equilibrado             <NA>
## 11  2016 22682 11530 10660 15-17 CNPV+SISFOH Equilibrado             <NA>
## 12  2017 22483 11578 10970 15-17 CNPV+SISFOH Equilibrado             <NA>
## 13  2018 24288 11963 11377 18-20 CNPV+SISFOH Equilibrado             <NA>
## 14  2019 22143 11070 10535 18-20 CNPV+SISFOH Equilibrado             <NA>
## 15  2020 13529  6508  6171 18-20 CNPV+SISFOH Equilibrado             Pandemia, Parte Virtual
## 16  2021 22741 11613 11044 21-23 CNPV 2017   Equilibrado             <NA>
## 17  2022 22424 11274 10722 21-23 CNPV 2017   Equilibrado             <NA>
## 18  2023 21464 10612 10145 21-23 CNPV 2017   Equilibrado             <NA>
```

```r
 ## VV007: año de la encuesta
 ## NN06: Totales: Registrados en el Archivo ENDES.
 ## NYA9: Elegibles: Entre 6 y 35m (o sin edad) 2009-2023.
 ## NDIN: Incluidos: Entre 6-35m, con dato de Hb y Encuestado en Año.
 ## CIC: Ciclo trienal ENDES
 ## MMU: Marco Muestral (CNPV: Censo Nacional de Población y Vivienda. SISFOH: Sistema de Focalización 
L=LALT$FTES
print(L,n=nrow(L))
```

```
## # A tibble: 45 x 7
##    Name                                 Length Date                ZipFile            SrcFile
##    <chr>                                 <dbl> <dttm>              <chr>              <chr>
##  1 238-Modulo64/RECH0.SAV              3202509 2010-05-07 18:25:00 238-Modulo64.zip   RECH0.SAV
##  2 238-Modulo64/RECH1.SAV              6111249 2010-05-07 18:25:00 238-Modulo64.zip   RECH1.SAV
##  3 238-Modulo74/RECH6.SAV              1794791 2010-05-07 18:26:00 238-Modulo74.zip   RECH6.SAV
##  4 XX-Modulo64/RECH0.SAV               3256931 2011-05-16 12:16:00 260-Modulo64.zip   XX-Modulo64/R
##  5 XX-Modulo64/RECH1.SAV               5890762 2011-05-16 12:21:00 260-Modulo64.zip   XX-Modulo64/R
##  6 XX-Modulo74/RECH6.SAV               1620072 2011-05-16 14:35:00 260-Modulo74.zip   XX-Modulo74/R
##  7 RECH0.sav                           3299169 2012-03-01 12:01:00 290-Modulo64.zip   RECH0.sav
##  8 RECH1.sav                           5730261 2012-02-22 15:07:00 290-Modulo64.zip   RECH1.sav
##  9 Modulo 74/RECH6.sav                 1570695 2012-02-24 08:32:00 290-Modulo74.zip   Modulo 74/REC
## 10 323-Modulo64/RECH0.sav              3411395 2013-05-06 09:55:00 323-Modulo64.zip   RECH0.sav
## 11 323-Modulo64/RECH1.sav              5995740 2013-05-06 09:27:00 323-Modulo64.zip   RECH1.sav
## 12 323-Modulo74/RECH6.sav              1678029 2013-05-06 09:29:00 323-Modulo74.zip   RECH6.sav
## 13 407-Modulo64/RECH0.SAV              3330294 2014-01-15 09:38:00 407-Modulo64.zip   RECH0.SAV
## 14 407-Modulo64/RECH1.SAV              6848904 2014-05-23 09:50:00 407-Modulo64.zip   RECH1.SAV
## 15 407-Modulo74/RECH6.SAV              1580494 2014-02-04 09:17:00 407-Modulo74.zip   RECH6.SAV
## 16 441-Modulo64/RECH0.SAV              4180882 2015-04-10 16:19:00 441-Modulo64.zip   RECH0.SAV
```

```
## 17 441-Modulo64/RECH1.SAV             7282744 2015-01-19 17:25:00 441-Modulo64.zip   RECH1.SAV
## 18 441-Modulo74/RECH6.SAV             1676876 2015-01-19 16:25:00 441-Modulo74.zip   RECH6.SAV
## 19 504-Modulo64/RECH0.SAV             4649335 2016-04-01 10:19:00 504-Modulo64.zip   RECH0.SAV
## 20 504-Modulo64/RECH1.SAV            11052340 2016-04-21 12:01:00 504-Modulo64.zip   RECH1.SAV
## 21 504-Modulo74/RECH6.SAV             4148488 2016-02-17 11:00:00 504-Modulo74.zip   RECH6.SAV
## 22 RECH1.SAV                          9098150 2017-02-22 14:33:00 548-Modulo64.zip   RECH1.SAV
## 23 RECH0.SAV                          6072117 2017-05-25 08:40:00 548-Modulo64.zip   RECH0.SAV
## 24 RECH6.SAV                          3688856 2017-02-22 14:20:00 548-Modulo74.zip   RECH6.SAV
## 25 Modulo64/RECH0.SAV                 7049948 2018-03-06 08:44:00 605-Modulo64.zip   Modulo64/RECH
## 26 Modulo64/RECH1.SAV                 9008086 2018-01-29 14:23:00 605-Modulo64.zip   Modulo64/RECH
## 27 Modulo74/RECH6.SAV                 3645776 2018-01-29 14:20:00 605-Modulo74.zip   Modulo74/RECH
## 28 Modulo64/RECH0.SAV                 6522619 2019-05-21 08:20:00 638-Modulo64.zip   Modulo64/RECH
## 29 Modulo64/RECH1.SAV                 9126166 2019-05-16 14:24:00 638-Modulo64.zip   Modulo64/RECH
## 30 Modulo74/RECH6.SAV                 3907036 2019-04-10 11:27:00 638-Modulo74.zip   Modulo74/RECH
## 31 Modulo64/RECH0.SAV                 7196426 2020-04-30 00:15:00 691-Modulo64.zip   Modulo64/RECH
## 32 Modulo64/RECH1.sav                10357707 2020-04-30 00:16:00 691-Modulo64.zip   Modulo64/RECH
## 33 Modulo74/RECH6.SAV                 3712317 2020-04-30 00:27:00 691-Modulo74.zip   Modulo74/RECH
## 34 739-Modulo1629/Modulo1629/RECH0.sav  7552302 2021-05-21 11:18:00 739-Modulo1629.zip Modulo1629/RE
## 35 739-Modulo1629/Modulo1629/RECH1.sav 11333299 2021-05-21 11:18:00 739-Modulo1629.zip Modulo1629/RE
## 36 739-Modulo1638/Modulo1638/RECH6.sav  2398239 2021-05-21 11:18:00 739-Modulo1638.zip Modulo1638/RE
## 37 760-Modulo1629/RECH0.SAV           7415366 2022-04-07 14:35:00 760-Modulo1629.zip RECH0.SAV
## 38 760-Modulo1629/RECH1.SAV          11394219 2022-04-07 11:29:00 760-Modulo1629.zip RECH1.SAV
## 39 760-Modulo1638/RECH6.SAV           3806877 2022-04-07 11:29:00 760-Modulo1638.zip RECH6.SAV
## 40 786-Modulo1629/RECH0.sav           7180746 2023-05-14 19:41:00 786-Modulo1629.zip RECH0.sav
## 41 786-Modulo1629/RECH1.sav          11344299 2023-05-14 19:41:00 786-Modulo1629.zip RECH1.sav
## 42 786-Modulo1638/RECH6.sav           3754933 2023-05-14 19:41:00 786-Modulo1638.zip RECH6.sav
## 43 910-Modulo1629/RECH0_2023.sav      7814129 2024-05-22 16:53:00 910-Modulo1629.zip RECH0_2023.sa
## 44 910-Modulo1629/RECH1_2023.sav     11310955 2024-05-22 16:53:00 910-Modulo1629.zip RECH1_2023.sa
## 45 910-Modulo1638/RECH6_2023.sav      3601597 2024-05-22 16:53:00 910-Modulo1638.zip RECH6_2023.sa
```

```r
L=LALT$FTEX
print(L,n=nrow(L))
```

```
## # A tibble: 45 x 8
##     FSEQ FNME  YRPA   NCOL   NROW    SIZEB MD5                              SHA256
##    <dbl> <chr> <chr> <int>  <int>    <dbl> <chr>                            <chr>
##  1     1 RECH0 S2023    44  37486  7814129 775cfd3e0068308da0421cc382c4207e 06cbd89b01525308a308a6ef
##  2     2 RECH0 S2022    44  37350  7180746 fa6b5f920fd77d1d8bb5341f3d4f100c 4c32dd5e76c7b12576521707
##  3     3 RECH0 S2021    44  37479  7415366 79a2eda2d6bbb60b61b257b79d173390 7cd557ef13ca554f085b3ab2
##  4     4 RECH0 S2020    44  38083  7552302 17653df5f8499711d481026cc86f5b25 944bf80d62902d27bccdc055
##  5     5 RECH0 S2019    44  37474  7196426 5199c8879ab8c3d2c5483e2e9f2fabb6 f059e61573d58c576dbf61c4
##  6     6 RECH0 S2018    42  37486  6522619 c2a8b7f3c6cb46402a710c1998eb688e 7d13625fd48af87c8c4aa383
##  7     7 RECH0 S2017    49  36595  7049948 8aff6a4131dd3d544cc9706ecdd493dc e5ed8fcfd3d838fc00149945
##  8     8 RECH0 S2016    45  36647  6072117 c970d8aaffa9bc52c66201173df5cfa6 0c021025f4d46ebbdffbf870
##  9     9 RECH0 S2015    42  36692  4649335 4499daa2d96e052a9de2a7e56d043687 3a9e3bb039fba4a8b6654937
## 10    10 RECH0 S2014    43  30361  4180882 f8ce87f75a2b0e95dcd5a69efea68429 722127959699afa7ce1e1496
## 11    11 RECH0 S2013    41  28324  3330294 baa1668dd5dac22f4006b7987fefc12f e3604879f59b7dd119beec8d
## 12    12 RECH0 S2012    41  28376  3411395 aaab0343849d2faf55fabcebac325741 bba739f8097a7ef57a1cbeef
## 13    13 RECH0 S2011    40  27709  3299169 3d7fd75c33437f793b91c97f6eb57bc6 5af4dc906c12c18fcb65ba8c
## 14    14 RECH0 S2010    40  27756  3256931 f03250fc9ae9ccc888256b6a2f7c1336 44da5a414ac59b10e8e3e937
## 15    15 RECH0 S2009    40  27709  3202509 116309fdac2fc23f7f196fff5065ee9a 4624027c94c7041eaff3fbf0
## 16    20 RECH1 S2023    36 139278 11310955 76dfc13256b828b67a43e2d6b8ae1acb 3ffa3138690d3b9b9a2dd6e1
## 17    21 RECH1 S2022    36 139618 11344299 79cd05a7aec91031a8e2f31df0ed731a 4de2b10cd3d099637eab9c2c
## 18    22 RECH1 S2021    36 140276 11394219 8b67cbb978d1b129f3b00b9b78381032 005106ab426141ac46601693
## 19    23 RECH1 S2020    36 139653 11333299 5841e6ecc3f0b1f2d1b7a2f75e1a8dd9 c6c4b2b5e281a59dfaf61bc9
```

```
## 20    24 RECH1 S2019    36 143486 10357707 2830661001cb72afedda9a38486889aa 609342a8bed77cfee500af44
## 21    25 RECH1 S2018    35 147629  9126166 38d61dace11019c1bc531eca31c1e585 163f987191db35dadd3ae560
## 22    26 RECH1 S2017    47 140598  9008086 6f2317e40a94894b7f2b52b6d01a7da3 c07cc30a3d43d3ddb332f3f5
## 23    27 RECH1 S2016    47 142006  9098150 99c9d33f727e6058c8263ad21662d8db 9476da3339294511c5ce4b67
## 24    28 RECH1 S2015    48 151267 11052340 76ae24fe7a09324367cacdb5158ae19a ed749f9aaf6f470349cfb8d5
## 25    29 RECH1 S2014    50 108536  7282744 1b2550fdfffa77c4e3c97dfb72307afe f8879275af9a8a556f80bb3b
## 26    30 RECH1 S2013    52  99097  6848904 45d5c8e9dacb7668ffe4a9f1c0e34e16 67d389687c99df3ead98dd77
## 27    31 RECH1 S2012    41 103211  5995740 1ddf1db7e43a4c61139e078145579bed 91f8774d1268d02357d53998
## 28    32 RECH1 S2011    41  98662  5730261 bd13cd493e30912d23c9e268f5dd9953 189618561bfb2722913e7036
## 29    33 RECH1 S2010    41 101409  5890762 6209590bf1c6d0588b99b8d1443fe51c 7e0c1c77cdfc1b487540c150
## 30    34 RECH1 S2009    41 105225  6111249 9181386946c88e4b07f9e7d51ef956f3 ee6275bdc8c52dd990f77800
## 31    39 RECH6 S2023    41  21464  3601597 7e791931457cc4b66c4cd14af6dc2343 c05326a0ee8e6e8f5351ca1b
## 32    40 RECH6 S2022    41  22424  3754933 3b33f9aefb61a1745c62b46d541d08b2 1999b0696a22d2c29afb26b6
## 33    41 RECH6 S2021    41  22741  3806877 46a53fc8b76a00d2fd62e3d07c3ed790 b262af180cf28a125e2f2ae3
## 34    42 RECH6 S2020    42  13529  2398239 d2495ee04e75e738f14d15e4b615dafa 3187131916141bb0802118cf
## 35    43 RECH6 S2019    41  22143  3712317 4fcbba923990683e84a80719c349a735 6a541f9a20f8ea27b0462015
## 36    44 RECH6 S2018    42  24288  3907036 612aa9250ff7e1ead6d9d234f15ef6a4 6487f5bfc721ef3845459103
## 37    45 RECH6 S2017    44  22483  3645776 1bad4cf43c518b079b5e06c1b6e89f43 555f9b74dd3ff9e1e8c64cb4
## 38    46 RECH6 S2016    42  22682  3688856 4fe3aa2fff6cec5573329ec9d6a200c9 8a8780a39a4ec9e9b717d533
## 39    47 RECH6 S2015    42  25527  4148488 d6a610c2f0a6f7a6634903ebbfcf1908 1d82936aa2d30187e228d38c
## 40    48 RECH6 S2014    42  10222  1676876 d414ac633cb4f6d72ad33afbed2a2d14 facb47881aeeda51eccdd98c
## 41    49 RECH6 S2013    43   9574  1580494 ed0098386ab03627bfca85074db94224 53c20ff91bd1c2bc1cb7c526
## 42    50 RECH6 S2012    42  10231  1678029 dc4e494c151a3aca5e49d699716de2da 97d775abc82a2f3a7a565469
## 43    51 RECH6 S2011    42   9582  1570695 a2b780df59ddfd256b9d4e16b55aceb6 a00f2630cb5f79d69e47d137
## 44    52 RECH6 S2010    42   9812  1620072 c2b3907e8297b51560c561b4bcaa150d cf0cd22f87e7b07b1213d177
## 45    53 RECH6 S2009    42  10704  1794791 4c14ae82bf40af5a53883add587f12f6 57b866b4c317e044916438e6
```

# GLM: Métodos y Diagnósticos

## Métodos

Dado que las normas 2001 y 2024 han especificado diferencias precisas en sus puntos de corte, las prevalencias y cantidades relacionadas diferirán en cantidades directamente relacionadas al grupo de edad y nivel de altitud, así como a la distribución de la población por edad y altitud y su cercanía a los puntos de corte.

Por consiguiente, no habría razón de ensayar la significancia estadística de las diferencias por edad o altitud.

Tampoco habría razón para ensayar las diferencias según características, como region administrative, ámbito urbano o año, de las cuales se conoce que tienen cambios demográficos a lo largo del tiempo.

Lo que sería de mayor interés es la estimación de las magnitudes para esas diferencias. Ese es el punto principal del artículo y la prepublicación.

No obstante, a solicitud de los revisores, hemos efectuado algunos ensayos estadísticos para complementar la información sobre el tema.

Tenemos un indicador principal de resultado ('outcome') que es la diferencia entre la prevalencia calculada según ambas normas. La variable de nivel individual es la diferencia entre el diagnóstico de anemia diagnosis (como un valor dicótomo 0|1) con ambas normas. Por consiguiente, la variable es tricótoma, con valores posibles -1, 0 y 1.

También temenos un indicador secundario de resultado que es la proporción de niños cuyo diagnóstico de anemia difiere con ambas normas. La variable de nivel individual-es dicótoma.

Con ligeras diferencias, la secuencia de análisis para cada indicador ha sido como sigue:

(a) Estimación de los valores promedio según año calendario de la encuesta.

- Estimación ponderada de las medias con su intervalo de 95% de confianza.
- Prueba t ponderada de una sola muestra para la hipótesis de cero en cada categoría anual.
- Prueba de razón de verosimilitud ponderada para la hipótesis de homogeneidad entre categorías.
- Modelo lineal generalizado ponderado para la hipótesis bivariable, similar al paso (c).

(b) Estimación de los valores promedio según región administrativa.

- Similar al paso (a).

(c) Modelamiento de los valores promedio según año, región administrativa y ámbito urbano/rural y sus interacciones.

- Modelo lineal generalizado ponderado, familia gaussiana para la variable tricótoma y familia binomial para la variable dicótoma.
- Exploración y evaluación de los residuos cuantílicos para evaluar los diagnósticos de los modelos.

La fuente de datos es la información consolidada de la encuesta aleatoria continua ENDES a lo largo de 15 años.

Para este artículo se han aplicado los criterios de inclusión y las fórmulas oficialmente publicadas por el INEI.

El software usado fue R 4.4.2 con los paquetes tidyverse, survey y DHARMa.

## Resultados

**Dataset:**

```
print(dim(m1$data))
```

```
## [1] 120711     11
```

```
print(head(m1$data))
```

```
##   DNOR DCAM HV007    HV023 BURB2 VV007        HV022 HV001           HHID    W1RF .survey.prob.weigh
## 1    0    0  2023 Amazonas     0  2023        RURAL     1      000102201 0.09059              0.153
## 2    0    0  2023 Amazonas     0  2023        RURAL     1      000102801 0.09059              0.153
## 3    0    0  2023 Amazonas     0  2023        RURAL     1      000108001 0.09059              0.153
## 4    0    0  2023 Amazonas     0  2023        RURAL     1      000108801 0.09059              0.153
## 5    0    0  2023 Amazonas     0  2023        RURAL     1      000108801 0.09059              0.153
## 6    0    0  2023 Amazonas     0  2023 RESTO URBANO     6      000613701 0.10731              0.181
```

### Model: DNOR~HV007 m5 Prevalence Proportion Difference vs Year

```
print(m5$call)
```

### Model Summary

```
## svyglm(formula = DNOR ~ factor(HV007), design = x, family = gaussian)
```

```
print(m8$call)
```

```
## svyglm(formula = DNOR ~ 1, design = x, family = gaussian)
```

```
LALT$RMRES|>filter(MOD=='m5')
```

```
## # A tibble: 1 x 9
##   null.deviance df.null    AIC    BIC deviance df.residual   nobs MOD     MCFAR2
##           <dbl>   <int>  <dbl>  <dbl>    <dbl>       <dbl>  <int> <chr>    <dbl>
## 1        12777.  120710 71474. 12957.   12770.       34107 120711 m5    0.000572
```

```
print(anova(object=m5,object2=m8))
```

### Model Tests

```
## Working (Rao-Scott+F) LRT for factor(HV007)
##  in svyglm(formula = DNOR ~ factor(HV007), design = x, family = gaussian)
## Working 2logLR =  32.509 p= 0.0138
## (scale factors:  2.1 1.8 1.7 1.6 1.4 1.1 0.95 0.79 0.77 0.67 0.59 0.55 0.53 0.52 1.6e-05 );  denomina
```

```
L=GST0(ll=2009:2023,yv='DNOR',bv='HV007')
print(L,n=nrow(L))
```

```
## # A tibble: 15 x 2
##    SVLevel  STTestP
##      <int>    <dbl>
##  1    2009 9.58e-21
##  2    2010 1.02e-22
##  3    2011 3.34e-25
##  4    2012 2.33e-19
##  5    2013 1.58e-19
##  6    2014 6.98e-23
##  7    2015 5.27e-49
##  8    2016 8.06e-35
##  9    2017 5.34e-42
## 10    2018 4.98e-36
## 11    2019 1.88e-46
## 12    2020 2.83e-38
## 13    2021 3.58e-81
## 14    2022 2.77e-43
## 15    2023 2.97e-36
```

```
print(LALT$RMTER|>filter(MOD=='m5'),n=100)
```

### Model Terms

```
## # A tibble: 16 x 6
##    term               estimate std.error statistic  p.value MOD
```

```
##    <chr>                  <dbl>     <dbl>     <dbl>    <dbl> <chr>
##  1 (Intercept)         0.0718     0.00752     9.54  1.56e-21 m5
##  2 factor(HV007)2010 -0.00274    0.0102     -0.269 7.88e- 1 m5
##  3 factor(HV007)2011 -0.0115     0.00942    -1.22  2.23e- 1 m5
##  4 factor(HV007)2012 -0.0132     0.00988    -1.33  1.83e- 1 m5
##  5 factor(HV007)2013 -0.00456    0.0105     -0.434 6.64e- 1 m5
##  6 factor(HV007)2014 -0.00423    0.0101     -0.418 6.76e- 1 m5
##  7 factor(HV007)2015 -0.0107     0.00856    -1.26  2.09e- 1 m5
##  8 factor(HV007)2016 -0.0187     0.00864    -2.16  3.07e- 2 m5
##  9 factor(HV007)2017 -0.00983    0.00876    -1.12  2.62e- 1 m5
## 10 factor(HV007)2018 -0.0208     0.00853    -2.44  1.46e- 2 m5
## 11 factor(HV007)2019 -0.0103     0.00863    -1.20  2.31e- 1 m5
## 12 factor(HV007)2020  0.000946   0.00932     0.101 9.19e- 1 m5
## 13 factor(HV007)2021  0.00972    0.00859     1.13  2.58e- 1 m5
## 14 factor(HV007)2022 -0.0128     0.00862    -1.48  1.39e- 1 m5
## 15 factor(HV007)2023 -0.0169     0.00867    -1.95  5.06e- 2 m5
## 16 factor(HV007)2024 -0.0718     0.00752    -9.54  1.56e-21 m5
```

```
L=GDX8(mm=m5,NDSIM=30)
```

DNOR gaussian

**Model Diagnostics (quantile residuals)**

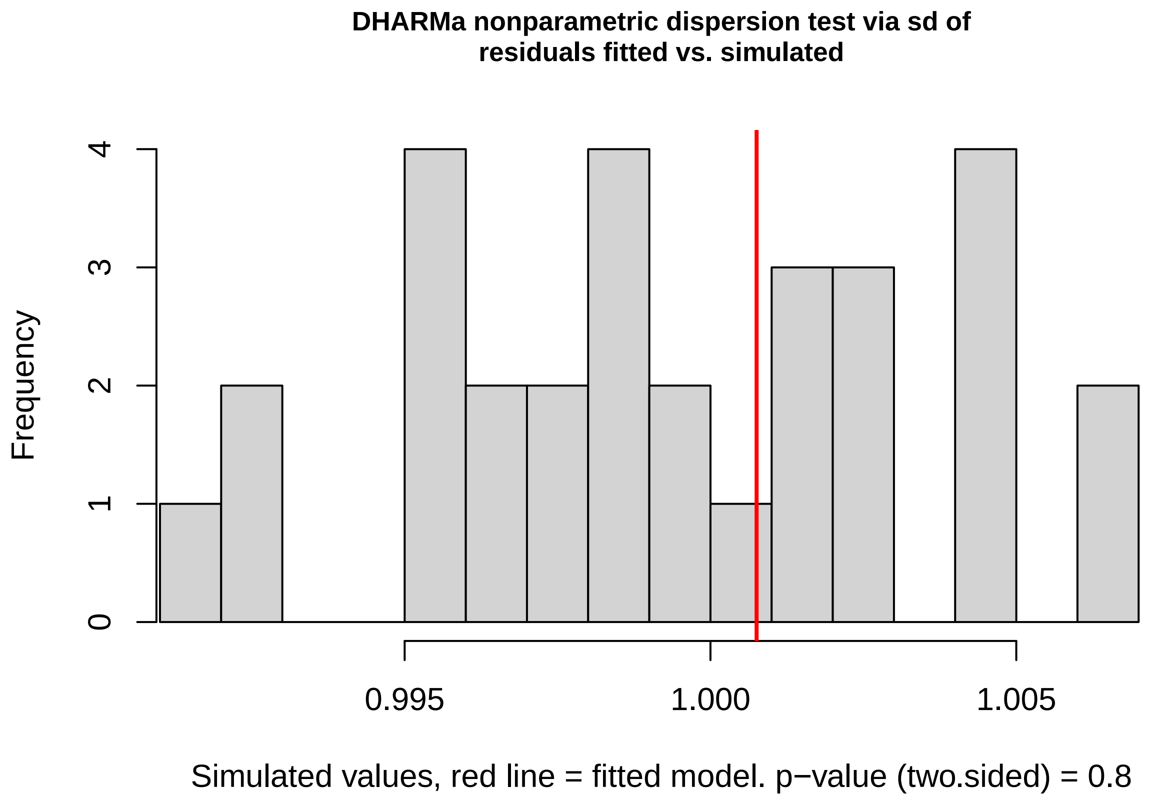

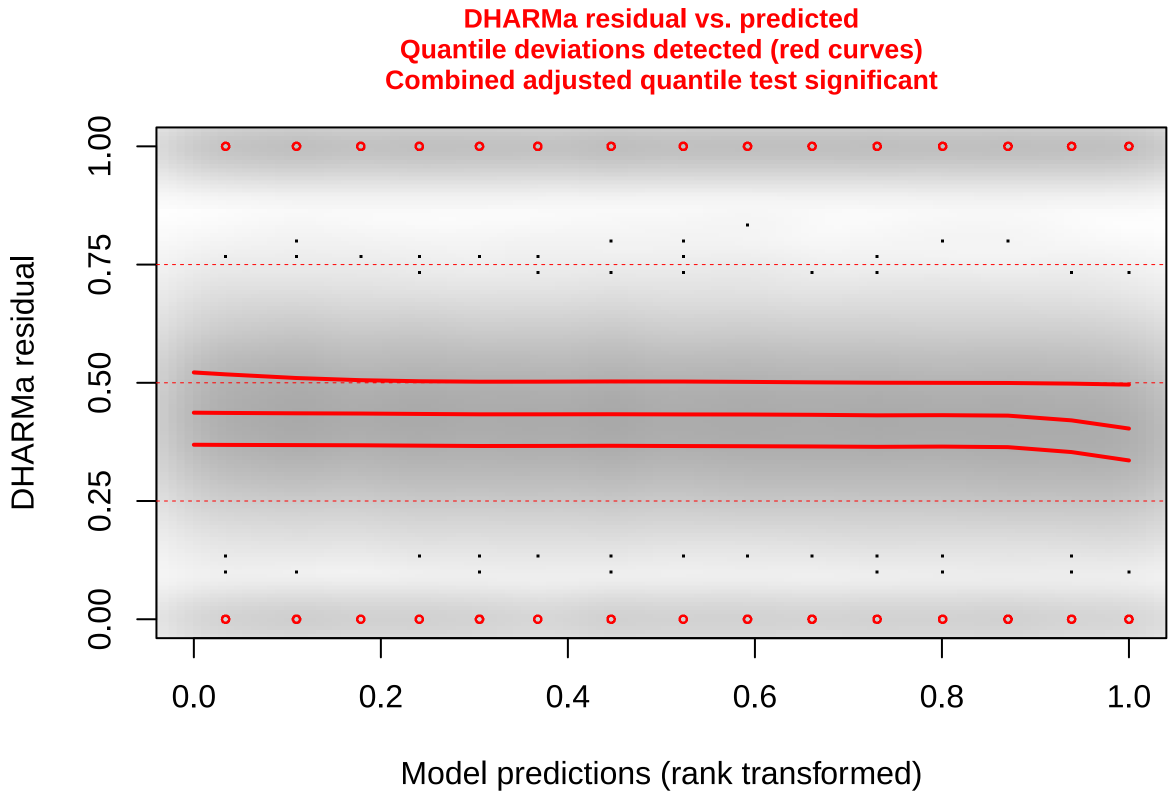
DHARMa residual vs. predicted
Quantile deviations detected (red curves)
Combined adjusted quantile test significant
DHARMa residual
0.00
0.25
0.50
0.75
1.00
0.0
0.2
0.4
0.6
0.8
1.0
Model predictions (rank transformed)

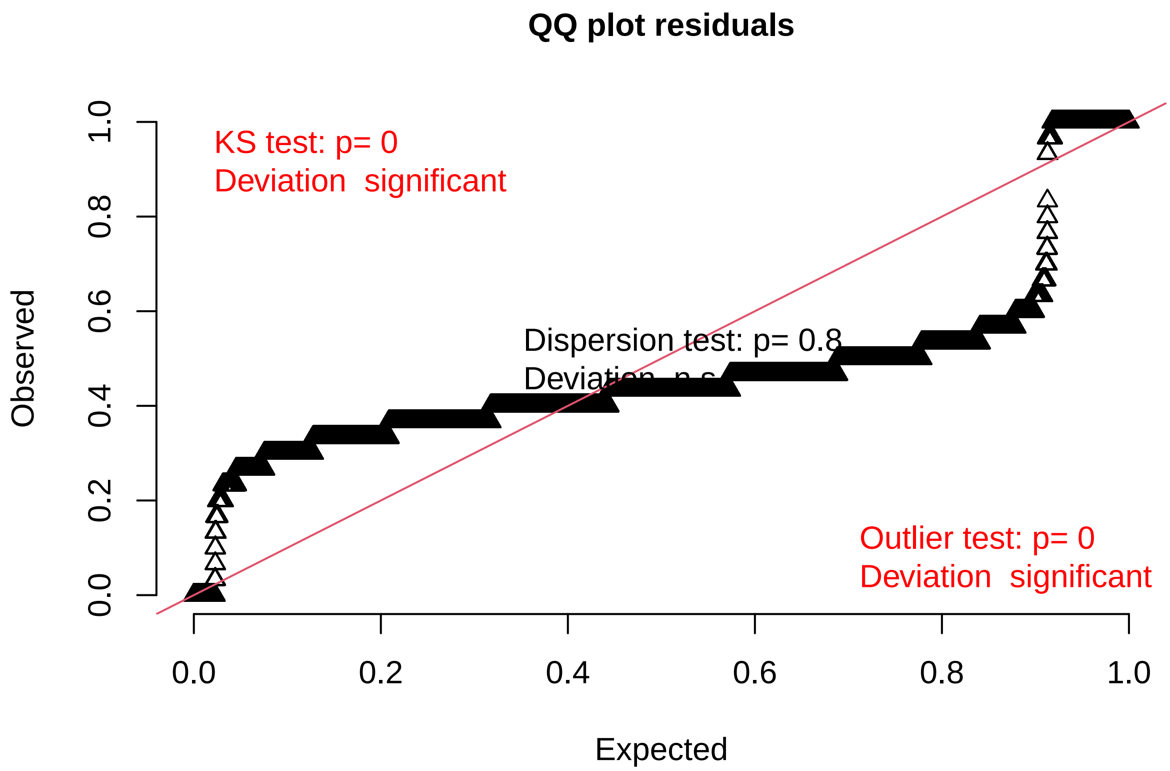
QQ plot residuals
KS test: p= 0
Deviation significant
Dispersion test: p= 0.8
Deviation n.s.
Outlier test: p= 0
Deviation significant
Observed
Expected
0.0
0.2
0.4
0.6
0.8
1.0

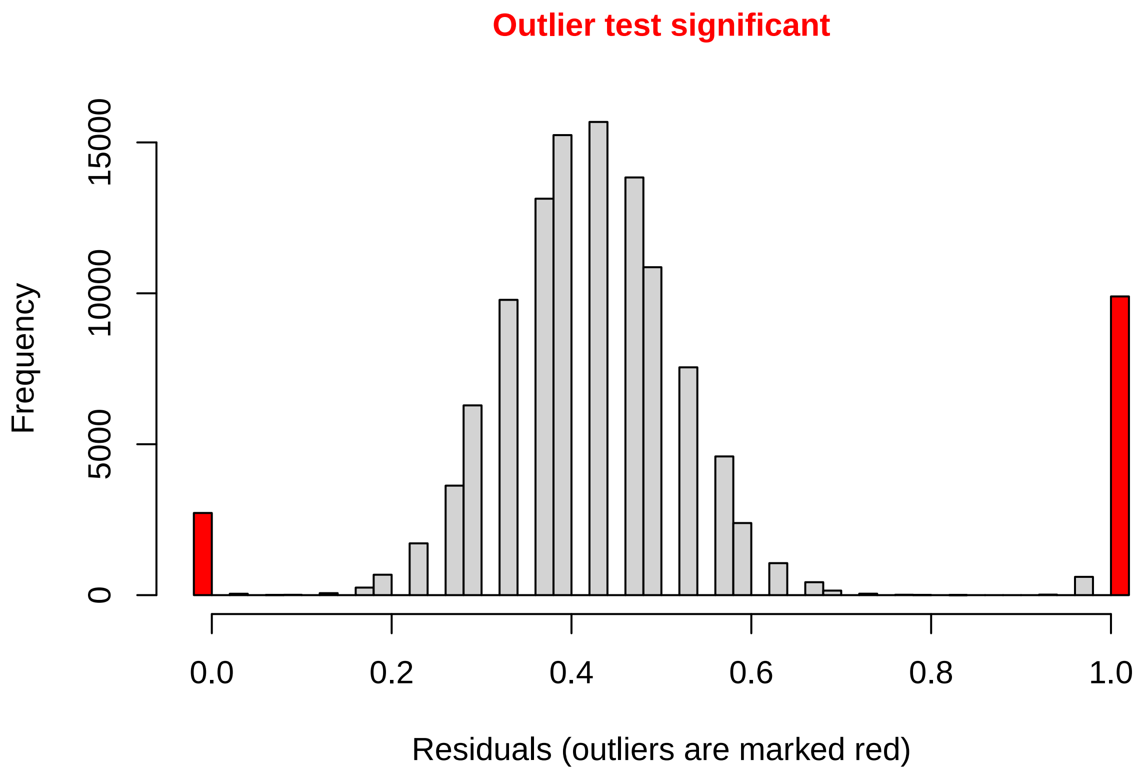


```r
print(L,n=nrow(L))
```

```
## # A tibble: 8 x 3
##   IPAR        XVAL DSC
##   <chr>      <dbl> <chr>
## 1 PDISP1 8    e-  1 p, Nonpar Dispersion test
## 2 PQGAM2 0         p, Combined Adjusted Quantile test
## 3 POSKS3 0         p, Asymptotic One-Sample Kolmogorov-Smirnov test
## 4 POUTB4 4.94e-324 p, Outlier Exact Binomial test
## 5 PEXPE4 6.45e-  2 Expected Proportion of Outliers
## 6 POBSE4 1.05e-  1 Observed Proportion of Outliers
## 7 PLLOW4 1.03e-  1 Lower CL Proportion of Outliers
## 8 PLUPP4 1.06e-  1 Upper CL Proportion of Outliers
```

### Model: DNOR~HV023 m6 Prevalence Proportion Difference vs Region

```
print(m6$call)
```

### Model Summary

```
## svyglm(formula = DNOR ~ factor(HV023), design = x, family = gaussian)
```

```
print(m8$call)
```

```
## svyglm(formula = DNOR ~ 1, design = x, family = gaussian)
```

```
LALT$RMRES|>filter(MOD=='m6')
```

```
## # A tibble: 1 x 9
##   null.deviance df.null    AIC    BIC deviance df.residual   nobs MOD   MCFAR2
##           <dbl>   <int>  <dbl>  <dbl>    <dbl>       <dbl>  <int> <chr>  <dbl>
## 1        12777.  120710 68167. 12717.   12424.       34098 120711 m6    0.0276
```

```
print(anova(object=m6,object2=m8))
```

### Model Tests

```
## Working (Rao-Scott+F) LRT for factor(HV023)
##  in svyglm(formula = DNOR ~ factor(HV023), design = x, family = gaussian)
## Working 2logLR =  2334.3 p= <2e-16
## (scale factors:  2.6 2.1 1.9 1.7 1.5 1.5 1.4 1.3 1.2 0.99 0.95 0.91 0.9 0.84 0.7 0.65 0.65 0.57 0.44
```

```
L=GST0(ll=levels(d$HV023),yv='DNOR',bv='HV023')
print(L,n=nrow(L))
```

```
## # A tibble: 25 x 2
##    SVLevel             STTestP
##    <chr>                 <dbl>
##  1 "Amazonas"        3.02e-  8
##  2 "Ancash"          1.96e- 38
##  3 "Apurimac"        3.58e- 47
##  4 "Arequipa"        2.70e-  2
##  5 "Ayacucho"        6.09e- 20
##  6 "Cajamarca"       8.05e-  1
##  7 "Callao"          6.78e- 24
##  8 "Cusco"           8.97e- 57
##  9 "Huancavelica"    1.38e- 79
## 10 " Huanuco"        8.64e- 11
## 11 " Ica"            9.28e-  7
## 12 " Junin"          1.13e- 30
## 13 " La Libertad"    2.48e- 35
## 14 " Lambayeque"     3.36e- 32
## 15 " Lima"           5.75e- 52
## 16 " Loreto"         5.45e- 54
## 17 " Madre de Dios" 1.29e-  7
## 18 " Moquegua"       9.61e-  1
## 19 " Pasco"          1.46e- 40
## 20 " Piura"          4.69e- 28
## 21 " Puno"           4.02e-134
## 22 " San Martin"     1.44e-  1
```

```
## 23 " Tacna"         3.84e-  1
## 24 " Tumbes"        6.00e- 32
## 25 " Ucayali"       2.37e- 36
```

```r
print(LALT$RMTER|>filter(MOD=='m6'),n=100)
```

### Model Terms

```
## # A tibble: 25 x 6
##    term                          estimate std.error statistic   p.value MOD
##    <chr>                            <dbl>     <dbl>     <dbl>     <dbl> <chr>
##  1 (Intercept)                    -0.0278   0.00498     -5.58 2.48e-  8 m6
##  2 factor(HV023)Ancash             0.105    0.00760     13.8  3.93e- 43 m6
##  3 factor(HV023)Apurimac           0.134    0.00862     15.6  1.45e- 54 m6
##  4 factor(HV023)Arequipa           0.0150   0.00760      1.98 4.81e-  2 m6
##  5 factor(HV023)Ayacucho           0.0895   0.00830     10.8  4.75e- 27 m6
##  6 factor(HV023)Cajamarca          0.0263   0.00779      3.37 7.49e-  4 m6
##  7 factor(HV023)Callao             0.104    0.00890     11.7  2.47e- 31 m6
##  8 factor(HV023)Cusco              0.158    0.00921     17.2  6.75e- 66 m6
##  9 factor(HV023)Huancavelica       0.205    0.00993     20.6  4.77e- 94 m6
## 10 factor(HV023) Huanuco           0.0776   0.00910      8.53 1.52e- 17 m6
## 11 factor(HV023) Ica               0.0512   0.00689      7.44 1.06e- 13 m6
## 12 factor(HV023) Junin             0.106    0.00829     12.8  1.66e- 37 m6
## 13 factor(HV023) La Libertad       0.106    0.00788     13.4  4.33e- 41 m6
## 14 factor(HV023) Lambayeque        0.0916   0.00724     12.7  1.30e- 36 m6
## 15 factor(HV023) Lima              0.0804   0.00604     13.3  2.76e- 40 m6
## 16 factor(HV023) Loreto            0.102    0.00675     15.1  2.43e- 51 m6
## 17 factor(HV023) Madre de Dios     0.0559   0.00726      7.69 1.51e- 14 m6
## 18 factor(HV023) Moquegua          0.0274   0.00807      3.40 6.68e-  4 m6
## 19 factor(HV023) Pasco             0.156    0.0105      14.8  1.21e- 49 m6
## 20 factor(HV023) Piura             0.0828   0.00698     11.9  2.25e- 32 m6
## 21 factor(HV023) Puno              0.294    0.0106      27.8  7.76e-168 m6
## 22 factor(HV023) San Martin        0.0206   0.00700      2.94 3.30e-  3 m6
## 23 factor(HV023) Tacna             0.0331   0.00786      4.20 2.62e-  5 m6
## 24 factor(HV023) Tumbes            0.0995   0.00775     12.8  1.21e- 37 m6
## 25 factor(HV023) Ucayali           0.0883   0.00682     12.9  3.05e- 38 m6
```

```
L=GDX8(mm=m6,NDSIM=30)
```

DNOR gaussian

**Model Diagnostics (quantile residuals)**

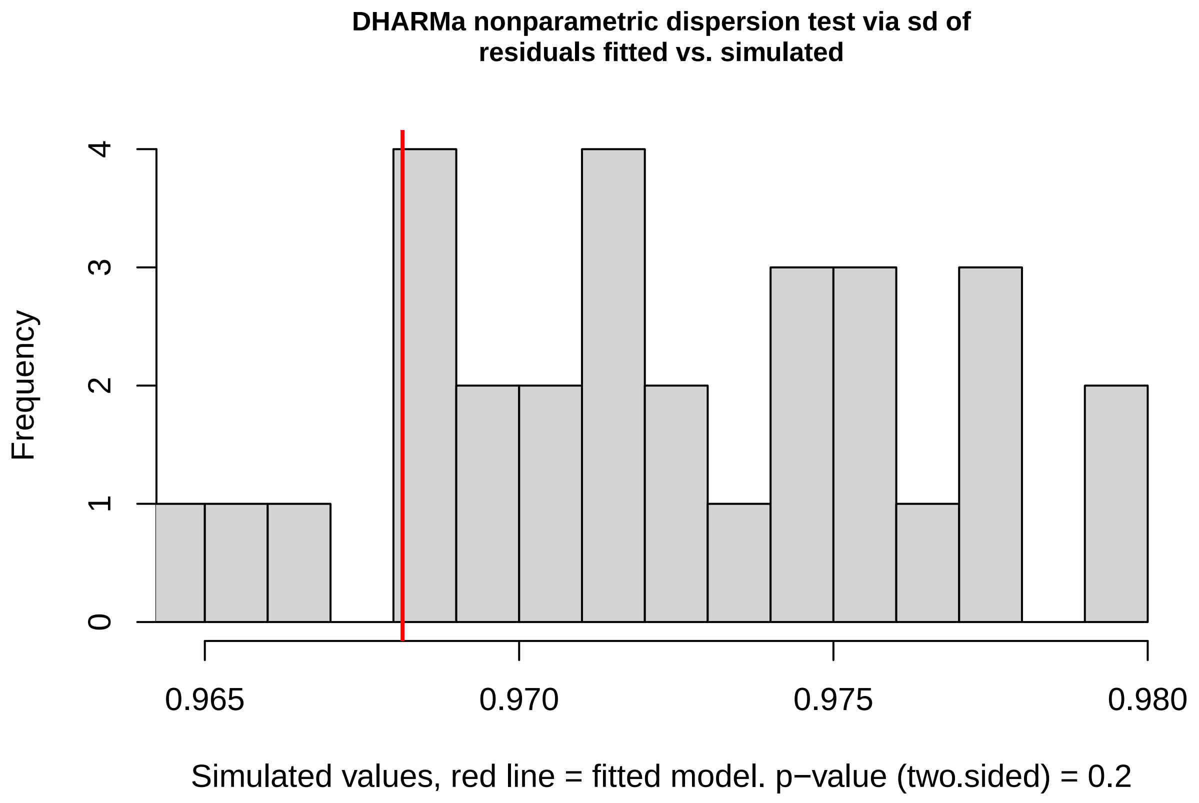

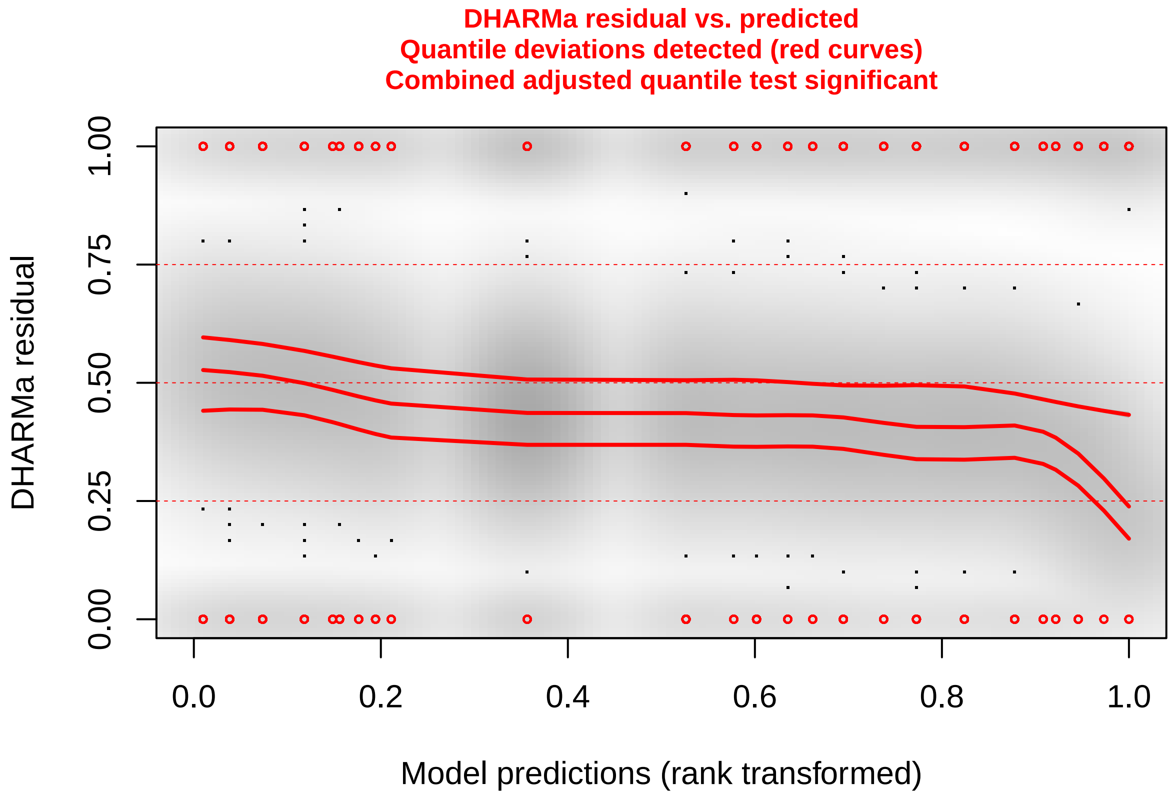
DHARMa residual vs. predicted
Quantile deviations detected (red curves)
Combined adjusted quantile test significant
DHARMa residual
0.00
0.25
0.50
0.75
1.00
0.0
0.2
0.4
0.6
0.8
1.0
Model predictions (rank transformed)

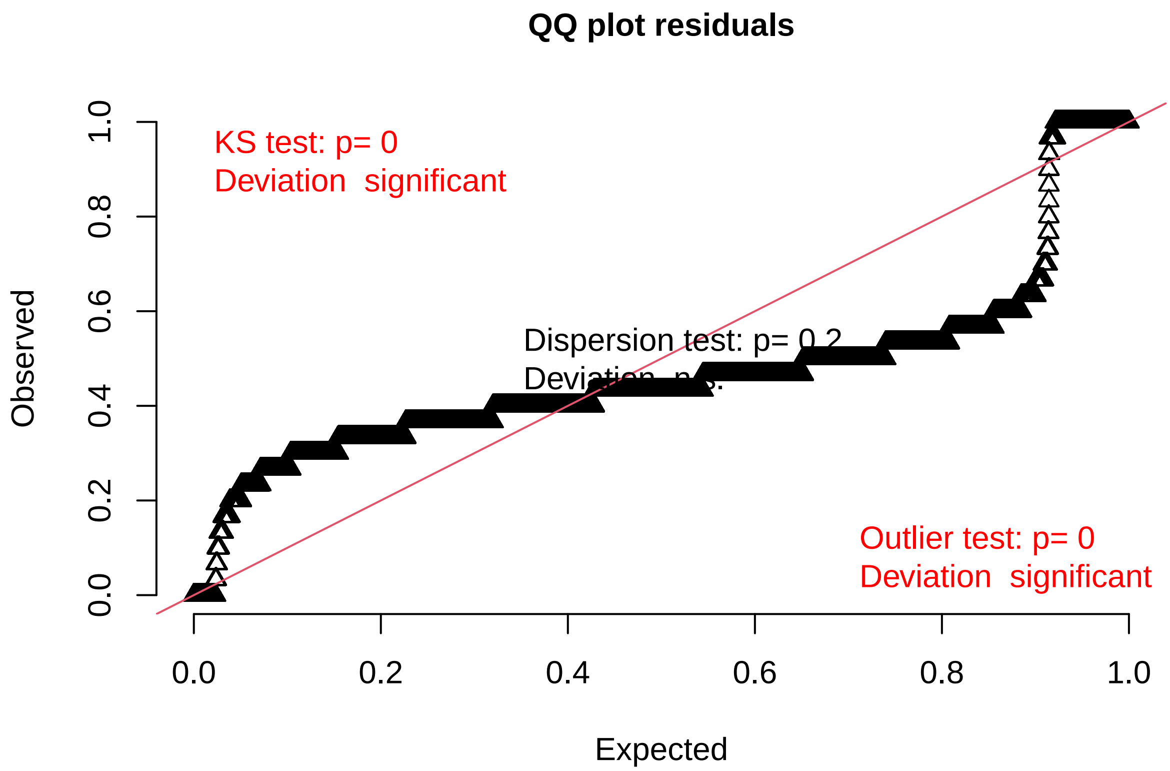

QQ plot residuals
KS test: p= 0
Deviation significant
Dispersion test: p= 0.2
Deviation n.s.
Outlier test: p= 0
Deviation significant
Observed
0.0
0.2
0.4
0.6
0.8
1.0
Expected
0.0
0.2
0.4
0.6
0.8
1.0

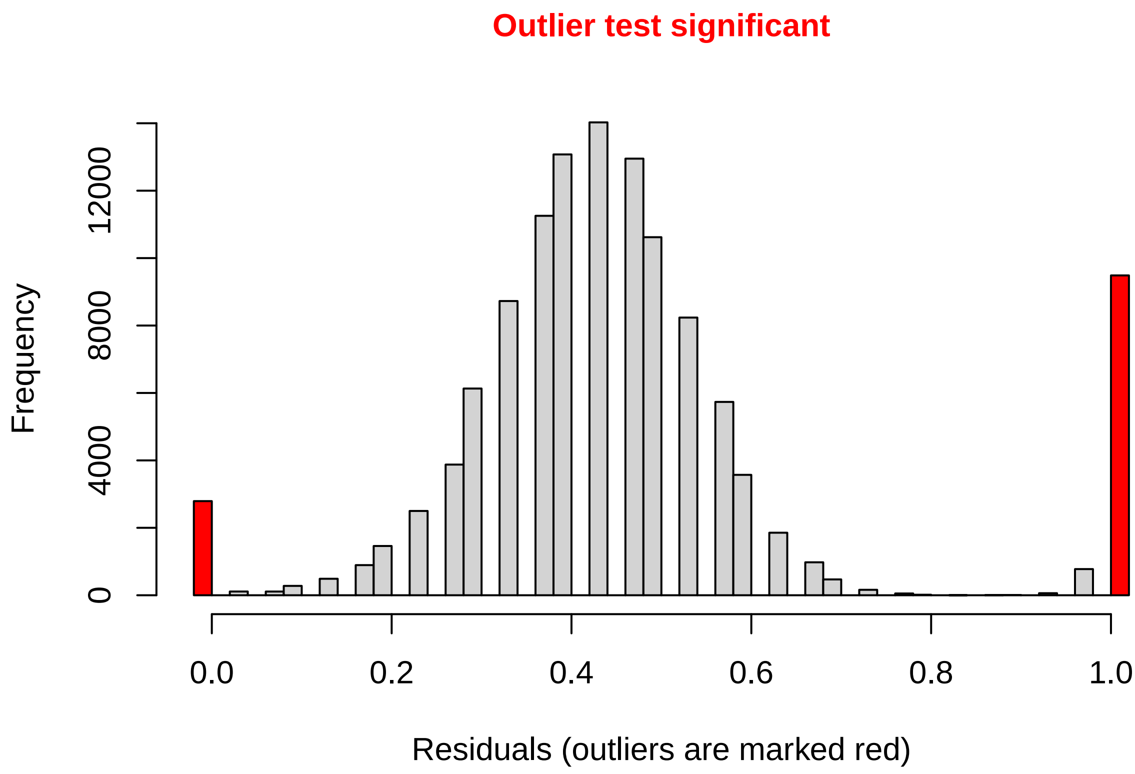


```r
print(L,n=nrow(L))
```

```
## # A tibble: 8 x 3
##   IPAR        XVAL DSC
##   <chr>      <dbl> <chr>
## 1 PDISP1 2   e-  1 p, Nonpar Dispersion test
## 2 PQGAM2 0         p, Combined Adjusted Quantile test
## 3 POSKS3 0         p, Asymptotic One-Sample Kolmogorov-Smirnov test
## 4 POUTB4 4.94e-324 p, Outlier Exact Binomial test
## 5 PEXPE4 6.45e-  2 Expected Proportion of Outliers
## 6 POBSE4 1.02e-  1 Observed Proportion of Outliers
## 7 PLLOW4 1.00e-  1 Lower CL Proportion of Outliers
## 8 PLUPP4 1.03e-  1 Upper CL Proportion of Outliers
```

### Model: DCAM~HV023 m7 Diagnostic Change Proportion vs Region

```r
print(m7$call)
```

### Model Summary

```
## svyglm(formula = DCAM ~ factor(HV023), design = x, family = binomial)
```

```r
print(m9$call)
```

```
## svyglm(formula = DCAM ~ 1, design = x, family = binomial)
```

```r
LALT$RMRES|>filter(MOD=='m7')
```

```
## # A tibble: 1 x 9
##   null.deviance df.null    AIC    BIC deviance df.residual   nobs MOD   MCFAR2
##           <dbl>   <int>  <dbl>  <dbl>    <dbl>       <dbl>  <int> <chr>  <dbl>
## 1        83576.  120710 82213. 82443.   82150.       34098 120711 m7    0.0171
```

```r
print(anova(object=m7,object2=m9))
```

### Model Tests

```
## Working (Rao-Scott+F) LRT for factor(HV023)
##  in svyglm(formula = DCAM ~ factor(HV023), design = x, family = binomial)
## Working 2logLR =  1081.9 p= <2e-16
## (scale factors:  2.7 2.2 2 1.8 1.5 1.5 1.3 1.2 1.1 1.1 1 0.93 0.8 0.73 0.71 0.66 0.54 0.47 0.44 0.35
```

```r
L=GSTO(ll=levels(d$HV023),yv='DCAM',bv='HV023')
print(L,n=nrow(L))
```

```
## # A tibble: 25 x 2
##    SVLevel            STTestP
##    <chr>                <dbl>
##  1 "Amazonas"       1.15e- 71
##  2 "Ancash"         7.49e- 70
##  3 "Apurimac"       1.61e- 89
##  4 "Arequipa"       1.56e- 58
##  5 "Ayacucho"       2.23e- 76
##  6 "Cajamarca"      2.83e- 51
##  7 "Callao"         4.45e- 29
##  8 "Cusco"          2.19e- 77
##  9 "Huancavelica"   8.27e- 91
## 10 " Huanuco"       3.61e- 78
## 11 " Ica"           1.34e- 77
## 12 " Junin"         1.49e- 86
## 13 " La Libertad"   1.32e- 62
## 14 " Lambayeque"    1.49e- 81
## 15 " Lima"          1.53e-160
## 16 " Loreto"        1.25e- 99
## 17 " Madre de Dios" 2.33e- 75
## 18 " Moquegua"      6.07e- 58
## 19 " Pasco"         7.29e-113
## 20 " Piura"         9.99e- 95
## 21 " Puno"          4.39e-143
## 22 " San Martin"    5.64e- 78
```

```
## 23 " Tacna"         1.69e- 51
## 24 " Tumbes"        1.82e- 75
## 25 " Ucayali"       3.06e-109
```

```
print(LALT$RMTER|>filter(MOD=='m7'),n=100)
```

### Model Terms

```
## # A tibble: 25 x 6
##    term                            estimate std.error statistic  p.value MOD
##    <chr>                              <dbl>     <dbl>     <dbl>    <dbl> <chr>
##  1 (Intercept)                     -2.33       0.0573  -40.7    0        m7
##  2 factor(HV023)Ancash              0.196      0.0825    2.38   1.73e- 2 m7
##  3 factor(HV023)Apurimac            0.524      0.0779    6.73   1.73e-11 m7
##  4 factor(HV023)Arequipa           -0.0513     0.0861   -0.595  5.52e- 1 m7
##  5 factor(HV023)Ayacucho            0.312      0.0810    3.85   1.17e- 4 m7
##  6 factor(HV023)Cajamarca          -0.0589     0.0896   -0.658  5.11e- 1 m7
##  7 factor(HV023)Callao              0.0644     0.112     0.576  5.65e- 1 m7
##  8 factor(HV023)Cusco               0.596      0.0817    7.29   3.10e-13 m7
##  9 factor(HV023)Huancavelica        0.859      0.0795   10.8    3.28e-27 m7
## 10 factor(HV023) Huanuco            0.387      0.0807    4.80   1.60e- 6 m7
## 11 factor(HV023) Ica                0.0244     0.0796    0.306  7.59e- 1 m7
## 12 factor(HV023) Junin              0.388      0.0783    4.95   7.35e- 7 m7
## 13 factor(HV023) La Libertad        0.168      0.0852    1.97   4.87e- 2 m7
## 14 factor(HV023) Lambayeque         0.150      0.0788    1.90   5.73e- 2 m7
## 15 factor(HV023) Lima               0.0417     0.0694    0.601  5.48e- 1 m7
## 16 factor(HV023) Loreto             0.221      0.0749    2.96   3.10e- 3 m7
## 17 factor(HV023) Madre de Dios      0.124      0.0804    1.55   1.22e- 1 m7
## 18 factor(HV023) Moquegua           0.0518     0.0867    0.597  5.50e- 1 m7
## 19 factor(HV023) Pasco              0.872      0.0754   11.6    6.10e-31 m7
## 20 factor(HV023) Piura              0.161      0.0758    2.13   3.35e- 2 m7
## 21 factor(HV023) Puno               1.35       0.0736   18.3    2.33e-74 m7
## 22 factor(HV023) San Martin        -0.00271    0.0791   -0.0343 9.73e- 1 m7
## 23 factor(HV023) Tacna              0.0166     0.0901    0.184  8.54e- 1 m7
## 24 factor(HV023) Tumbes             0.196      0.0809    2.43   1.52e- 2 m7
## 25 factor(HV023) Ucayali            0.212      0.0734    2.89   3.80e- 3 m7
```

```
L=GDX8(mm=m7,NDSIM=30)
```

DCAM binomial

**Model Diagnostics (quantile residuals)**

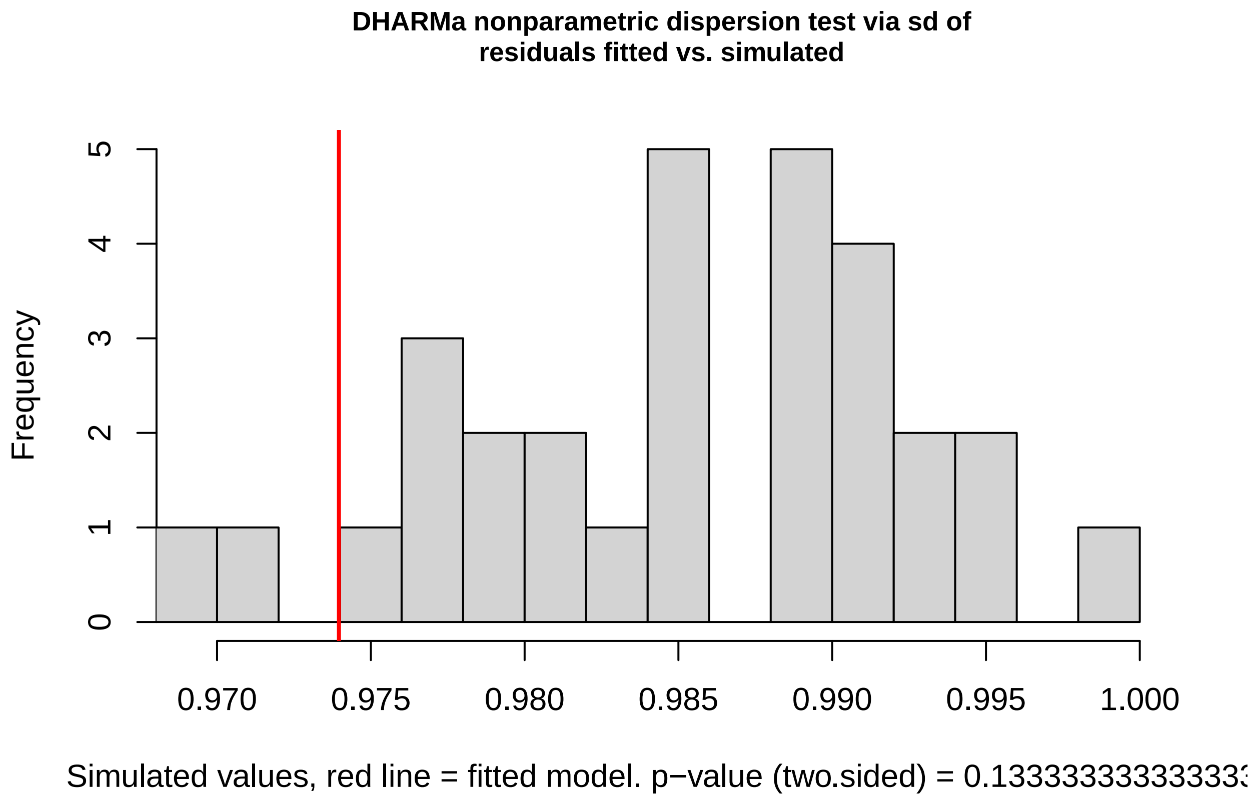

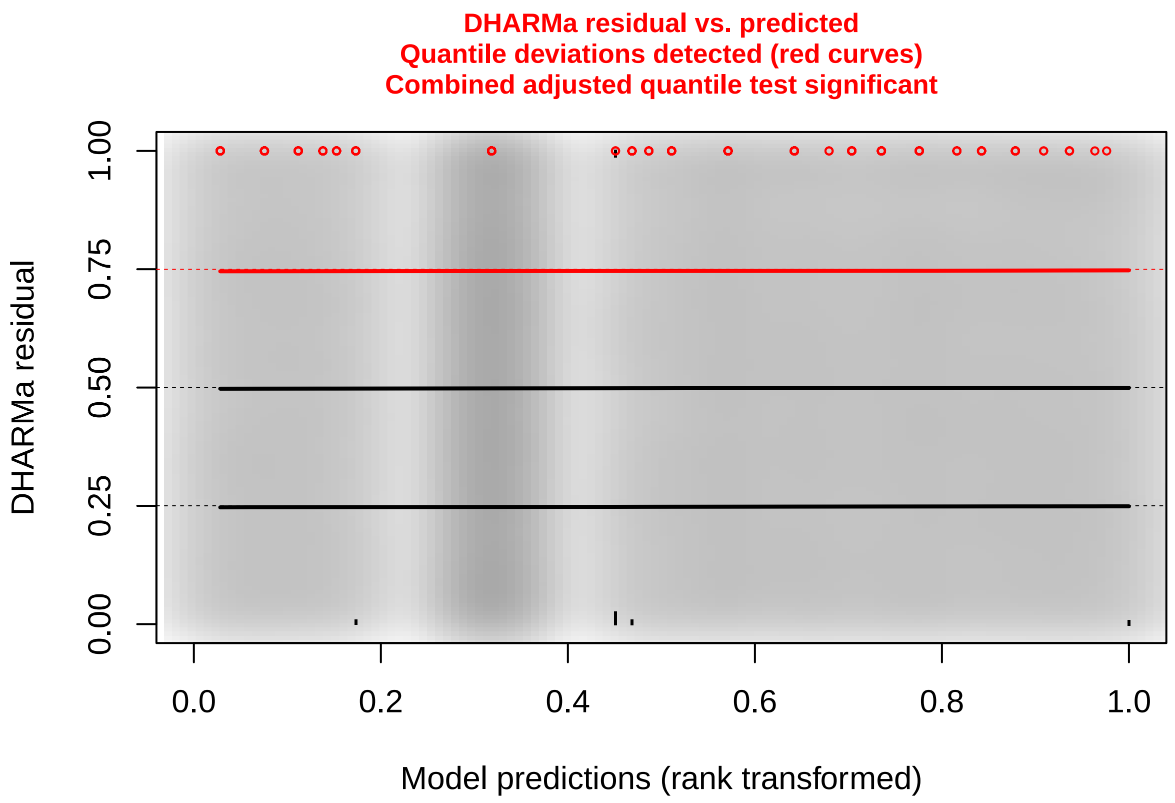
DHARMa residual vs. predicted
Quantile deviations detected (red curves)
Combined adjusted quantile test significant
DHARMa residual
0.00
0.25
0.50
0.75
1.00
0.0
0.2
0.4
0.6
0.8
1.0
Model predictions (rank transformed)

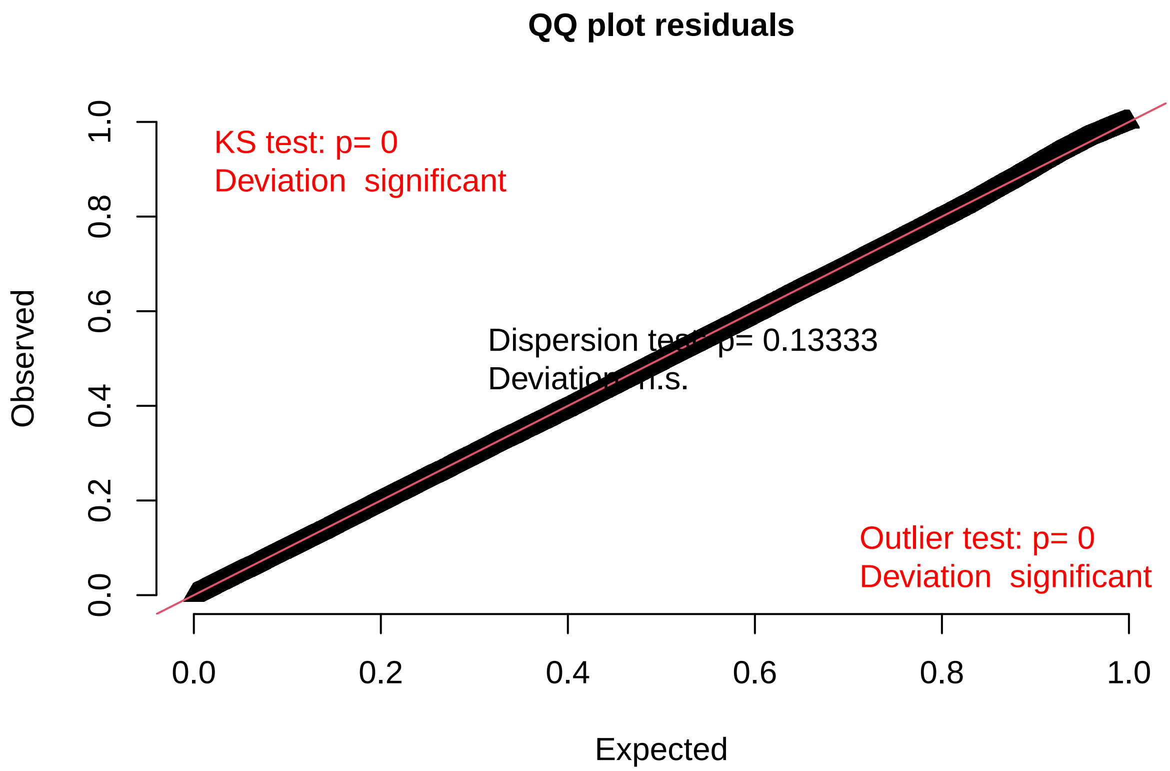
QQ plot residuals
KS test: p= 0
Deviation significant
Dispersion test: p= 0.13333
Deviation n.s.
Outlier test: p= 0
Deviation significant
Observed
Expected
0.0
0.2
0.4
0.6
0.8
1.0

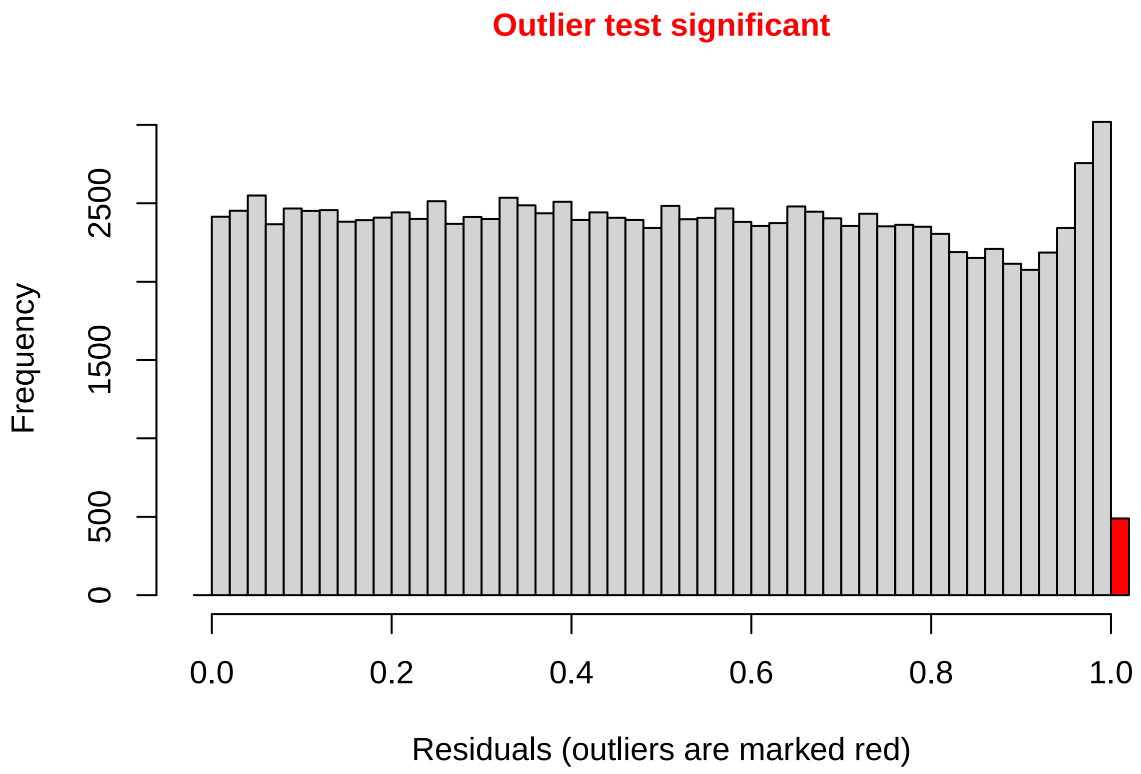


```r
print(L,n=nrow(L))
```

```
## # A tibble: 8 x 3
##   IPAR       XVAL DSC
##   <chr>     <dbl> <chr>
## 1 PDISP1 1.33e- 1 p, Nonpar Dispersion test
## 2 PQGAM2 2.12e- 2 p, Combined Adjusted Quantile test
## 3 POSKS3 7.39e-16 p, Asymptotic One-Sample Kolmogorov-Smirnov test
## 4 POUTB4 6.69e-60 p, Outlier Exact Binomial test
## 5 PEXPE4 6.45e- 2 Expected Proportion of Outliers
## 6 POBSE4 7.64e- 2 Observed Proportion of Outliers
## 7 PLLOW4 7.49e- 2 Lower CL Proportion of Outliers
## 8 PLUPP4 7.79e- 2 Upper CL Proportion of Outliers
```

### Model: DNOR~ m1 Prevalence Proportion Difference vs covars

```r
print(m1$call)
```

### Model Summary

```
## svyglm(formula = DNOR ~ factor(HV007) * factor(HV023) * BURB2,
##     design = x, family = gaussian)
```

```r
LALT$RMRES|>filter(MOD=='m1')
```

```
## # A tibble: 1 x 9
##   null.deviance df.null    AIC    BIC deviance df.residual   nobs MOD   MCFAR2
##           <dbl>   <int>  <dbl>  <dbl>    <dbl>       <dbl>  <int> <chr>  <dbl>
## 1        12777.  120710 68471. 20889.   12266.       33386 120711 m1    0.0400
```

```r
print(LALT$RMFIN$M1$AOV)
```

### Analysis of Deviance

```
## Anova table:  (Rao-Scott LRT)
## svyglm(formula = DNOR ~ factor(HV007), design = x, family = gaussian)
##                                    stats     DEff       df   ddf      p    
## factor(HV007)                       7.31   0.2250  15.0000 34107 0.0138 *  
## factor(HV023)                     352.54   0.1510  24.0000 34083 <2e-16 ***
## BURB2                               0.03   0.1945   1.0000 34082 0.7078    
## factor(HV007):factor(HV023)        61.63   0.1411 337.0000 33745 0.0058 ** 
## factor(HV007):BURB2                 3.11   0.1811  14.0000 33731 0.2508    
## factor(HV023):BURB2                43.78   0.1398  23.0000 33708 <2e-16 ***
## factor(HV007):factor(HV023):BURB2  43.30   0.1252 322.0000 33386 0.2338    
## ---
## Signif. codes:  0 '***' 0.001 '**' 0.01 '*' 0.05 '.' 0.1 ' ' 1
```

```r
print(LALT$RMTER|>filter(MOD=='m1'&p.value<0.01),n=100)
```

### Selected Model Terms

```
## # A tibble: 39 x 6
##    term                                            estimate std.error statistic  p.value MOD
##    <chr>                                              <dbl>     <dbl>     <dbl>    <dbl> <chr>
##  1 factor(HV007)2024                                -0.0763    0.0229     -3.34 0.000852 m1
##  2 factor(HV023)Huancavelica                         0.339     0.106       3.20 0.00139  m1
##  3 factor(HV007)2018:factor(HV023)Ayacucho           0.164     0.0627      2.62 0.00880  m1
##  4 factor(HV007)2013:factor(HV023)Cusco              0.236     0.0823      2.86 0.00421  m1
##  5 factor(HV007)2015:factor(HV023)Cusco              0.221     0.0614      3.59 0.000326 m1
##  6 factor(HV007)2016:factor(HV023)Cusco              0.191     0.0640      2.99 0.00279  m1
##  7 factor(HV007)2017:factor(HV023)Cusco              0.244     0.0634      3.85 0.000120 m1
##  8 factor(HV007)2018:factor(HV023)Cusco              0.252     0.0656      3.85 0.000120 m1
##  9 factor(HV007)2019:factor(HV023)Cusco              0.256     0.0671      3.81 0.000140 m1
## 10 factor(HV007)2020:factor(HV023)Cusco              0.196     0.0663      2.96 0.00306  m1
## 11 factor(HV007)2021:factor(HV023)Cusco              0.248     0.0656      3.78 0.000159 m1
## 12 factor(HV007)2022:factor(HV023)Cusco              0.203     0.0636      3.19 0.00142  m1
## 13 factor(HV007)2023:factor(HV023)Cusco              0.247     0.0684      3.61 0.000308 m1
## 14 factor(HV007)2021:factor(HV023) Puno              0.243     0.0815      2.98 0.00285  m1
## 15 factor(HV023)Apurimac:BURB2                       0.257     0.0759      3.39 0.000700 m1
## 16 factor(HV023)Arequipa:BURB2                       0.188     0.0617      3.04 0.00235  m1
## 17 factor(HV023)Ayacucho:BURB2                       0.189     0.0662      2.86 0.00423  m1
## 18 factor(HV023)Cajamarca:BURB2                      0.205     0.0720      2.85 0.00433  m1
## 19 factor(HV023)Cusco:BURB2                          0.271     0.0714      3.79 0.000149 m1
## 20 factor(HV023) Huanuco:BURB2                       0.354     0.109       3.24 0.00120  m1
## 21 factor(HV023) Ica:BURB2                           0.200     0.0770      2.60 0.00933  m1
## 22 factor(HV023) Madre de Dios:BURB2                 0.167     0.0630      2.65 0.00809  m1
## 23 factor(HV023) Puno:BURB2                          0.246     0.0876      2.81 0.00497  m1
## 24 factor(HV007)2015:factor(HV023)Cajamarca:BURB2   -0.233     0.0832     -2.79 0.00520  m1
## 25 factor(HV007)2018:factor(HV023)Cajamarca:BURB2   -0.237     0.0888     -2.67 0.00765  m1
## 26 factor(HV007)2015:factor(HV023)Cusco:BURB2       -0.236     0.0875     -2.70 0.00700  m1
## 27 factor(HV007)2017:factor(HV023)Cusco:BURB2       -0.265     0.0939     -2.82 0.00478  m1
## 28 factor(HV007)2018:factor(HV023)Cusco:BURB2       -0.285     0.0919     -3.10 0.00194  m1
## 29 factor(HV007)2019:factor(HV023)Cusco:BURB2       -0.306     0.0904     -3.39 0.000702 m1
## 30 factor(HV007)2023:factor(HV023)Cusco:BURB2       -0.284     0.101      -2.81 0.00493  m1
## 31 factor(HV007)2017:factor(HV023) Ica:BURB2        -0.272     0.0898     -3.03 0.00243  m1
## 32 factor(HV007)2018:factor(HV023) Ica:BURB2        -0.263     0.0958     -2.75 0.00597  m1
## 33 factor(HV007)2018:factor(HV023) Madre de Dios:BURB2 -0.259  0.0833     -3.11 0.00190  m1
## 34 factor(HV007)2023:factor(HV023) Madre de Dios:BURB2 -0.209  0.0795     -2.63 0.00863  m1
## 35 factor(HV007)2023:factor(HV023) Piura:BURB2      -0.208     0.0783     -2.66 0.00776  m1
## 36 factor(HV007)2015:factor(HV023) Puno:BURB2       -0.296     0.106      -2.78 0.00537  m1
## 37 factor(HV007)2019:factor(HV023) Puno:BURB2       -0.285     0.107      -2.66 0.00778  m1
## 38 factor(HV007)2021:factor(HV023) Puno:BURB2       -0.295     0.103      -2.87 0.00407  m1
## 39 factor(HV007)2023:factor(HV023) Puno:BURB2       -0.315     0.112      -2.81 0.00499  m1
```

```
L=GDX8(mm=m3,NDSIM=30,FNSAM=0.05)
```

DNOR gaussian

**Model Diagnostics (quantile residuals)**

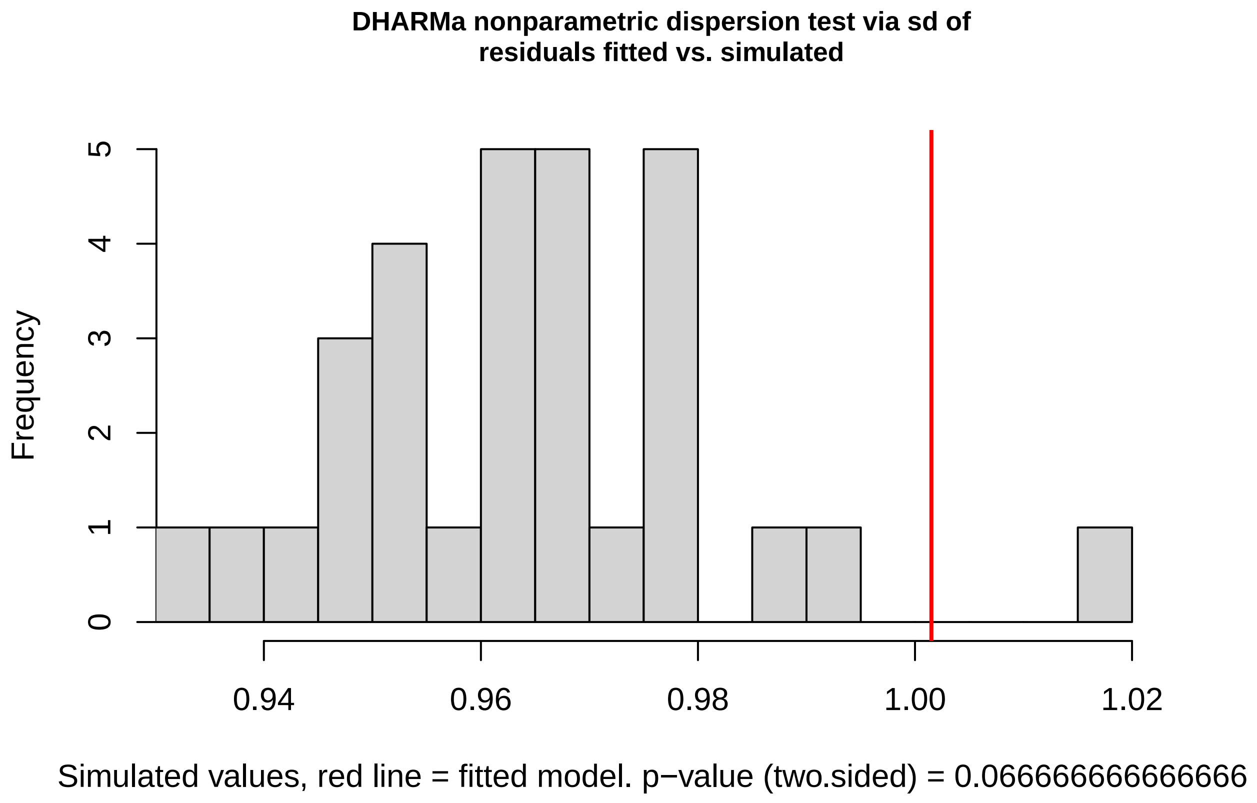

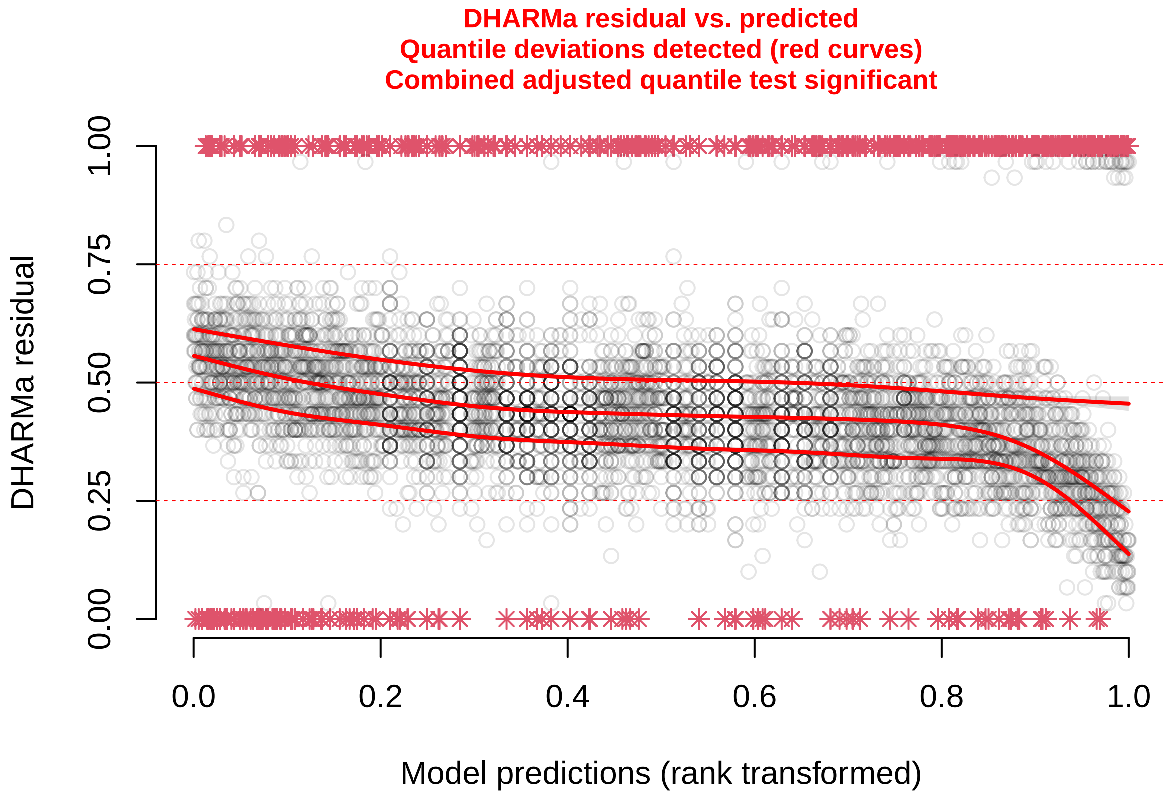
DHARMa residual vs. predicted
Quantile deviations detected (red curves)
Combined adjusted quantile test significant
DHARMa residual
0.00
0.25
0.50
0.75
1.00
0.0
0.2
0.4
0.6
0.8
1.0
Model predictions (rank transformed)

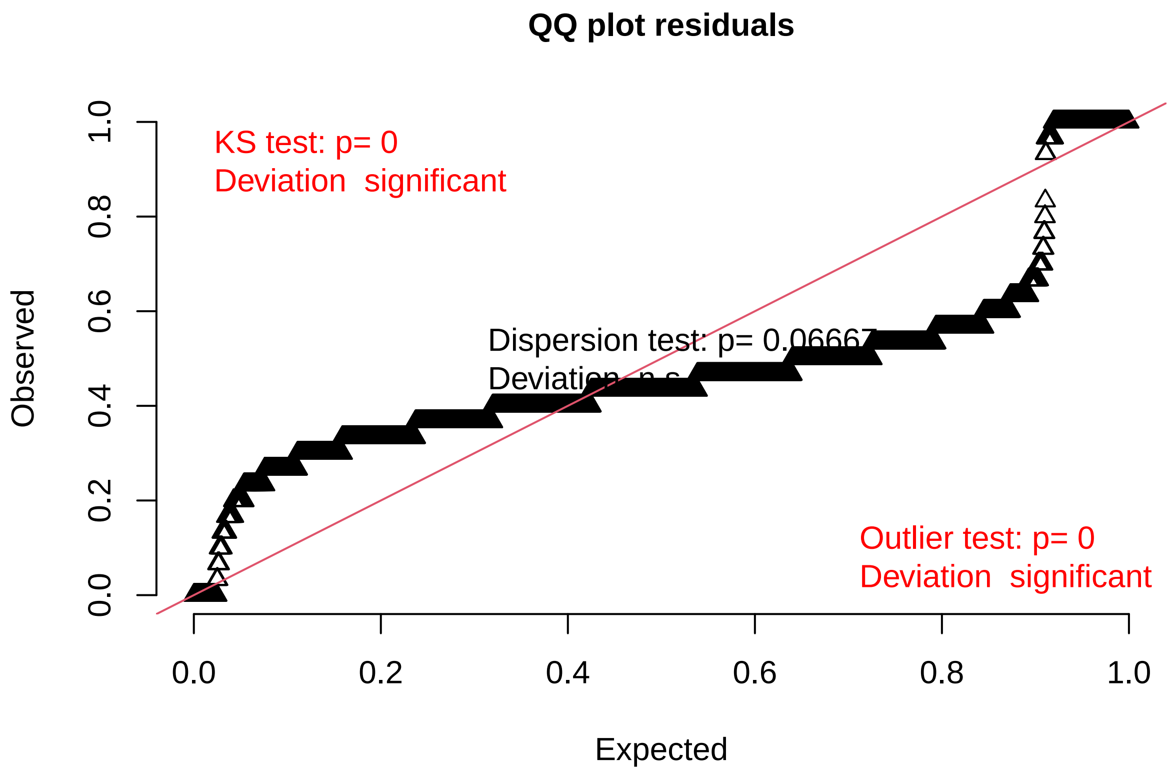
QQ plot residuals
KS test: p= 0
Deviation significant
Dispersion test: p= 0.06667
Deviation n.s.
Outlier test: p= 0
Deviation significant
Observed
Expected
0.0
0.2
0.4
0.6
0.8
1.0

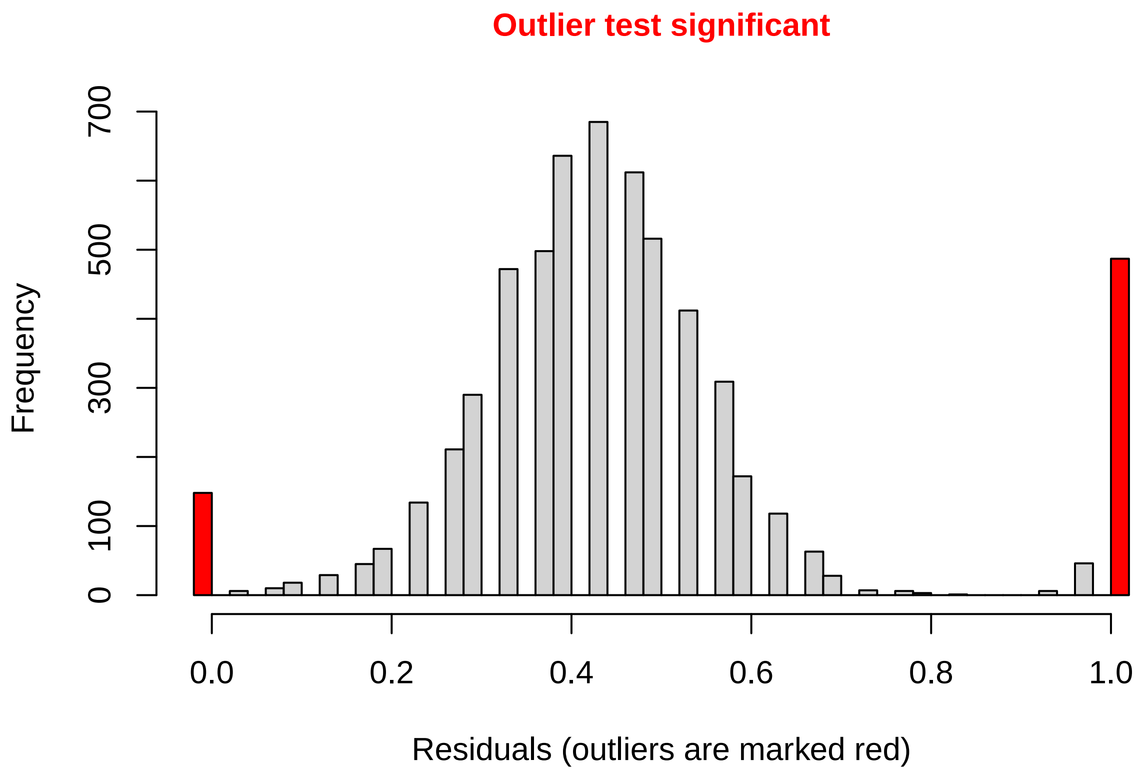


```r
print(L,n=nrow(L))
```

```
## # A tibble: 8 x 3
##   IPAR       XVAL DSC
##   <chr>     <dbl> <chr>
## 1 PDISP1 6.67e- 2 p, Nonpar Dispersion test
## 2 PQGAM2 0        p, Combined Adjusted Quantile test
## 3 POSKS3 0        p, Asymptotic One-Sample Kolmogorov-Smirnov test
## 4 POUTB4 1.80e-32 p, Outlier Exact Binomial test
## 5 PEXPE4 6.45e- 2 Expected Proportion of Outliers
## 6 POBSE4 1.05e- 1 Observed Proportion of Outliers
## 7 PLLOW4 9.76e- 2 Lower CL Proportion of Outliers
## 8 PLUPP4 1.13e- 1 Upper CL Proportion of Outliers
```

### Model: DCAM~ m2 Diagnostic Change Proportion vs covars

```r
print(m1$call)
```

### Model Summary

```
## svyglm(formula = DNOR ~ factor(HV007) * factor(HV023) * BURB2,
##     design = x, family = gaussian)
```

```r
LALT$RMRES|>filter(MOD=='m2')
```

```
## # A tibble: 1 x 9
##   null.deviance df.null    AIC    BIC deviance df.residual   nobs MOD   MCFAR2
##           <dbl>   <int>  <dbl>  <dbl>    <dbl>       <dbl>  <int> <chr>  <dbl>
## 1        83576.  120710 82742. 89665.   81042.       33386 120711 m2    0.0303
```

```r
print(LALT$RMFIN$M2$AOV)
```

### Analysis of Deviance

```
## Anova table:  (Rao-Scott LRT)
## svyglm(formula = DCAM ~ factor(HV007), design = x, family = binomial)
##                                    stats    DEff     df   ddf        p
## factor(HV007)                       72.7    1.98  15.00 34107   0.0051 **
## factor(HV023)                     1430.0    1.31  24.00 34083  < 2e-16 ***
## BURB2                               32.6    1.60   1.00 34082  6.9e-06 ***
## factor(HV007):factor(HV023)        480.6    1.24 337.00 33745   0.0842 .
## factor(HV007):BURB2                 21.9    1.51  14.00 33731   0.4050
## factor(HV023):BURB2                 98.4    1.17  23.00 33708  1.8e-06 ***
## factor(HV007):factor(HV023):BURB2  397.9    1.04 322.00 33386   0.0469 *
## ---
## Signif. codes:  0 '***' 0.001 '**' 0.01 '*' 0.05 '.' 0.1 ' ' 1
```

```r
print(LALT$RMTER|>filter(MOD=='m2'&p.value<0.01),n=100)
```

**Selected Model Terms**

```
## # A tibble: 28 x 6
##    term                                              estimate std.error statistic  p.value MOD
##    <chr>                                                <dbl>     <dbl>     <dbl>    <dbl> <chr>
##  1 (Intercept)                                          -2.64     0.774     -3.41 6.58e- 4 m2
##  2 factor(HV007)2024                                   -11.7      1.02     -11.6  7.21e-31 m2
##  3 factor(HV023)Huancavelica                             2.26     0.868      2.60 9.29e- 3 m2
##  4 factor(HV007)2011:factor(HV023)Cajamarca            -11.2      1.23      -9.15 6.21e-20 m2
##  5 factor(HV023)Arequipa:BURB2                         -12.5      0.953    -13.2  1.94e-39 m2
##  6 factor(HV007)2010:factor(HV023)Arequipa:BURB2        12.3      1.32       9.37 8.04e-21 m2
##  7 factor(HV007)2011:factor(HV023)Arequipa:BURB2        13.4      1.45       9.23 2.95e-20 m2
##  8 factor(HV007)2012:factor(HV023)Arequipa:BURB2        12.0      1.51       7.95 1.94e-15 m2
##  9 factor(HV007)2013:factor(HV023)Arequipa:BURB2        12.6      1.38       9.15 6.05e-20 m2
## 10 factor(HV007)2014:factor(HV023)Arequipa:BURB2        14.4      1.28      11.3  2.03e-29 m2
## 11 factor(HV007)2015:factor(HV023)Arequipa:BURB2        13.1      1.10      11.9  1.94e-32 m2
## 12 factor(HV007)2016:factor(HV023)Arequipa:BURB2        13.6      1.15      11.8  6.01e-32 m2
## 13 factor(HV007)2017:factor(HV023)Arequipa:BURB2        12.9      1.08      11.9  1.15e-32 m2
## 14 factor(HV007)2018:factor(HV023)Arequipa:BURB2        13.1      1.19      11.0  3.99e-28 m2
## 15 factor(HV007)2019:factor(HV023)Arequipa:BURB2        12.0      1.20      10.0  1.28e-23 m2
## 16 factor(HV007)2020:factor(HV023)Arequipa:BURB2        12.9      1.26      10.2  2.13e-24 m2
## 17 factor(HV007)2021:factor(HV023)Arequipa:BURB2        13.6      1.10      12.4  1.70e-35 m2
## 18 factor(HV007)2022:factor(HV023)Arequipa:BURB2        11.0      1.24       8.85 9.47e-19 m2
## 19 factor(HV007)2023:factor(HV023)Arequipa:BURB2        12.6      1.19      10.6  2.73e-26 m2
## 20 factor(HV007)2011:factor(HV023)Cajamarca:BURB2       12.0      1.39       8.60 8.41e-18 m2
## 21 factor(HV007)2014:factor(HV023)Huancavelica:BURB2     4.01     1.38       2.91 3.67e- 3 m2
## 22 factor(HV007)2014:factor(HV023) Junin:BURB2           3.43     1.22       2.81 5.00e- 3 m2
## 23 factor(HV007)2014:factor(HV023) Lambayeque:BURB2      3.90     1.45       2.68 7.35e- 3 m2
## 24 factor(HV007)2011:factor(HV023) Lima:BURB2           -9.80     1.46      -6.69 2.19e-11 m2
## 25 factor(HV007)2014:factor(HV023) Tacna:BURB2           4.49     1.67       2.70 6.99e- 3 m2
## 26 factor(HV007)2020:factor(HV023) Tacna:BURB2          -9.24     1.69      -5.45 4.94e- 8 m2
## 27 factor(HV007)2012:factor(HV023) Tumbes:BURB2        -10.8      1.39      -7.82 5.42e-15 m2
## 28 factor(HV007)2018:factor(HV023) Tumbes:BURB2        -10.7      1.21      -8.85 8.85e-19 m2
```

```
L=GDX8(mm=m4,NDSIM=30,FNSAM=0.05)
```

DCAM binomial

**Model Diagnostics (quantile residuals)**

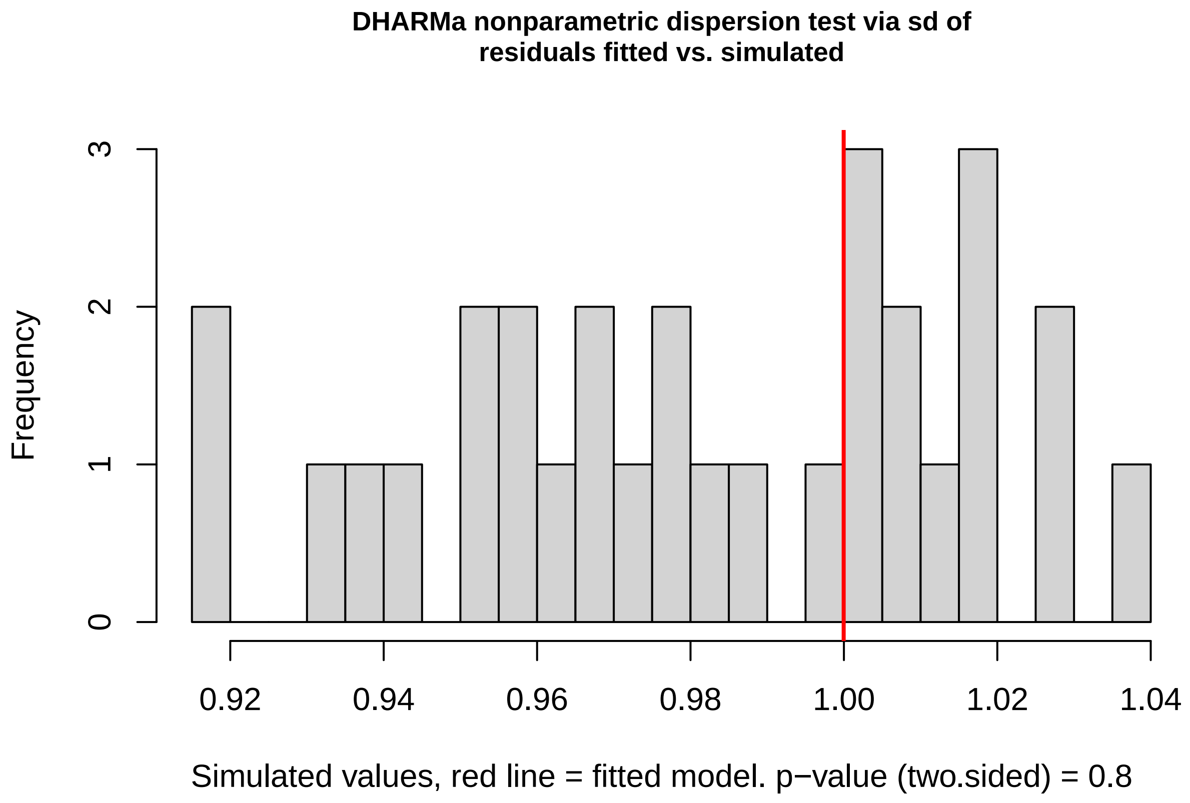

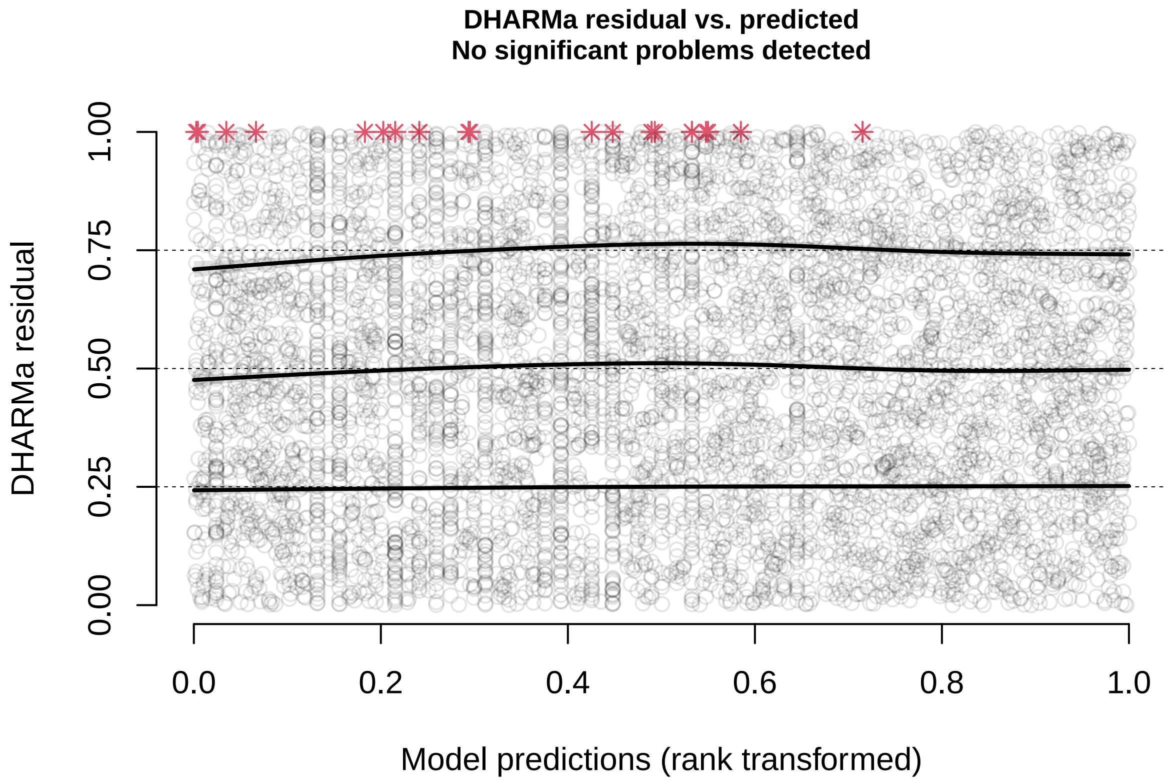
DHARMa residual vs. predicted
No significant problems detected
DHARMa residual
0.00
0.25
0.50
0.75
1.00
0.0
0.2
0.4
0.6
0.8
1.0
Model predictions (rank transformed)

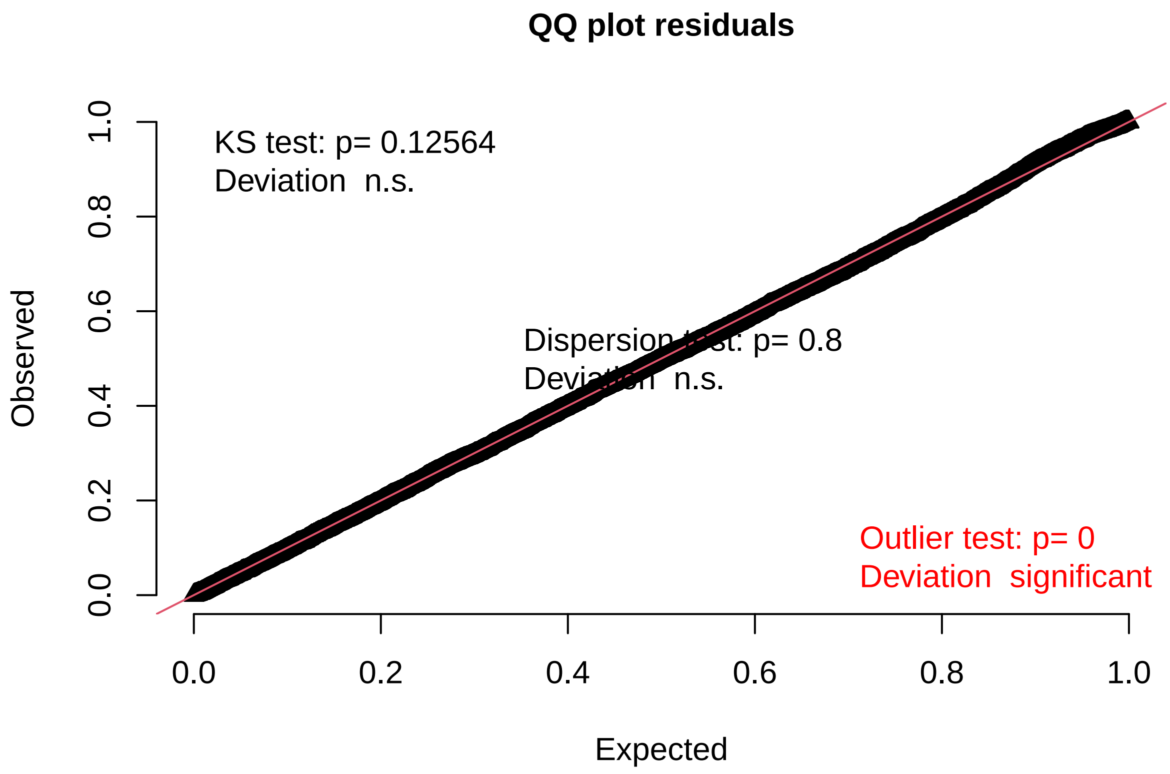
QQ plot residuals
KS test: p= 0.12564
Deviation n.s.
Dispersion test: p= 0.8
Deviation n.s.
Outlier test: p= 0
Deviation significant
Observed
0.0
0.2
0.4
0.6
0.8
1.0
Expected

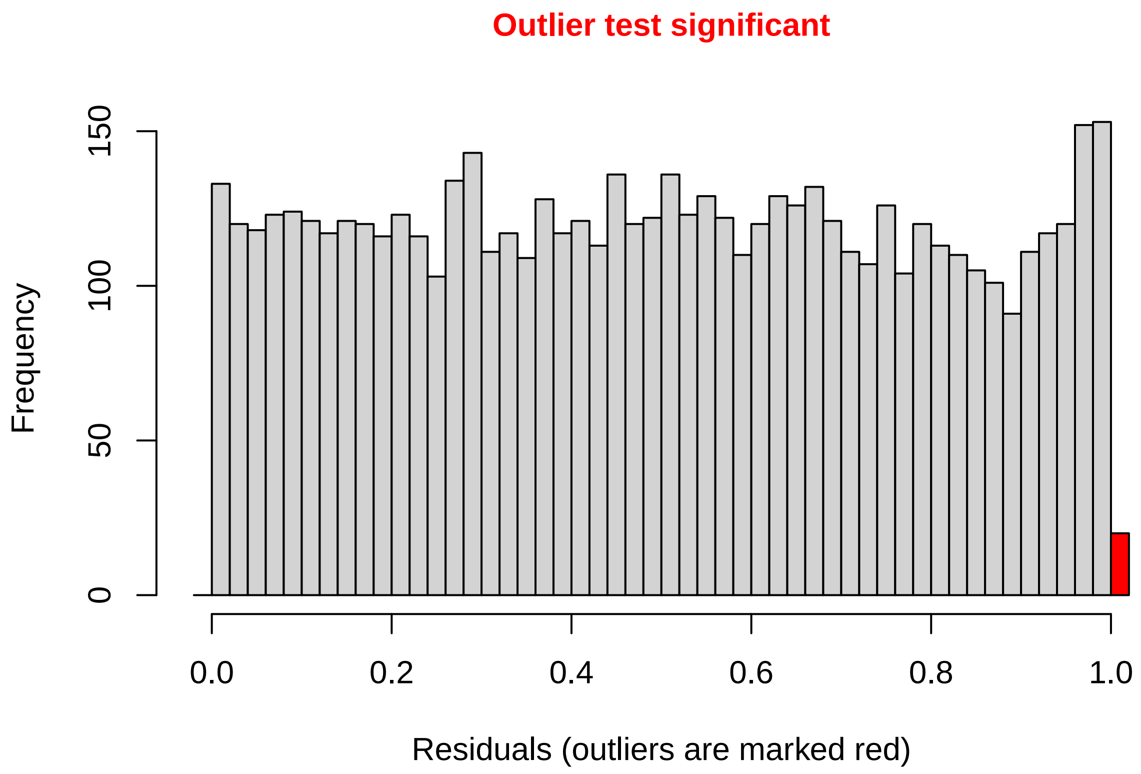


```
print(L,n=nrow(L))
```

```
## # A tibble: 8 x 3
##   IPAR         XVAL DSC
##   <chr>       <dbl> <chr>
## 1 PDISP1 0.8        p, Nonpar Dispersion test
## 2 PQGAM2 0.365      p, Combined Adjusted Quantile test
## 3 POSKS3 0.126      p, Asymptotic One-Sample Kolmogorov-Smirnov test
## 4 POUTB4 0.00000267 p, Outlier Exact Binomial test
## 5 PEXPE4 0.0645     Expected Proportion of Outliers
## 6 POBSE4 0.0799     Observed Proportion of Outliers
## 7 PLLOW4 0.0731     Lower CL Proportion of Outliers
## 8 PLUPP4 0.0870     Upper CL Proportion of Outliers
```

## Comentarios

El propósito básico de esta nota es presentar los resultados detallados del modelamiento con algunos diagnósticos exploratorios para chequear la validez de los modelos y las conclusiones basadas en ellos.

La estimación básica de todas las medias está sustentada por el teorema central del límite que garantiza la confiabilidad de los intervalos de confianza. Además, el modelamiento binomial de la variable dicótoma, siendo una regresión logística, también está sustentado.

Los retos en este caso surgirán del gran número de casos, el gran número de términos, el diseño de muestra compleja y la no normalidad de la variable tricótoma.

El núcleo de cualquier diagnóstico de modelos es la exploración de la distribución de residuos. Esta distribución puede ser suavizada usando residuos cuantílicos, la técnica aplicada aquí.

Debido al tamaño, no pudimos usar el paquete svydiags, y aún para el paquete DHARMa tuvimos que submuestrear los modelos mas grandes.

Con algo de ironía, la muestra es suficientemente grande para que varias pruebas diagnósticas tengan significancia. Pero nuestra impresión es que las desviaciones no son tan grandes que cuestionen las conclusiones de los artículos (el órden de magnitud de las diferencias y su variación sobre extremos importantes). No intentamos formular afirmaciones de magnitud para escenarios específicos. Cada uno en particular tendría que ser evaluado separadamente por los interesados.

Como esperabamos, la cantidad de variabilidad 'explicada', reflejada en el R2 de McFadden, es baja. Estos no son modelos explicativos, solo descriptivos señalando la existencia de gran variabilidad.

Debemos reiter que los valores no son particularmente informativos en este escenario. Debería darse atención a los intervalos de confianza.

## Referencias


1. Dunn PK, Smyth GK. Randomized Quantile Residuals. Journal of Computational and Graphical Statistics. 1996;5(3):236–44. doi:10.2307/1390802
2. Harrell FE. Regression modeling strategies: with applications to linear models, logistic and ordinal regression, and survival analysis. Second edition. New York, NY, USA: Springer; 2015. 582 p. (statistics).
3. Hartig F. DHARMa | Diagnostics for HierArchical Regression Models [Internet]. 2022 [citado el 28 de noviembre de 2021]. Disponible en: http://florianhartig.github.io/DHARMa/
4. Lohr SL. Sampling: design and analysis. 2nd ed. Boston, Mass: Brooks/Cole; 2010. 596 p.
5. Lumley T. Complex surveys: a guide to analysis using R. Hoboken, N.J: John Wiley; 2010. 276 p. (Wiley series in survey methodology).
6. Lumley T. Survey: analysis of complex survey samples [Internet]. Vienna, Austria: R Foundation for Statistical Computing; 2012 [citado el 8 de febrero de 2013]. Disponible en: http://CRAN.R-project.org/package=survey
7. McCullagh P, Nelder JA. Generalized Linear Models. 2a ed. Boca Raton, FL: Chapman and Hall/CRC; 1989. 532 p.
8. R Core Team. R: A language and environment for statistical computing [Internet]. Vienna, Austria: R Foundation for Statistical Computing; 2024 [citado el 10 de enero de 2025]. Disponible en: http://www.R-project.org
9. Wickham H, Grolemund G. R for Data Science: Import, Tidy, Transform, Visualize, and Model Data [Internet]. 1a ed. Boston, MA: O'Reilly Media; 2017. 522 p. Disponible en: https://r4ds.had.co.nz/

## Programa R

## P2025021620.R MCNut24.R 2025-Feb-17 vipermcs@gmail.com et al 2021-Mar-08

```
# ------------------------------------------------------------------------------- #
# Basic Packages
require(tidyverse)
require(survey)
require(survival)
require(future)
require(ggpattern)
windows(width=6,height=6,record=TRUE)
options(digits=5,width=240)
options(survey.lonely.psu="adjust")
options(dplyr.summarise.inform=FALSE,dplyr.show_progress=FALSE)
# ------------------------------------------------------------------------------- #
# Parameters
PCRIT = 0.05
ZCRIT = qnorm(1-PCRIT/2)
ALPHA = PCRIT
DWORK = "Z:\" # carpeta de trabajo
DTABS = "X:\Tabs\" # carpeta con tablas, puede ser DTABS="Z:\"
setwd(DWORK)
RRSEED=prod(as.numeric(unlist(strsplit("9 92 91","\s+")))) # https://www.random.org/cgi-bin/rand
byte?nbytes=4&format=d 2024-Mar-29 15:50 52
NCORES=parallel::detectCores()
# ------------------------------------------------------------------------------- #
# Documentation
# Software for the paper
# "New WHO guideline on the definition of anemia:
# implications for 6-35 months old children in Peru 2009-2023"
# Miguel Campos, Luis Cordero, Enrique Velásquez, Nelly Baiocchi,
# Marianella Miranda, María Inés Sánchez-Griñán, Walter Valdivia.
# This code has three parts:
# I Consolidado ENDES
# Provided here only as documentation, it will not necessarily execute.
# Generates N44, a consolidated data frame of ENDES 2009-2023 children.
# It is used for several purposes in our group.
# It depends on downloaded ENDES ZIPs in folders for each year,
# and a table listing files and years for ENDES (in PETabs.rda).
# II Preparación de Datos
```

```
# Provided here only as documentation, it will not necessarily execute.
# Generates d, a data frame (tibble), summarized subset of N44.
# It is used only for analysis in this paper.
# III Artículo
# It can be executed together with the d data frame.
# It requires R and the tidyverse & survey packages.
# It has two sections:
# - Generation of Weighed Estimates (it can take a while)
# - Production of Graphs
# ---------------------------------------------------------------------------------- #
# I Consolidado ENDES
if(FALSE){ # endes
# H00,H01,H03,H06 son UNIONs de los RECH0, RECH1, RECH3 y RECH6 anuales
# TPPBE17 son las proyecciones del Boletín 17 del INEI
h00=
H00%>%
mutate(
    HHID=as.character(HHID),
    W1R=HV005/1E6,
    W1RX=if_else(VV007==2015,HV005X/1E6,NA),
    SID=HV022*100+HV023
)%>%
mutate(
    VV006=HV008-12*(HV007-1900)
)%>%
select(one_of(c(
    "HHID","W1R","W1RX","SID",
    "HV001","HV022","HV023","HV025","HV026",
    "HV007","VV006","VV007","HV015","HV016","HV040"
)))%>%   # ,"HV024","HV015","HV042"
left_join(y=H03,by=(c("VV007","HHID")))
N06=
H06%>%
left_join(h00,by=c("VV007","HHID"))%>%
left_join(H01%>%rename(HC0=HVIDX),by=c("VV007","HHID","HC0"))
N06=N06%>%filter(!((VV007%in%c(2004:2008))&(HV007!=VV007)))
```

```
N06=
N06%>%
mutate(
    FEnt=as.Date(paste(VV006,"/",HV016,"/",HV007,sep=""),"%m/%d/%Y"),    # está en FENT
    FNac=as.Date(paste(HC30,"/",HC16,"/",HC31,sep=""),"%m/%d/%Y")
)%>%
mutate(
    EdadM=ifelse(HV007%in%c(2018),HC1,as.numeric(FEnt-FNac)/30.4375),
    IDTE=ifelse(is.na(HC70),NA,ifelse(HC70>6000,NA,ifelse(HC70<(-200),1,0))),
    IDPT=ifelse(is.na(HC72),NA,ifelse(HC72>6000,NA,ifelse(HC72<(-200),1,0))),
    ISPO=ifelse(is.na(HC70),NA,ifelse(HC70>6000,NA,ifelse(HC70>200,1,0))),
    HC56=ifelse(HC56<20|HC56>400,NA,HC56),
    IANE=ifelse(is.na(HC56),NA,ifelse(HC56<110,1,0)),
    GA12=12*as.integer(HC1/12)+6
)%>%
mutate(
    GEDA=as.integer(HC1/ifelse(HC1<6,3,ifelse(HC1<12,6,12)))*ifelse(HC1<6,3,ifelse(HC1<12,6,12))+ifelse(HC1<6,3,ifelse(HC1<12,6,
    FENT=ISOdate(HV007,VV006,HV016)
)
x=
TPPBE17%>%
filter(EDADS%in%0:2)%>%
select(EDADS,A2000,A2003:A2007,A2008,A2009:A2023)
y=(x[1,-1]/2+x[2,-1]+x[3,-1])
z=N06%>%group_by(VV007)%>%summarize(TW1R=sum(W1R,na.rm=TRUE))
u=unlist(sapply(1:nrow(N06),function(i){(N06W1R[i]/zTW1R[zVV007 == N06VV007[i]])*y[1,paste0("A",as.character(N06$
N06$W1RP=u
} # endes
# ---------------------------------------------------------------------------------- #
# II Preparación de Datos
if(FALSE){ # load
Y=N06
d=
Y%>%
filter(
```

```
    VV007%in%c(2009:2023)&
    !is.na(HC1)&HC1>=6&HC1<36&!is.na(HC53)&
    HV103==1&HV015==1&HC55==0
)%>%
mutate(
    ADH=((-0.032*(HV040*0.0033)+0.022*(HV040*0.0033)^2)*10),     # ecuación ENDES
    AAW=((-0.032*(HV040*0.0032808)+0.022*(HV040*0.0032808)^2)*10),   # CDC 1989, WHO 2011
    A24=(0.0056384*HV040)+(0.0000003*HV040^2),           # WHO 2024, continuous
    AS4=c(0,4,8,11,14,18,21,25,29,33)[(ifelse(HV040>=5000,4999,HV040)%/%500)+1],             # WHO 2024, staircase
    SID2=HV023*10+HV025
)%>%
mutate(
    IANE3=ifelse((HC53-ifelse(HV040<1000&HV007>=2022,0,ADH))<110,1,0),
    IANE4=ifelse((HC53-A24)<ifelse(HC1<24,105,110),1,0),
    IANE6=ifelse((HC53-AS4)<ifelse(HC1<24,105,110),1,0),
    FEDA3=factor(x=12*as.integer(HC1/12),labels=c("6-11m","12-23m","24-35m")),
    FALT3=factor(x=ifelse(HV040<1000,0,ifelse(HV040<3000,2000,3500)),labels=c("0-0.9km","1-2.9km","3+km")),
    BURB2=ifelse(as.character(haven::as_factor(HV025))=="Urbano",0,1)
)%>%
mutate(
    DNOR=IANE3-IANE4,
    DCAL=IANE6-IANE4
)%>%
mutate(
    DNAS=if_else(DNOR==(-1),1,0),
    DSAN=if_else(DNOR==1,1,0),
    DCAM=if_else(DNOR==0,0,1)
)%>%
mutate(W1RA=HV005A/1000000)%>%
mutate(across(c(HV023,HV025),~haven::as_factor(.)))%>%
select(
    HV007,VV007,VV006,HC1,HC27,IANE,HC56,HC53,
    ADH,AAW,A24,AS4,IANE3,IANE4,IANE6,
```

```
    DNOR,DCAL,DNAS,DSAN,DCAM,FEDA3,FALT3,BURB2,
    HV001,HV022,HV023,HV025,HV026,HV040,SHREGION,
    HHID,W1RP,W1R,W1RX,W1RA,SID,SID2
)%>%
filter((HC53-ADH)>10&(HC53-ADH)<300)%>%
labelled::set_variable_labels(
    HV007   ="año de encuesta (real)",
    VV007   ="año de encuesta (nominal)",
    VV006   ="mes de encuesta",
    HC1     ="edad, meses cumplidos",
    HC27    ="sexo",
    IANE    ="anemia (DHS)",
    HC56    ="hb corregida (DHS)",
    HC53    ="hb sin corregir",
    AAW     ="ajuste WHO 2011, ecuación ENDES",
    ADH     ="ajuste WHO 2011, pies 1959",
    A24     ="ajuste WHO 2024, continuo",
    AS4     ="ajuste WHO 2024, escalera",
    IANE3   ="anemia (WHO 2001)",
    IANE4   ="anemia (WHO 2024, continua)",
    IANE6   ="anemia (WHO 2024, escalera)",
    DNOR    ="diferencia estado de anemia 2001-2024",
    DCAL    ="diferencia estado de anemia tabla-fórmula",
    DNAS    ="cambió de no anémico 2001 a anémico 2024",
    DSAN    ="cambió de anémico 2001 a no anémico 2024",
    DCAM    ="cambió diagnóstico 2001 vs 2024",
    FEDA3   ="edad (años)",
    FALT3   ="altitud (3)",
    HV040   ="altitud m snm",
    HHID    ="id caso",
    W1R     ="ponderación original HV005",
    W1RX    ="ponderación original HV005X 2015",
```

```
    W1RA    ="ponderación original HV005A 2020",
    W1RP    ="ponderación, reescalada",
    SID     ="estrato: HV022+región",
    SID2    ="estrato: región+ámbito"
)
# reponderación, homogeneización
y=
N06%>%group_by(VV007)%>%summarize(NN=sum(W1RP))%>%
mutate(NX=NN*2.5/5)%>%
left_join(y=d%>%group_by(VV007)%>%summarize(NW=sum(W1RP)),by="VV007")
d=
d%>%
left_join(y=y,by="VV007")%>%
mutate(W1RF=case_when(VV007==2015~W1RX,VV007==2020~W1RA,TRUE~W1R)*NX/NW)%>%
select(-NN,-NW)
# diseño de la muestra
s=survey::svydesign(ids=HV001+HHID,weights=W1RF,strata=~VV007+HV022,data=d,nest=TRUE)
# parche descriptivo
y=
Y%>%
mutate(HV023=haven::as_factor(HV023))%>%
group_by(HV023)%>%
summarize(
    NN06=n(),
    NYA9=sum(if_else(VV007>2008&(is.na(HC1)|(HC1>=6&HC1<36)),1,0)),
)%>%
left_join(y=d%>%group_by(HV023)%>%summarize(NDIN=n()),by="HV023")
z=
Y%>%
group_by(VV007)%>%
summarize(
    NN06=n(),
    NYA9=sum(if_else(VV007>2008&(is.na(HC1)|(HC1>=6&HC1<36)),1,0))
)%>%
left_join(y=d%>%group_by(VV007)%>%summarize(NDIN=n()),by="VV007")
```

```
LALT$DAT=d
LALT$SVY=s
} # load
# ---------------------------------------------------------------------------------- #
# III Artículo
load(file="X2024031817.rda")
# Generación de Estimaciones
if(TRUE){ # estp
# RY0 resumen antes de la exclusión
if(TRUE){
LALT$RY0=y
z=
z%>%
mutate(
    CIC=
        case_when(
            VV007==2000~"00",
            VV007>=2004&VV007<=2008~"04-08",
            VV007>=2009&VV007<=2023~paste0(
                sprintf("%02d",(3*(VV007-2009)%/%3)+9),
                "-",
                sprintf("%02d",(3*(VV007-2009)%/%3)+9+2)
                ),
            TRUE~NA
        ),
    MMU=
        case_when(
            VV007>=2009&VV007<=2014~"CNPV 2007",
            VV007>=2015&VV007<=2020~"CNPV+SISFOH",
            VV007>=2021&VV007<=2023~"CNPV 2017",
            TRUE~NA
        ),
    DMU=
        case_when(
            VV007>=2009&VV007<=2014~"Estratificado Bietápico",
```

```
            VV007>=2015&VV007<=2023~"Equilibrado",
            TRUE~NA
        ),
    DNO=
        case_when(
            VV007>=2012&VV007<=2014~"+Hogares Secundarios",
            VV007==2020~"Pandemia, Parte Virtual",
            TRUE~NA
        )
)
LALT$RZ0=z
}
# R0 estimaciones nacionales
if(TRUE){
print(date())
m=survey::svyby(formula=IANE3,by=VV007,design=s,FUN=survey::svymean,na.rm=TRUE)
z=survey::svyby(formula=IANE4,by=VV007,design=s,FUN=survey::svymean,na.rm=TRUE)
v=survey::svyby(formula=~DNOR, by=~VV007,design=s,FUN=survey::svymean,na.rm=TRUE)
r=
as_tibble(m)%>%
rename(se3=se)%>%
left_join(y=as_tibble(z)%>%rename(se4=se),by="VV007")%>%
left_join(y=as_tibble(v)%>%rename(sed=se),by="VV007")%>%
mutate(
    l3=IANE3-ZCRIT*se3,
    u3=IANE3+ZCRIT*se3,
    l4=IANE4-ZCRIT*se4,
    u4=IANE4+ZCRIT*se4,
    ld=DNOR-ZCRIT*sed,
    ud=DNOR+ZCRIT*sed
)
y=d%>%group_by(VV007)%>%summarize(f=n())
r=r%>%left_join(y=y,by="VV007")
LALT$R0 =r # estimaciones nacionales
}
```

```
# R4B estimaciones en regiones
if(TRUE){
r=
srvyr::as_survey_design(s)%>%
group_by(HV023)%>%
summarize(
    PR1=srvyr::survey_mean(x=IANE, na.rm=TRUE,vartype=c("ci")),
    PR3=srvyr::survey_mean(x=IANE3,na.rm=TRUE,vartype=c("ci")),
    PR4=srvyr::survey_mean(x=IANE4,na.rm=TRUE,vartype=c("ci")),
    PR6=srvyr::survey_mean(x=IANE6,na.rm=TRUE,vartype=c("ci")),
    DNOR=srvyr::survey_mean(x=DNOR,na.rm=TRUE,vartype=c("ci")),
    DCAL=srvyr::survey_mean(x=DCAL,na.rm=TRUE,vartype=c("ci")),
    PNAS=srvyr::survey_mean(x=DNAS,na.rm=TRUE,vartype=c("ci")),
    PSAN=srvyr::survey_mean(x=DSAN,na.rm=TRUE,vartype=c("ci")),
    PCAM=srvyr::survey_mean(x=DCAM,na.rm=TRUE,vartype=c("ci")),
    SWTO=sum(W1RF),
    NNOP=srvyr::unweighted(n())
)
LALT$R4B=r # estimaciones por región
# #.Callao, Lima Metro
}
# R4C estimaciones en regiones, ámbitos y años
if(TRUE){
r=
srvyr::as_survey_design(s)%>%
group_by(HV023,HV025,VV007)%>%
summarize(
    PR3=srvyr::survey_mean(x=IANE3,na.rm=TRUE,vartype=c("ci")),
    PR4=srvyr::survey_mean(x=IANE4,na.rm=TRUE,vartype=c("ci")),
    PR6=srvyr::survey_mean(x=IANE6,na.rm=TRUE,vartype=c("ci")),
    DNOR=srvyr::survey_mean(x=DNOR,na.rm=TRUE,vartype=c("ci")),
    DCAL=srvyr::survey_mean(x=DCAL,na.rm=TRUE,vartype=c("ci")),
    PNAS=srvyr::survey_mean(x=DNAS,na.rm=TRUE,vartype=c("ci")),
    PSAN=srvyr::survey_mean(x=DSAN,na.rm=TRUE,vartype=c("ci")),
    PCAM=srvyr::survey_mean(x=DCAM,na.rm=TRUE,vartype=c("ci")),
```

```
    SWTO=sum(W1RF),
    NNOP=srvyr::unweighted(n())
)
LALT$R4C=r # estimaciones por región-ámbito-año
}
# R8 estimaciones alternativas de varios indicadores
if(TRUE){
# magnitud y dispersión de diferencia en prevalencias
r1=survey::svymean(x=~DNOR,design=s);r2=confint(r1)
r5=survey::svymean(x=~IANE4,design=s);r6=confint(r5)
r7=survey::svymean(x=~DCAM,design=s);r8=confint(r7)
s1=survey::svymean(x=~DCAL,design=s);s2=confint(s1)
z2=survey::svyglm(formula=DNOR~1,family=gaussian,design=s)
z3=survey::svyglm(formula=DCAM~1,family=binomial,design=s)
z4=survey::svyglm(formula=DCAL~1,family=gaussian,design=s)
r=
tribble(
    ~EVAL,~LVAL,~UVAL,~PVAL,~DIFE,~VERS,~INDI,
    as.numeric(r5),r6[1],r6[2],NA,"PAN4","1","Media Nacional Ponderada",
    as.numeric(r1),r2[1],r2[2],summary(z2)$coefficients[1,4],"DNOR","1","Media Nacional Ponderada",
    as.numeric(s1),s2[1],s2[2],summary(z4)$coefficients[1,4],"DCAL","1","Media Nacional Ponderada",
    as.numeric(r7),r8[1],r8[2],summary(z3)$coefficients[1,4],"DCAM","1","Proporción de Falsos +/-, Nacional Ponderada"
)
LALT$R8 =r # resumen de estimaciones de indicadores alternativos
print(date())
}
# R9 estimaciones edad x altura x año (u,r)
if(TRUE){
r=
srvyr::as_survey_design(s)%>%
group_by(FEDA3,FALT3,HV025,VV007)%>%
summarize(
    PR3=srvyr::survey_mean(x=IANE3,na.rm=TRUE,vartype=c("ci")),
    PR4=srvyr::survey_mean(x=IANE4,na.rm=TRUE,vartype=c("ci")),
    NNOP=srvyr::unweighted(n())
)
```

```
LALT$R9 =r # estimaciones por grupos de altitud-edad-ámbito-año
}
# RMFIN modelos de selección final DNOR, DCAM
if(TRUE){
print(date())
y=
LALT$DAT%>%
select(DNOR,DCAM,HV007,HV023,BURB2,VV007,HV022,HV001,HHID,W1RF)%>%
mutate(HV022=haven::as_factor(HV022))%>%
mutate(HV007=factor(HV007))
x=survey::svydesign(ids=HV001+HHID,weights=W1RF,strata=~VV007+HV022,data=y,nest=TRUE)
m1=
survey::svyglm(
    formula=DNOR~factor(HV007)*factor(HV023)*BURB2,
    design=x,family=gaussian
)
m2=
survey::svyglm(
    formula=DCAM~factor(HV007)*factor(HV023)*BURB2,
    design=x,family=binomial
)
m3=
survey::svyglm(
    formula=
        DNOR~
        factor(HV023)+
        factor(HV023):factor(HV007)+
        factor(HV023):BURB2,
    design=x,family=gaussian
)
m4=
survey::svyglm(
    formula=
        DCAM~
        factor(HV007)+factor(HV023)+BURB2+
        factor(HV023):BURB2,
```

```
    design=x,family=binomial
)
m5=survey::svyglm(formula=DNOR~factor(HV007),design=x,family=gaussian)
m6=survey::svyglm(formula=DNOR~factor(HV023),design=x,family=gaussian)
m7=survey::svyglm(formula=DCAM~factor(HV023),design=x,family=binomial)
m8=survey::svyglm(formula=DNOR~1,design=x,family=gaussian)
m9=survey::svyglm(formula=DCAM~1,design=x,family=binomial)
print(date())
LALT$RMTER=
bind_rows(
    broom::tidy(x=m1)%>%mutate(MOD="m1"),
    broom::tidy(x=m2)%>%mutate(MOD="m2"),
    broom::tidy(x=m3)%>%mutate(MOD="m3"),
    broom::tidy(x=m4)%>%mutate(MOD="m4"),
    broom::tidy(x=m5)%>%mutate(MOD="m5"),
    broom::tidy(x=m6)%>%mutate(MOD="m6"),
    broom::tidy(x=m7)%>%mutate(MOD="m7"),
    broom::tidy(x=m8)%>%mutate(MOD="m8"),
    broom::tidy(x=m9)%>%mutate(MOD="m9")
)
LALT$RMRES=
bind_rows(
    broom::glance(x=m1)%>%mutate(MOD="m1"),
    broom::glance(x=m2)%>%mutate(MOD="m2"),
    broom::glance(x=m3)%>%mutate(MOD="m3"),
    broom::glance(x=m4)%>%mutate(MOD="m4"),
    broom::glance(x=m5)%>%mutate(MOD="m5"),
    broom::glance(x=m6)%>%mutate(MOD="m6"),
    broom::glance(x=m7)%>%mutate(MOD="m7"),
    broom::glance(x=m9)%>%mutate(MOD="m9")
)%>%
mutate(
        MCFAR2=1-deviance/null.deviance
)
```

```
r1=broom::tidy(x=m1)
r2=broom::tidy(x=m2)
s1=broom::glance(x=m1)
s2=broom::glance(x=m2)
o1=anova(object=m1)
o2=anova(object=m2)
LALT$RMFIN=
list(
    M1=list(AOV=o1,TER=r1,MOD=s1),
    M2=list(AOV=o2,TER=r2,MOD=s2)
)
print(date())
}
} # estp
# Producción de Tablas y Gráficos
if(TRUE){ # outc
# Tabla 1 (ex 2)
l="%4.1f";o="%+4.1f" # l="%5.3f";o="%+5.3f"
a=
LALT$RO%>%
mutate(across(IANE3:ud,~.*100))%>%
mutate(
    PR3R=paste0(sprintf(l,IANE3)," (",sprintf(l,l3)," a ",sprintf(l,u3),")"),
    PR4R=paste0(sprintf(l,IANE4)," (",sprintf(l,l4)," a ",sprintf(l,u4),")"),
    PNOR=paste0(sprintf(o,-DNOR)," (",sprintf(o,-ud)," a ",sprintf(o,-ld),")")
)%>%
mutate(across(PR3R:PNOR,~gsub("\\.",",",.)))%>%
select(VV007,PR3R,PR4R,PNOR)%>%
setNames(c(
    "Año",
    "Prev. Norma 2001",
    "Prev. Norma 2024",
    "Dif. Prev. 2024 - 2001"
))
K="Tabla 1"
J="Estimaciones Anuales"
```

```
S=c(
"Formato: Estimado [Límites Inferior a Superior de 95% de confianza]",
"Prev: Prevalencia",
"Dif: Diferencia entre Prevalencias Norma 2001 menos 2024"
)
g=
a%>%
flextable::flextable()%>%
flextable::add_header_row(values=c(paste0(K,": ",J)),colwidths=ncol(a))%>%
flextable::width(j=1,width=0.5)%>%
flextable::width(j=2:ncol(a),width=2)%>%
flextable::add_footer_lines(S)
LALT$T0102=g
# Tabla 2 (ex 3)
b=
LALT$R4B%>%
mutate(HV023=trimws(as.character(HV023)))%>%
select(HV023,starts_with(c("PR3","PR4","DNOR","PCAM")))
l="%4.1f";o="%+4.1f";p="%5.3f" # l="%5.3f";o="%+5.3f"
a=
b%>%
mutate(across(PR3:DNOR_upp,~.*100))%>%
mutate(
    PR3R=paste0(sprintf(l,PR3)," (",sprintf(l,PR3_low)," a ",sprintf(l,PR3_upp),")"),
    PR4R=paste0(sprintf(l,PR4)," (",sprintf(l,PR4_low)," a ",sprintf(l,PR4_upp),")"),
    PNOR=paste0(sprintf(o,-DNOR)," (",sprintf(o,-DNOR_upp)," a ",sprintf(o,-DNOR_low),")"),
    PCAR=paste0(sprintf(l,PCAM*100)," (",sprintf(l,PCAM_low*100)," a ",sprintf(l,PCAM_upp*100),")")
)%>%
mutate(across(PR3R:PCAR,~gsub("\\.",",",.)))%>%
select(HV023,PR3R,PR4R,PNOR,PCAR)%>%
setNames(c(
    "Región",
    "Prev. Norma 2001",
    "Prev. Norma 2024",
    "Dif. Prev. 2024 - 2001",
```

```
    "Cambian entre 2001 y 2024"
))
K="Tabla 3"
J="Estimaciones Regionales"
S=c(
"Formato: Estimado [Límites Inferior a Superior de 95% de confianza]",
"Prop: Proporción; Prev: Prevalencia",
"Dif: Diferencia entre Prevalencias Norma 2001 menos 2024",
"Cambio, PC: Proporción que cambian Diagnóstico entre Anemia y No Anemia"
)
g=
a%>%
flextable::flextable()%>%
flextable::add_header_row(values=c(paste0(K,": ",J)),colwidths=ncol(a))%>%
flextable::width(j=1,width=1.5)%>%
flextable::width(j=2:5,width=2)%>%
flextable::add_footer_lines(S)
LALT$T0103=g
# Figura 1
y=
bind_rows(
    LALT$R0%>%
    select(VV007,IANE3,se3,l3,u3)%>%
    setNames(c("HV007","IANE","SEM","LANE","UANE"))%>%
    mutate(NVER="2001")
,
    LALT$R0%>%
    select(VV007,IANE4,se4,l4,u4)%>%
    setNames(c("HV007","IANE","SEM","LANE","UANE"))%>%
    mutate(NVER="2024")
)%>%
mutate(
    NVER=factor(x=NVER,levels=c("2024","2001"))
)%>%
```

```
filter(HV007%in%c(2009:2023))
g=
ggplot(data=y)+
geom_ribbon(mapping=aes(x=HV007,ymin=LANE,ymax=UANE,fill=NVER),alpha=0.25)+
geom_line(mapping=aes(x=HV007,y=IANE,color=NVER))+
scale_y_continuous(labels=scales::label_number(decimal.mark=","))+
labs(
    x="año",y="prop. prevalencia anemia",color="norma",fill="norma",
    title="Figura 1 Tendencias de Prevalencia Nacional",
    caption=paste0("INEI/ENDES 6-35m"," n=",nrow(filter(d,HV007!=2024)))
)+
theme_minimal()
LALT$G0101=g
# Figura 2
y=
bind_rows(
    LALT$R9%>%
    select(FEDA3,FALT3,HV025,VV007,PR3,PR3_low,PR3_upp)%>%
    setNames(c("FEDA3","FALT3","HV025","HV007","IANE","LANE","UANE"))%>%
    mutate(NVER="2001")
,
    LALT$R9%>%
    select(FEDA3,FALT3,HV025,VV007,PR4,PR4_low,PR4_upp)%>%
    setNames(c("FEDA3","FALT3","HV025","HV007","IANE","LANE","UANE"))%>%
    mutate(NVER="2024")
)%>%
mutate(
    HV025=
        factor(
            x=as.character(haven::as_factor(HV025)),
            levels=c("Urbano","Rural")
        ),
    NVER=factor(x=NVER,levels=c("2024","2001"))
)%>%
```

```
filter(HV007%in%c(2009:2023))
g=
ggplot(data=y)+
facet_grid(rows=FALT3~FEDA3)+
geom_ribbon(mapping=aes(x=HV007-2000,ymin=LANE,ymax=UANE,fill=NVER),color=NA,alpha=0.2,data=y%>%filter(HV025=="Urbano"))+
geom_ribbon(mapping=aes(x=HV007-2000,ymin=LANE,ymax=UANE,fill=NVER),color=NA,alpha=0.1,data=y%>%filter(HV025=="Rural"))+
geom_line(mapping=aes(x=HV007-2000,y=IANE,color=NVER,linetype=HV025))+
labs(
    x="año, en el siglo XXI",y="prop. prevalencia anemia",
    color="norma",fill="norma",linetype="ámbito",
    title="Figura 2 Tendencias de Prevalencias Subnacionales",
    caption=paste0("INEI/ENDES 6-35m"," n=",nrow(filter(d,HV007!=2024)))
)+
theme_minimal()
LALT$G0102=g
# Figura 3 (para RPMESP)
y=LALT$R4B
g=
ggplot(data=y)+
geom_abline(intercept=0,slope=1,color="gray",linewidth=1,linetype="solid")+
geom_errorbar(mapping=aes(x=PR4,ymin=PR6_low,ymax=PR6_upp),color="pink")+
geom_errorbarh(mapping=aes(y=PR6,xmin=PR4_low,xmax=PR4_upp),color="pink")+
geom_point(mapping=aes(x=PR4,y=PR6),color="red")+
annotate(x=0.475,y=0.475,geom="label",label="identidad",size=2.5,color="gray")+
scale_x_continuous(labels=scales::label_number(decimal.mark=","))+
scale_y_continuous(labels=scales::label_number(decimal.mark=","))+
labs(
    x="prop. prevalencia anemia, norma 2024, fórmula",
    y="prop. prevalencia anemia, norma 2024 tabla",
    title="Figura 3 Prevalencias Regionales según técnica de cálculo",
    caption=
        paste0(
            "INEI/ENDES 6-35m 2009-2023",
```

```
            " n=",sum(y$NNOP)," p=",nrow(y)
        )
)+
theme_minimal()
LALT$G0105=g
} # outc
# ------------------------------------------------------------------------------------------ #
# Funciones Diagnósticas (llamadas para suplemento GLM)
GSR6=function(mm,p,n=NDSIM){
ff=as.character(mm$family)[1]
if(ff=="binomial"){
    r=replicate(n,rbinom(n=length(p),size=1,prob=p))
}else if(ff=="gaussian"){
    r=replicate(n,rnorm(n=length(p),mean=p,sd=sd(residuals(mm))))
}else{
    r=rep(NA,n)
}
return(r)
}
GDX7=function(mm){
ff=as.character(mm$family)[1]
yy=as.character(mm$formula)[2]
bckd=svydiags::svyCooksD(mobj=mm,stvar="IDESP",clvar="IDCLU",doplot=FALSE)
bdff=svydiags::svydffits(mobj=mm,stvar="IDESP",clvar="IDCLU",z=3)
bdfb=svydiags::svydfbetas(mobj=mm,stvar="IDESP",clvar="IDCLU",z=3)
byha=svydiags::svyhat(mobj=mm,doplot=FALSE)
bstr=svydiags::svystdres(mobj=mm,stvar="IDESP",clvar="IDCLU",doplot=FALSE)
bvma=svydiags::Vmat(mobj=mm,stvar="IDESP",clvar="IDCLU")
 # diagnósticos svydiag: inflación de la varianza
l=names(coefficients(mm))
l=l[l!="(Intercept)"]
l=gsub("FTEN2|FTEN3|FTEN4","FTEN",l)
l=gsub("IDDOM2|IDDOM3","IDDOM",l)
l=unique(l)
```

```
l=paste0("~",paste(l,collapse="+"))
bxmm=model.matrix(object=as.formula(l),data=mm$data)
bvif=svydiags::svyvif(mobj=mm,X=bxmm[,-1],w=mm$data$WPOND,stvar="IDESP",clvar="IDCLU")
 #funcionaría solo en binomial, pendiente su resumen
 #bcio=svydiags::svycollinear(mod=mm,w=mm$data$WPOND,Vcov=vcov(mm),svyglm.obj=TRUE)
w=
    tribble(
        ~IPAR,~XVAL,~DSC,
        "NUOBSO",nrow(mm$data),"unweighted n",
        "MCFAR2",1-mm$deviance/mm$null.deviance,"McFadden Pseudo R2",
        "COOKMD",median(bckd),"Median Cook Influence",
        "COOKQ1",quantile(bckd,0.25),"Percentile 25 Cook",
        "COOKQ2",quantile(bckd,0.75),"Percentile 75 Cook",
        "COOKP2",mean(bckd>2),"Proportion Cook>2",
        "COOKP3",mean(bckd>3),"Proportion Cook>3",
        "FITSPX",mean(abs(bdff$Dffits)>bdff$cutoff),"Proportion Fit>cut",
        "BETAPX",mean(abs(bdfb$Dfbetas)>bdfb$cutoff),"Proportion Beta>cut",
        "LEVEP3",mean(byha>3),"Proportion Leverage >3",
        "RESIP3",mean(abs(bstr$stdresids)>3),"Proportion Residuals >3",
        "MCOVPO",mean(bvma!=0),"Proportion Covar Matrix non-zero"
    )
W=
    tibble(
        TERM=labels(coefficients(mm)),
        TVAL=as.numeric(coefficients(mm)),
        BETX=apply(X=abs(bdfb$Dfbetas)>bdfb$cutoff,MARGIN=1,FUN=sum),
    )%>%
    left_join(
        y=
            bvif$`No intercept`%>%
            mutate(FGT3=svy.vif>3,TERM=rownames(bvif$`No intercept`)),
        by="TERM"
    )
```

```
 # # # $`Intercept adjusted`
return(list(w=w,W=W))
}
GDX8=function(mm,NDSIM=30,FNSAM=1){
ff=as.character(mm$family)[1]
yy=as.character(mm$formula)[2]
fc=mm$coefficients
nn=as.integer(nrow(mm$data)*FNSAM)
 # diagnósticos DHARMa indirectos: residuos ponderados
v=mm$data%>%slice_sample(n=nn,weight_by=W1RF,replace=TRUE)
if(!any(is.na(fc))){
    p=predict(object=mm,newdata=v,type="response")
}else{
     # preparación de expresiones para cada término
    fx=fc
    fx[is.na(fx)]=0
    l=names(fx)
    l=gsub("\\(Intercept\\)","1",l)
    l=gsub("\\)","\\)=='",l)
    l[!grepl(":",l)&grepl("factor",l)]=paste0(l[!grepl(":",l)&grepl("factor",l)],"'")
    l[grepl(":",l)&grepl(":factor",l)]=paste0(l[grepl(":",l)&grepl(":factor",l)],"')")
    l[grepl(":",l)&grepl("factor",l)]=gsub("factor","(factor",l[grepl(":",l)&grepl("factor",l)])
    l=gsub(":","')\\*",l)
    names(fx)=l
    l=paste0("(",l,")*(",as.numeric(fx),")")
    fl=paste(l,collapse="+")
    p=
        sapply(
            X=1:nrow(v),
            FUN=function(i){
                return(with(v[i,],eval(expr=parse(text=fl))))
            }
        )
```

```
    if(ff=="binomial"){p=exp(p)/(1+exp(p))}

}

u=eval(expr=parse(text=paste0("v$",yy)))

s=

    DHARMa::createDHARMa(

        simulatedResponse=GSR6(mm=mm,p=p,n=NDSIM),

        fittedPredictedResponse=p,

        observedResponse=u,integerResponse=(ff=="binomial")

    )

print(ggplot()+annotate(x=0.5,y=0.5,geom="label",label=paste(yy,ff))+theme_void())

b1=DHARMa::testDispersion(simulationOutput=s,plot=TRUE)

b2=DHARMa::testQuantiles(simulationOutput=s,plot=TRUE)

b3=DHARMa::testUniformity(simulationOutput=s,plot=TRUE)

b4=DHARMa::testOutliers(simulationOutput=s,plot=TRUE)

o=

    tribble(

        ~IPAR,~XVAL,~DSC,

        "PDISP1",b1$p.value,"p, Nonpar Dispersion test",

        "PQGAM2",b2$p.value,"p, Combined Adjusted Quantile test",

        "POSKS3",b3$p.value,"p, Asymptotic One-Sample Kolmogorov-Smirnov test",

        "POUTB4",b4$p.value,"p, Outlier Exact Binomial test",

        "PEXPE4",as.numeric(b4$null.value),"Expected Proportion of Outliers",

        "POBSE4",as.numeric(b4$estimate),"Observed Proportion of Outliers",

        "PLLOW4",as.numeric(b4$conf.int[1]),"Lower CL Proportion of Outliers",

        "PLUPP4",as.numeric(b4$conf.int[2]),"Upper CL Proportion of Outliers"

    )

return(o)

}

GST0=function(ll,yv,bv){

r=paste0(yv,"~0")

z=

    tibble(

        SVLevel=ll,
```

```
        STTestP=
            sapply(
                X=ll,
                FUN=function(x){
                    p=eval(expr=parse(text=paste0("subset(s,",bv,"==x)")))
                    as.numeric(
                        survey::svyttest(
                            formula=as.formula(r),
                            design=p
                        )$p.value
                    )
                }
            )
    )
return(z)
}
# ------------------------------------------------------------------------------- #
# Documentación
# Lista de variables
# Para cada niño, variables versión RECH6:
# RECH6.HC1 Edad en meses
# RECH0.HV040 Altitud del conglomerado en metros
# RECH0.HV025 Ámbito urbano o rural
# RECH0.HV023 Región administrativa
# RECH0.HV007 Año de la entrevista
# RECH1.HV103 Pernoctó la noche anterior
# RECH0.HV105 Entrevista Completa
# RECH6.HC55 Hemoglobina Completa
# RECH6.HC53 Hemoglobina en sangre, leida
# Para cada niño, variables versión REC44:
# REC44.HW1 Edad en meses
# REC21.B4 Sexo declarado
# RECH0.HV040 Altitud del conglomerado en metros
# RECH0.HV025 Ámbito urbano o rural
# RECH0.HV023 Región administrativa
# RECH0.HV007 Año de la entrevista
```

```
# REC44.HW53 Hemoglobina en sangre, leida
# Además se tomaron variables del diseño muestral:
# RECH0.HV022 Estrato
# RECH0.HV001 Conglomerado
# RECH0.HHID Vivienda, Hogar
# REC44.CASEID Vivienda, Hogar, Madre (y REC21)
# REC44.HWIDX Sujeto (y REC21, RECH6)
# RECH0.HV005 Factor de Expansión (HV005X 2015 y HV005A 2020)
# Enlaces:
# RECH0 --< REC44 -- REC21 HHID(CASEID) -< HWIDX
# RECH0 --< RECH1 -- RECH6 HHID -< HWIDX = HC0
# Salidas, versión RECH6:
# LALT Datos, Estimaciones, Tablas, Gráficos
# DAT Datos
# SVY Diseño Muestral, para survey
# RY0 Descripción de la Inclusión por Regiones
# RZ0 Descripción de la Muestra por Años, Antes de Exclusión
# R0 Estimaciones Nacionales por año
# R4B Estimaciones por región
# R4C Estimaciones por región-ámbito-año
# R8 Estimaciones de indicadores
# R9 Estimaciones por altitud-ámbito-año
# RMFIN Modelos Finales (saturados)
# RMRES Modelos Finales - resumenes
# RMTER Modelos Finales - términos
# T0102 Prevalencia nacional de anemia, anual
# T0103 Prevalencia nacional de anemia, regional
# T0104 Resumen de los Dos Modelos Principales
# G0101 Tendencias de prevalencia nacional
# G0102 Tendencias de prevalencias subnacionales
# G0105 Prevalencias regionales según técnica de cálculo
# FTES Lista de Archivos ENDES usados
# Salidas, versión REC44:
# LEST Estimaciones
# RY0 Descripción de la Inclusión por Regiones
# RZ0 Descripción de la Muestra por Años, Antes de Exclusión
# R0 Estimaciones Nacionales
# R1 Estimaciones por grupo de edad
# R2 Estimaciones por dominio
# R3 Estimaciones por dominio-año
# R4 Estimaciones por región-año
```

```
# R4A Estimaciones por región ámbito
# R4B Estimaciones por región
# R4C Estimaciones por región-ámbito-año
# R4D Identificación de regiones extremas
# R5 Estimaciones por grupo de altitud
# R8 Estimaciones de indicadores
# R9 Estimaciones por altitud-ámbito-año
# RMFIN Modelos Finales (saturados)
# RMRES Modelos Finales - resumenes
# RMTER Modelos Finales - términos
# LTAB Tablas
# T0101 Encuesta Demográfica y de Salud Familiar
# T0102 Prevalencia nacional de anemia, anual
# T0103 Prevalencia nacional de anemia, regional
# LGRA Gráficos
# G0101 Tendencias de prevalencia nacional
# G0102 Tendencias de prevalencias subnacionales
# G0103 Prevalencias por región-año según normas
# G0104 Prevalencias por región-años según OMS 2024
# G0105 Prevalencias regionales según técnica de cálculo
# ------------------------------------------------------------------------------- #
# License:
#
# The data sources for this analysis come from publications and data by
# the Instituto Nacional de Estadística e Informática (INEI) from Peru,
# which can be freely obtained at https://proyectos.inei.gob.pe/microdatos/
# and https://www.datosabiertos.gob.pe/ under a ODC Open Database License.
# They can also be freely obtained from https://dhsprogram.com/Data/ .
# Consolidated and anonymized data, as used for the analysis,
# are freely available in github/vipermcs.
#
# This R program has been developed by Miguel Campos,
# on the basis of discussion with the co-authors.
# It is placed freely available, with the sole condition of quoting the source.
# Copyright (c) 2024 Miguel Campos under MIT License
#
# Permission is hereby granted, free of charge, to any person obtaining a copy
# of this software and associated documentation files (the "Software"), to deal
# in the Software without restriction, including without limitation the rights
# to use, copy, modify, merge, publish, distribute, sublicense, and/or sell
# copies of the Software, and to permit persons to whom the Software is
```

```
# furnished to do so, subject to the following conditions:
#
# The above copyright notice and this permission notice shall be included in all
# copies or substantial portions of the Software.
#
# THE SOFTWARE IS PROVIDED “AS IS”, WITHOUT WARRANTY OF ANY KIND, EXPRESS OR
# IMPLIED, INCLUDING BUT NOT LIMITED TO THE WARRANTIES OF MERCHANTABILITY,
# FITNESS FOR A PARTICULAR PURPOSE AND NONINFRINGEMENT. IN NO EVENT SHALL THE
# AUTHORS OR COPYRIGHT HOLDERS BE LIABLE FOR ANY CLAIM, DAMAGES OR OTHER
# LIABILITY, WHETHER IN AN ACTION OF CONTRACT, TORT OR OTHERWISE, ARISING FROM,
# OUT OF OR IN CONNECTION WITH THE SOFTWARE OR THE USE OR OTHER DEALINGS IN THE
# SOFTWARE.
# -------------------------------------------------------------------------------- #
```
