# Supplementary material for: New world health organization guideline on anemia cut-off points: implications for children aged 6-35 months in Peru
Source: Rev Peru Med Exp Salud Publica. 2025 Jun 12;42(2):115–25. doi: 10.17843/rpmesp.2025.422.14028 (PMC12377884; doi:10.17843/rpmesp.2025.422.14028)
Supplement: Translation

# Translation: Nueva directriz de la Organizacion Mundial de la Salud sobre los puntos de corte de anemia: implicancias en niños de 6-35 meses de Perú

**Authors:** Miguel Campos-Sánchez, Luis Cordero-Muñoz, Enrique Velásquez-Hurtado, Nelly Baiocchi-Ureta, Marianella Miranda-Cuadros, María Inés Sánchez-Griñán, Walter Valdivia-Miranda

**Affiliations:** 1 Universidad Peruana Cayetano Heredia, Lima, Perú.; 2 Instituto Nacional de Salud, Lima, Perú.; 3 CERES-Nutrir, Lima, Perú.; 4 Ministerio de Desarrollo e Inclusión Social, Lima, Perú.

**Keywords:** Hemoglobina, Anemia, Niños, Perú

## Abstract

**Objetivos.:**

Comparar las prevalencias nacionales anuales y regionales de anemia, usando la norma 2001 versus la nueva norma 2024 en niños entre 6 y 35 meses de edad residentes en Perú entre 2009 y 2023. Evaluar si las diferencias entre normas varían por región, ámbito o año.

**Materiales y métodos.:**

Análisis secundario de la Encuesta Demográfica y de Salud Familiar (muestra aleatoria nacional continua, estratificada y con conglomerados). La hemoglobina se midió en sangre capilar con Hemocue. Para cada norma se aplicó una ecuación (y/o tabla) de ajuste por altitud y un punto de corte. Se calcularon intervalos de 95% de confianza [IC95]. Se evaluaron diferencias según regiones, ámbitos y/o años con un modelo lineal generalizado calculando extremos y cuartiles. Estimaciones y modelos fueron ponderados.

**Resultados.:**

Se analizaron 120 711 niños. La diferencia en prevalencias fue -6,3 [-6,6 a -6,0], p<0,001, varió según región (p<0,001), región-año (p=0,004), y región-ámbito (p<0,001), entre -40,6 y 11,0. El porcentaje de niños cuyo diagnóstico difiere fue de 11,0 [ 10,7 a 11,2], (p<0,001), varió entre 0,0 y 40,6. La diferencia tabla versus ecuación fue -3,8 [ -4,0 a -3,6].

**Conclusiones.:**

La prevalencia difiere con la nueva norma (generalmente disminuye, pero puede aumentar), diferencia variable según región, ámbito y año. El porcentaje cuyo diagnóstico difiere también varía. Estas diferencias son de gran importancia sanitaria, en algunos casos cambian el problema de severo a moderado. El cálculo con tabla subestima el obtenido con ecuación. La literatura sustenta la dirección de la corrección, pero no su magnitud.

## INTRODUCCIÓN

La anemia infantil es reconocida como un problema de gran importancia ^1^^,^^2^ especialmente por sus consecuencias sobre el desarrollo temprano. En el Perú ha sido y es de suma prioridad sanitaria ^2^^-^^4^. Su frecuencia se determina comparando la hemoglobina en sangre con los puntos de corte ^1^^,^^2^.

La Organización Mundial de la Salud (OMS) publicó el 6 de marzo del 2024 una nueva norma («guideline») ^5^ sobre los puntos de corte de la hemoglobina para diagnosticar la anemia, actualizando la norma previa ^6^ y tomando en cuenta importante evidencia publicada recientemente ^7^^-^^9^. La nueva norma tiene tres cambios principales para niños pequeños: reduce el punto de corte para el grupo de 6 a 23 meses de edad, modifica la ecuación de ajuste por altitud, reduciendo el punto de corte por encima de los 3000 m s. n. m., aumentándolo por debajo de esa altitud y recomienda como método de laboratorio la medición en sangre venosa con instrumentos hematológicos automatizados o portátiles calibrados, ambos bajo control de calidad. Perú ha incorporado oficialmente la norma 2024 en la actualización de su norma técnica ^10^.

La norma anterior ^6^ carecía de evidencia sólida para sus puntos de corte, siendo esencialmente opinión de expertos con mención a unos pocos estudios. Ya desde hace algún tiempo se han señalado indicios de que la norma 2001 y sus predecesoras sobreestimaban los puntos de corte en los primeros meses de vida ^11^ y en los ajustes por altitud ^9^, resultando en una probable sobreestimación de las prevalencias de anemia.

Al respecto se ha producido un extenso debate internacional ^11^^,^^12^ y nacional ^3^^,^^4^. Nuestra interpretación del consenso hasta 2023 es reconocer que la evidencia sustentaba la necesidad de modificar las normas, así como la dirección en que los ajustes en los puntos de corte tendrían que hacerse, pero no sustentaba la magnitud de las correcciones necesarias.

La elección de valores normales de hemoglobina según edad y altitud podría sustentarse en estudios de cohorte en muestras representativas de grandes poblaciones con estudios complementarios. Las nuevas normas emplean en cambio lo que llaman «enfoque estadístico» (bastante usado en patología clínica), es decir la estimación de los percentiles de la distribución en encuestas representativas nacionales (y algunos estudios no representativos) con variables (marcadores de hierro e inflamación y síntomas) que permitan seleccionar un subconjunto «sano». Dicho enfoque puede fallar crucialmente cuando las prevalencias de algunas causas de anemia no sean pequeñas como para ignorarlas y no se disponga de métodos diagnósticos para excluirlas.

Los puntos de corte de la nueva norma 2024 citan tres artículos ^7^^-^^9^ (que, en conjunto, representan aproximadamente algo menos de 11% de la población mundial) y otros dos artículos ^13^^,^^14^ no considerados. Hay también estudios previos ^15^^-^^25^ con indicios de discrepancias con la norma 2001, un estudio peruano reciente ^26^ y propuestas alternativas ^27^^-^^30^. Revisaremos esta evidencia con más detalle en la sección de discusión.

La implementación de la nueva norma OMS 2024 cambiará las cifras de prevalencia. No están precisados aún la magnitud de esos cambios, su efecto sobre las tendencias temporales, su homogeneidad dentro del país y sus efectos en la toma de decisiones. Como consecuencia de no conocer esas magnitudes, se propuso el presente estudio con los siguientes objetivos (a) comparar las prevalencias nacionales anuales de anemia, cuando se utiliza la norma OMS 2001 versus OMS 2024 para la población de niños entre 6 y 35 meses de edad residentes en el Perú entre 2009 y 2023; (b) comparar las prevalencias regionales acumuladas; y (c) evaluar si la magnitud de las diferencias varía según región, ámbito (urbano o rural) o año calendario, solos o en combinación.

**MENSAJE CLAVE**

Motivación para realizar el estudio. La Organización Mundial de la Salud modificó en 2024 los puntos de corte que definen anemia. No se conoce la magnitud del cambio en la prevalencia en niños de 6-35 meses con anemia en el Perú, respecto de la norma 2001.

Principales hallazgos. Entre 2009-2023 encontramos diferencias importantes y heterogéneas (a) nacionales, (b) entre y dentro de regiones; y (c) por la técnica de cálculo (tabla o ecuación).

Implicancias. Los fundamentos de la norma 2024, siendo mucho mejores que la 2001, no son suficientes. Es necesario adoptar la nueva norma con cautela, tanto en la atención individual como en las decisiones poblacionales.

## MATERIALES Y MÉTODOS

El presente diseño corresponde a un estudio diagnóstico que compara las prevalencias de anemia bajo dos normas, OMS 2001 y 2024, mediante un análisis secundario de los datos de la Encuesta Demográfica y de Salud Familiar (ENDES) ^31^ 2009 al 2023, ejecutada por el Instituto Nacional de Estadística e Informática (INEI), quinquenal desde el 1986 y continua anual desde el 2004.

### Población y muestra

La ENDES ^31^^,^^32^ tiene un diseño transversal repetido sobre una muestra aleatoria nacional. El marco, mantenido por el INEI, agrupa todo el país en conglomerados de aproximadamente 120-140 viviendas, distribuidos en estratos por regiones (primer nivel de subdivisión administrativa del país) y niveles socioeconómicos. El INEI calcula el tamaño muestral como encuesta multipropósito resultando aproximadamente 35 000 hogares cada año. En la primera etapa, en cada estrato se seleccionan aleatoriamente conglomerados con probabilidad proporcional al tamaño y se distribuyen entre los meses calendario. En la segunda etapa, los equipos de campo actualizan la relación de hogares en cada conglomerado y seleccionan aleatoriamente los entrevistados. La edad (fecha de nacimiento) y el sexo se obtienen declarativamente. El ámbito rural se define cuando el centro poblado que incluye al hogar tiene menos de 2000 habitantes. La altitud (m s. n. m., metros sobre el nivel del mar) es definida por el INEI para cada conglomerado (desde 2016 el INEI recoge altitud del hogar con GPS, pero ese dato no es usado aquí). En submuestras de varios subgrupos poblacionales se mide la hemoglobina en sangre capilar mediante hemoglobinómetros portátiles (el personal se estandariza al menos una vez al año). El INEI actualiza continuamente el marco reajustando la definición y técnicas de muestreo. Los datos, individuales anonimizados y la documentación están públicamente disponibles.

Los datos individuales fueron leídos y consolidados, reajustando la ponderación de cada año según la proyección poblacional nacional ^33^ y redefiniendo estratos como las 25 regiones × 2 ámbitos (urbano y rural). Se incluyeron niños entre 6 y 35 meses de edad con hemoglobina completa, que pernoctaron la noche anterior, un total de 120 711 niños.

### Variables

Para cada niño se tomaron de la ENDES las siguientes variables del cuestionario de hogares, archivo RECH0 (y, donde se indica, de los módulos de niños en el cuestionario individual, archivos REC21 y RECH6): Edad en meses cumplidos (RECH6.HC1), altitud del conglomerado en m s. n. m. (HV040), ámbito urbano o rural (HV025), región administrativa (HV023, con la provincia del Callao separada y la provincia de Lima incluida en la región Lima), año de la entrevista (HV007), hemoglobina g/dl (Hb) en sangre capilar medida con dispositivo portátil Hemocue Hb201 (RECH6.HC53), estrato (HV022), conglomerado (HV001), vivienda y hogar (HHID), niño (RECH6.HC1), y factor de expansión (HV005 excepto HV005X para 2015 y HV005A para 2020). El archivo principal de análisis es RECH6 al cual se enlazaron los archivos REC21 y RECH0 mediante CASEID y/o HHID.

### Análisis

Para cada niño incluido se calculó la condición dicótoma de anemia con ambas normas ^5^^,^^6^ restando a la Hb medida un ajuste de altitud antes de compararla con un punto de corte. Para la norma 2001 el ajuste fue la ecuación del Centers for Disease Control (CDC) ^23^^,^^34^ (desde los 1000 m s. n. m. a partir del 2022) y el corte fue 11 g/dL. Para la norma 2024 el ajuste fue la ecuación especificada en la norma ^8^^,^^9^ desde 1 m s. n. m. y el corte fue 10,5 (edad <24 meses) u 11 (edad 24-35 meses) g/dl. Se calculó también una variante de ajuste de altitud con la tabla, no la ecuación, especificada en la norma 2024.

Las estimaciones fueron ajustadas al diseño muestral ^35^ con sus respectivos intervalos de 95% de confianza (IC95). Para ilustrar las implicancias, se estimaron también las prevalencias anuales ajustadas combinando tres agrupaciones: edad (6-11,12-23 y 24-35 meses), altitud (0-999, 1000-2999 y 3000+ m s. n. m.) y ámbito (urbano, rural).

Se calculó la diferencia de diagnósticos en cada individuo como la resta entre sus diagnósticos de anemia (codificados 0 sin, 1 con) con la norma 2024 menos la norma 2001. La diferencia promedio en prevalencia fue estimada como el promedio de las diferencias de diagnósticos individuales, con su respectivo IC95. Por definición, dichas diferencias promedio son distintas de cero para la edad y la altitud, y por consiguiente no comparamos estadísticamente estas variables. Evaluamos más bien las diferencias promedio según regiones, ámbitos y/o años (que son consecuencia de sus distribuciones de edad y altitud). Para ello aplicamos un GLM (modelo lineal generalizado, con distribución normal y ajustado al diseño muestral), siendo la unidad de análisis el niño encuestado, la variable dependiente la diferencia individual y las variables independientes las tres variables categóricas, en un modelo saturado (con todas las interacciones de 2 y 3 términos). Para apreciar la variabilidad calculamos los extremos y cuartiles de la distribución de diferencias estimadas en las combinaciones de región, ámbito y año. Cuando la prevalencia es mayor a 40% la clasificación oficial de OMS ^1^ pasa de problema de salud pública moderado a severo, y recomienda suplementación diaria universal en el rango 6-23 meses.

De manera complementaria estimamos también el porcentaje nacional de niños cuyo diagnóstico difiere bajo las dos normas y la diferencia en las prevalencias cuando se calcularon con la norma OMS 2024 de dos maneras (ecuación versus tabla).

Se empleó el programa R ^36^ versión 4.4.2 con los paquetes tidyverse, survey y sus dependencias. El programa y los datos consolidados de la prepublicación están en https://github.com/vipermcs/pdata/blob/main/CUTHBN32024.zip. Se adjunta un suplemento con las fuentes ENDES, los modelos GLM y el programa R.

### Aspectos éticos

El estudio empleó solamente datos individuales anonimizados públicamente disponibles, por lo que no solicitamos revisión por parte de algún comité de ética en investigación.

## RESULTADOS

La figura 1 presenta la tendencia de la prevalencia nacional anual bajo las dos normas. Puede apreciarse que dicha tendencia es similar con ambas normas (dentro de los márgenes de confianza), y que con la nueva norma la prevalencia ya no se sitúa como problema de salud pública severo sino moderado. La tabla 1 presenta las cifras de prevalencia nacional anual de la figura 1 y agrega las estimaciones de la diferencia de prevalencias. Puede apreciarse que la magnitud de la diferencia nacional es similar a lo largo de los años. Cada año es diferente de 0 (p<0,001) sin diferencias entre los años (p=0,067).

**Figura 1:** Prevalencias anuales de anemia en niños de 6-35 meses, Perú 2009-2023

El eje horizontal es el año calendario. El eje vertical es la prevalencia de anemia. Las líneas unen los estima-dos puntuales anuales, ponderados. Las bandas unen los límites de 95% de confianza. La línea horizontal gris separa problema de salud pública severo y moderado, según la definición OMS. El color corresponde a cada norma OMS, la verde es la anterior 2001 y la roja es la nueva 2024.

**Tabla 1:** Prevalencias porcentuales anuales de anemia en niños 6-35 meses, Perú 2009-2023.

Año	Prev. Norma 2001	Prev. Norma 2024	Dif. Prev. 2024 - 2001
2009	50,4 (48,1 a 52,6)	43,2 (41,0 a 45,4)	-7,2 (-8,6 a -5,7)
2010	50,3 (48,1 a 52,5)	43,4 (41,2 a 45,6)	-6,9 (-8,2 a -5,6)
2011	41,6 (39,5 a 43,7)	35,6 (33,6 a 37,6)	-6,0 (-7,1 a -4,9)
2012	44,5 (42,3 a 46,6)	38,6 (36,5 a 40,7)	-5,9 (-7,1 a -4,6)
2013	46,4 (44,3 a 48,4)	39,7 (37,5 a 41,8)	-6,7 (-8,2 a -5,3)
2014	46,8 (44,8 a 48,9)	40,1 (38,1 a 42,1)	-6,8 (-8,1 a -5,4)
2015	43,5 (42,3 a 44,8)	37,4 (36,2 a 38,6)	-6,1 (-6,9 a -5,3)
2016	43,6 (42,2 a 45,0)	38,3 (36,9 a 39,6)	-5,3 (-6,1 a -4,5)
2017	43,6 (42,2 a 45,0)	37,4 (36,1 a 38,7)	-6,2 (-7,1 a -5,3)
2018	43,5 (42,3 a 44,8)	38,5 (37,2 a 39,7)	-5,1 (-5,9 a -4,3)
2019	40,3 (39,0 a 41,6)	34,2 (32,9 a 35,4)	-6,1 (-7,0 a -5,3)
2020	40,1 (38,3 a 41,9)	32,8 (31,1 a 34,5)	-7,3 (-8,4 a -6,2)
2021	39,0 (37,8 a 40,3)	30,9 (29,7 a 32,1)	-8,1 (-8,9 a -7,3)
2022	42,4 (41,2 a 43,7)	36,5 (35,3 a 37,7)	-5,9 (-6,8 a -5,1)
2023	43,1 (41,8 a 44,4)	37,7 (36,4 a 38,9)	-5,4 (-6,3 a -4,6)
2009-2023			-6,3 (-6,6 a -6,0)

Formato: estimado (límites inferior a superior de 95% de confianza). Prev: prevalencia. Dif: diferencia entre prevalencias Norma 2024 menos 2001.

La tabla 2 presenta las prevalencias regionales, acumuladas para todo el periodo 2009-2023, bajo las dos normas. Puede apreciarse la variación en la magnitud de las diferencias. Cada una de las regiones, excepto Cajamarca y Moquegua, es diferente de 0 (p<0,001) con diferencias entre regiones (p<0,001). La tabla 2 también presenta los porcentajes de niños cuyo diagnóstico cambia para cada región, también acumulados durante 2009-2023. Cada una de las regiones es mayor que 0 (p<0,001) con diferencias entre regiones (p<0,001).

**Tabla 2:** Prevalencias porcentuales regionales de anemia en niños de 6-35 meses, Perú 2009-2023.

Región	Prev. Norma 2001	Prev. Norma 2024	Dif. Prev. 2024 - 2001	Porc. Cambian Dx. (anemia→normal o normal→anemia) entre 2001 y 2024
Amazonas	46,1 (44,3 a 47,9)	48,9 (47,1 a 50,7)	+2,8 (+1,8 a +3,8)	8,8 (7,9 a 9,8)
Ancash	44,0 (42,2 a 45,9)	36,3 (34,6 a 38,1)	-7,7 (-8,8 a -6,6)	10,6 (9,5 a 11,7)
Apurimac	53,9 (51,8 a 55,9)	43,2 (41,2 a 45,2)	-10,7 (-12,0 a -9,3)	14,1 (12,8 a 15,3)
Arequipa	41,2 (39,2 a 43,3)	42,5 (40,4 a 44,6)	+1,3 (+0,1 a +2,4)	8,4 (7,5 a 9,4)
Ayacucho	50,4 (48,5 a 52,2)	44,2 (42,3 a 46,0)	-6,2 (-7,5 a -4,9)	11,7 (10,5 a 12,9)
Cajamarca	40,2 (38,2 a 42,2)	40,3 (38,4 a 42,3)	+0,1 (-1,0 a +1,3)	8,4 (7,3 a 9,4)
Callao	39,3 (36,8 a 41,9)	31,7 (29,4 a 34,0)	-7,6 (-9,0 a -6,2)	9,4 (7,8 a 11,0)
Cusco	56,6 (54,5 a 58,7)	43,6 (41,5 a 45,7)	-13,1 (-14,6 a -11,5)	15,0 (13,5 a 16,4)
Huancavelica	58,8 (56,7 a 60,9)	41,1 (38,8 a 43,3)	-17,7 (-19,4 a -16,0)	18,6 (17,0 a 20,3)
Huanuco	48,0 (46,1 a 49,9)	43,1 (41,2 a 44,9)	-5,0 (-6,5 a -3,5)	12,5 (11,3 a 13,7)
Ica	40,7 (39,0 a 42,4)	38,3 (36,6 a 40,1)	-2,3 (-3,3 a -1,4)	9,0 (8,2 a 9,9)
Junin	52,4 (50,5 a 54,3)	44,6 (42,6 a 46,5)	-7,8 (-9,1 a -6,5)	12,5 (11,4 a 13,7)
La Libertad	39,6 (37,7 a 41,5)	31,8 (30,0 a 33,6)	-7,8 (-9,0 a -6,6)	10,3 (9,2 a 11,4)
Lambayeque	37,4 (35,8 a 39,0)	31,0 (29,4 a 32,7)	-6,4 (-7,4 a -5,4)	10,1 (9,2 a 11,1)
Lima	34,8 (33,7 a 35,9)	29,5 (28,4 a 30,6)	-5,3 (-5,9 a -4,6)	9,2 (8,5 a 9,8)
Loreto	55,9 (54,4 a 57,5)	48,5 (46,9 a 50,1)	-7,4 (-8,3 a -6,5)	10,8 (9,9 a 11,7)
Madre de Dios	58,3 (56,6 a 60,0)	55,5 (53,8 a 57,1)	-2,8 (-3,8 a -1,8)	9,9 (8,9 a 10,9)
Moquegua	38,4 (36,2 a 40,5)	38,4 (36,3 a 40,5)	+0,0 (-1,2 a +1,3)	9,3 (8,2 a 10,3)
Pasco	56,5 (54,6 a 58,3)	43,6 (41,6 a 45,6)	-12,9 (-14,7 a -11,0)	18,8 (17,4 a 20,3)
Piura	41,1 (39,6 a 42,7)	35,6 (34,1 a 37,1)	-5,5 (-6,5 a -4,5)	10,2 (9,3 a 11,1)
Puno	73,8 (72,1 a 75,6)	47,2 (45,1 a 49,4)	-26,6 (-28,4 a -24,8)	27,2 (25,4 a 28,9)
San Martin	43,3 (41,6 a 45,0)	44,0 (42,2 a 45,8)	+0,7 (-0,2 a +1,7)	8,8 (8,0 a 9,7)
Tacna	38,0 (36,1 a 40,0)	37,5 (35,6 a 39,4)	-0,5 (-1,7 a +0,7)	9,0 (7,9 a 10,1)
Tumbes	47,2 (45,5 a 48,8)	40,0 (38,2 a 41,7)	-7,2 (-8,3 a -6,0)	10,6 (9,5 a 11,6)
Ucayali	58,3 (56,7 a 59,8)	52,2 (50,6 a 53,9)	-6,1 (-7,0 a -5,1)	10,7 (9,9 a 11,6)

Formato: estimado (inferior a superior). Prev: prevalencia. Dif: diferencia entre prevalencias Norma 2024 menos 2001. Porc: porcentaje. Dx: diagnóstico.

El cambio de diagnóstico (quinta columna) incluye aquellos que pasan de anémico a no anémico como los que pasan de no anémico a anémico.

La figura 2 presenta la tendencia en la prevalencia a lo largo del tiempo con ambas normas para cada combinación de ámbito, grupo de edad y nivel de altitud. Puede apreciarse que, en niños de 24 meses de edad o más, en quienes el punto de corte de ambas normas es el mismo, se producen diferencias en la estimación de prevalencia de anemia entre las normas, por la modificación de la ecuación de ajuste según altitud. Dichas diferencias no son lineales, e incluso presentan un incremento en la prevalencia de anemia cuando la altitud está por debajo de los 3000 m s. n. m. En niños menores de 24 meses la diferencia de prevalencia de anemia entre las normas resulta tanto del cambio en la ecuación de ajuste según altitud, como de la reducción del punto de corte a nivel del mar en dicho grupo de edad.

**Figura 2:** Prevalencias anuales de anemia en subdominios en niños 6-35 meses, Perú 2009-2023.

Cada panel corresponde a una combinación de grupo de edad (eje superior) y grupo de altitud (eje derecho). El eje inferior es el año calendario. El eje izquierdo es la prevalencia de anemia. Las líneas unen los estimados puntuales anuales, ponderados. Las bandas unen los límites de 95% de confianza. El tipo de línea corresponde al ámbito, urbano (trazo continuo) o rural (trazo con guiones). Las líneas horizontales grises separan problemas de salud pública severo y moderado, según la definición OMS. El color corresponde a cada norma OMS, la verde es la anterior 2001 y la roja es la nueva 2024.

La diferencia en la prevalencia nacional (norma 2024 menos norma 2001) para todo el periodo es de -6,3% (IC95 de -6,6 a -6,0, p<0,001). Dicha diferencia varía significativamente (tabla 3) según año, región, región-año, y región-ámbito, variabilidad reflejada en su distribución en 735 combinaciones de región, ámbito y año, oscilando entre -40,6 y 11,0, con cuartiles en -9,9, -5,8 y -1,1, abarcando un amplio rango de diferencias con distintas implicancias en la práctica.

**Tabla 3:** Resumen del modelamiento.

Variable de resultado (outcome)	Modelo 1^a^	Modelo 2^a^
	Diferencia	Cambio
Número de observaciones individuales	120711	120711
Deviance, nulo	12777	83576
Grados de libertad, nulo	120710	120710
Criterio de información de Akaike, AIC	68471	82742
Criterio de información Bayesiano, BIC	20889	89665
Deviance	12266	81042
Grados de libertad, residuo	33386	33386
Pseudo-R cuadrado, McFadden	0,040	0,030
Año, p	0,014	0,005
Región, p	<0,001	<0,001
Ámbito, p	0,708	<0,001
Año:Región, p	0,006	0,084
Año:Ámbito, p	0,251	0,405
Región:Ámbito, p	<0,001	<0,001
Año:Región:Ámbito, p	0,234	0,047

a Modelos lineales generalizados. Diferencia: diferencia entre diagnósticos individuales norma 2001 versus 2024. Cambio: individuo cambia de diagnóstico según normas 2001 y 2024. p: valores de p para hipótesis de coeficiente cero. Símbolo «:» denota interacción.

El porcentaje, nacional para todo el periodo, de niños cuyo diagnóstico cambia bajo las dos normas es de 11,0 (IC95 de 10,7 a 11,2, p<0,001). Dicho porcentaje varía significativamente (tabla 3) según año, región, ámbito, región-ámbito y año-región-ámbito, variabilidad reflejada en su distribución en 735 combinaciones de región, ámbito y año, oscilando entre 0 y 40,6, con cuartiles en 8,3, 10,7 y 14,1, abarcando también un amplio rango de cambios.

La norma 2024 describe dos maneras de obtener el ajuste por altitud: una tabla de clasificación y una ecuación. La tabla no define ajuste para altitudes de 5000 m s. n. m. o más (la norma 2001 presentaba ambas maneras y por debajo de los 1000 m s. n. m. no aplicaba ningún ajuste). Graficando ajuste versus altitud, la ecuación es una curva continua, mientras que la tabla es una línea escalonada. La diferencia nacional entre ambas maneras es -3,8 (IC95 de -4,0 a -3,6). La figura 3 compara las prevalencias regionales (acumuladas para todo el periodo 2009-2023) de ambas maneras. Encontramos que la prevalencia obtenida con la norma 2024 de ambas maneras discrepa de manera importante. Usar la tabla produce subestimaciones de grado variable en la prevalencia.

**Figura 3:** Prevalencias regionales de anemia según tipo de ajuste de altitud en niños de 6-35 meses, Perú 2009-2023.

Cada punto rojo es un estimado ponderado de la prevalencia regional de anemia. El eje horizontal es la estimación usando la ecuación de la norma 2024. El eje vertical es la estimación usando la tabla de la norma 2024. Las líneas verticales y horizontales rosa son los intervalos de 95% de confianza. La línea diagonal gris es la esperada si ambas técnicas de cálculo fueran equivalentes.

## DISCUSIÓN

Con la nueva norma nuestro análisis encuentra diferencias importantes y estadísticamente significativas en la prevalencia (generalmente reducción, pero también incremento) en grado variable según región, ámbito y año, con tendencias temporales similares, pero no idénticas. Esto resulta de la no linealidad de las normas, la distribución interna de la población por altitud y la cercanía de las posiciones poblacionales a los puntos de corte. Es lógico que la nueva norma difiera, lo que nuestro análisis aporta es la apreciación de su magnitud y variabilidad en una población real y diversa.

En los siguientes párrafos revisamos los artículos de sustento de la nueva norma. Un artículo es sobre la edad ^7^ de 6 meses a 65 años. Sus fuentes de datos relevantes (1999-2019) son dos encuestas (EE. UU. y Ecuador), y una cohorte (Canadá/TARGet Kids!). Dicho análisis ajusta una curva en el segundo semestre de vida, que refleja mejor la fisiología que la transición abrupta de las normas, generadora de un salto artificial a los 24 meses.

Dos artículos son sobre la altitud en niños <60 meses y mujeres de 15-49 años ^9^ y niños en edad escolar ^8^. Sus fuentes de datos relevantes (1999-2019, proyecto BRINDA) son encuestas (México, Colombia, Ecuador, Afganistán, Nepal, Laos, Malawi, Papúa Nueva Guinea, Azerbaijan, Georgia, Reino Unido y EE. UU.), una vigilancia (Guatemala/SIVESNU), y dos evaluaciones (Ghana/GIFTS y Bolivia/NIDI El Alto). Además, la norma 2024 describe una ampliación del análisis, que agregaría datos de 5 años de la vigilancia Perú/SIEN (la única fuente por encima de 4000 m s. n. m.). En niños <60 meses encuentran ^9^ cierta heterogeneidad entre países. La ecuación de la norma 2024 es casi idéntica a la propuesta para escolares ^8^.

Hay dos artículos, mencionados en la norma 2024, pero no considerados por diferentes criterios de inclusión. Una revisión de BRINDA ^13^ es evidencia precursora que examina puntos de corte para el grupo 6-59 meses en conjunto y encuentra una amplia heterogeneidad y una común discrepancia de la norma 2001. Una encuesta en India ^14^ que, con una intención y enfoque (mas no método) similares a la OMS, propone puntos de corte marcadamente inferiores a las normas 2001 y 2024.

Hay además estudios sustentando la existencia de discrepancias con la norma 2001 según edad ^15^^-^^21^^,^^11^ y altitud ^22^^,^^23^, así como la heterogeneidad de dichas discrepancias ^24^^,^^9^^,^^11^, más no su magnitud. De particular interés son los indicios de que el ajuste por altitud varía con la adaptación evolutiva ^25^.

Un estudio peruano reciente ^26^, cuya muestra (6-8 meses a nivel del mar) es muy pequeña y cuya representatividad es discutible (niños de establecimientos de salud sin documentar selección aleatoria), incluyó, además de Lima, las ciudades de Arequipa, Cusco y Puno. Su regresión exponencial produce un ajuste diferente a la norma 2024. Es probable que publiquen otros grupos de edad en el futuro cercano.

Hay finalmente propuestas alternativas de ajuste ^27^^-^^30^ cuyo sustento es muy débil por ser de representatividad limitada por el ámbito y la capacidad de excluir patología.

Nuestra interpretación es que la evidencia actual, si bien sugiere fuertemente que la norma 2024 aplica correcciones en la dirección apropiada, no es suficiente para sustentar que sea el valor óptimo y universal de hemoglobina. Las dudas más importantes son la representatividad de las poblaciones de referencia, los criterios de selección y exclusión, la suavidad de la función de ajuste con la edad y la heterogeneidad entre y dentro de los diferentes países.

Siendo extremadamente deseable sustentar decisiones con evidencia científicamente sólida, en la práctica de la salud pública ello no parece generalmente posible. Tomar decisiones bajo incertidumbre se hace necesario, pero con precauciones razonables para gestionar oportunamente las fallas de estrategia y tácticas. Los indicios de la necesidad de corrección son cada vez más fuertes, pero la evidencia definitiva sobre la magnitud de dicha corrección puede tardar varios años de investigaciones y resultará casi seguramente en modificaciones adicionales.

Los vacíos de evidencia son retos para la investigación a corto y largo plazo. Varios de los estudios que ahora son evidencia parcial podrían ser aprovechados como líneas basales de cohortes para medir el riesgo de diferentes resultados como función de la hemoglobina, edad, altitud y otras covariables.

Para este artículo aplicamos definiciones oficiales ^37^ del indicador de anemia, obteniendo prevalencias nacionales anuales con la norma 2001 idénticas a las oficiales ^38^ a tres dígitos significativos, excepto en 2019-2021 que difieren 0,1 a 0,2. Atribuimos las discrepancias remanentes al software de muestras complejas y pequeñas ambigüedades de documentación ^37^^-^^39^. Las definiciones oficiales toman como universo a los niños que pernoctaron el día anterior, aplican una ponderación especial para los años 2015 y 2020, aplican el ajuste por altitud desde los 1000 m s. n. m. solo desde el año 2022, y convierten metros a pies usando el factor 3,3. Nuestros resultados sobre la comparación entre normas son los mismos de nuestra prepublicación ^40^.

Respecto de la literatura relacionada a nuestro estudio, la norma OMS 2024 ^5^ se publicó en marzo y el INEI publicó los datos anuales de ENDES ^32^ en mayo. Desde nuestra prepublicación ^40^ del 29 de mayo, tras búsquedas en PubMed, BVS y Google, solo hemos encontrado tres comparaciones de las normas. La primera ^41^ usó el registro SIEN 2012-2017 (años combinados) para atenciones de niños 6-23 meses en establecimientos de salud de Lima Metropolitana, encontrando una clara reducción de la prevalencia. La segunda ^42^ usó la ENDES 2023 para mujeres de 15-49 años, observando diferencias puntuales, claramente variables según región y ámbito. La tercera ^43^ empleó los Demographic and Health Surveys (DHS) en un año reciente de 48 países (incluyendo ENDES 2022 de Perú, ENDI 2023 de Ecuador y EDSA 2016 de Bolivia, países con gran población de altitud), para niños de 6-59 meses, encontrando que las prevalencias diferían de manera variable según país, ámbito y, para los tres países mencionados, región subnacional, cambiando en muchos casos, pero no todos, el nivel de problema de salud pública. Estos trabajos no examinaron las tendencias temporales ni tuvieron evaluación estadística de los resultados resumidos. No obstante, sus hallazgos son consistentes con el presente estudio.

Respecto de la importancia práctica de nuestros hallazgos, notemos que el nivel del problema de salud pública, usando las categorías de la OMS ^1^, cambia de severo a moderado en muchos escenarios, cambiando, en consecuencia, la recomendación de suplementación masiva. El nivel de problema se define en base al valor de la prevalencia, y es un concepto diferente de la prevalencia de anemia severa, moderada o leve, que son grados de severidad del diagnóstico individual. Notemos también que el porcentaje de niños cuyo diagnóstico cambia (es decir, el complemento del valor predictivo total) abarca un rango amplio y variable, cambiando en consecuencia las decisiones clínicas de prevención y tratamiento ^10^. La variabilidad implica que no se puede aplicar una regla común y simple a todos los escenarios.

Hemos examinado solamente el subgrupo de 6-35 meses. Similares consideraciones pueden afectar otros subgrupos. No abordamos aquí dos problemas también críticos: la etiología ^44^, particularmente la contribución real de la ferropenia y los instrumentos ^5^. La nueva norma debe ser aplicada, cauta y gradualmente, acompañada de la lectura crítica de la evidencia disponible a medida que aparece. Como nuestra opinión planteamos las siguientes recomendaciones agrupadas en tres temas:

Sobre el tema específico del punto de corte: reconocer la norma 2024 como una corrección provisional, no completamente sustentada. En el escenario de análisis, dado que encontramos discrepancias en la técnica de ajuste para altitud, creemos mejor emplear la ecuación, no la tabla, desde el nivel del mar, y para el ajuste por edad emplear también su ecuación ^7^, aunque no lo especifique la norma, puesto que los cambios escalonados no serían consistentes con lo que se conoce de la fisiopatología. Interpretar dichos cálculos solo como una mitigación con limitaciones remanentes. Tomar en cuenta que la interpretación a medianas y grandes alturas es frágil. En el escenario de comunicación, considerar periodos con indicadores paralelos, cuidado en la transparencia de los métodos y las explicaciones.

Para el tema del escenario de control de la anemia infantil en salud pública: dada la incertidumbre no resuelta, reforzar la vigilancia para detectar subcoberturas, filtraciones, e inequidades (no solo de indicadores de hemoglobina, sino de sus determinantes, incluyendo sociales, ambientales e intervenciones), así como consecuencias de la anemia y sus intervenciones (incluyendo efectos adversos). Revisar la estrategia, reanalizando la evidencia con un margen de decisiones intuitivas, para, de ser el consenso técnico, modificar las poblaciones objetivo, el conjunto de intervenciones, y las prioridades de estudios rápidos.

Para el tema del escenario de atención preventiva y recuperativa de la salud individual: reconocer que la práctica médica debe aplicar su criterio en cada paciente, que es distinto. Actualizar los lineamientos y algoritmos de manejo individual ^10^, tomando en cuenta que los cambios son más notorios en menores de 24 meses y/o altitudes mayores a 2000 m s. n. m. Revisar los planes de gestión de servicios de salud, coberturas de aseguramiento, y procedimientos de atención normados por el estado, particularmente MINSA y EsSalud.

Notemos que la norma 2024 es conceptualmente retroactiva: implica lógicamente reconocer que estuvimos, la comunidad técnica, parcialmente equivocados en el diagnóstico y consecuente manejo, individual y poblacional, de la anemia. Esta reflexión no busca cuestionar el método científico, sino recordarnos que la ciencia no es un conjunto estático, sino que evoluciona con la crítica.

La fuente de datos de la ENDES tiene fortalezas importantes: la representatividad nacional y subnacional, su continuidad durante más de una década, y la diversidad ecológica, económica, social y cultural del Perú. Las limitaciones que podemos señalar son: (a) la reponderación, que no creemos afecte los cálculos, separados por año y sin modelamiento; (b) los cambios de diseño ^31^ en los ciclos trienales de la ENDES, como la definición de los estratos, por lo cual se redefinieron (no podemos excluir con certeza cambios asociados a las modificaciones de diseño); (c) la precisión, particularmente en los subgrupos con muestras de menor tamaño, por lo que las pequeñas diferencias no se pueden definir con seguridad; (d) la medición de sangre capilar con hemoglobinómetros portátiles HemoCue (modelo 201 en su gran mayoría); (e) la definición de una sola altitud para cada conglomerado, que puede incluir hogares en altitudes bastante diferentes; (f) el ajuste limitado de los modelos GLM en los que se basa la significancia estadística; y (g) la pequeña discrepancia con las prevalencias oficiales.

Las conclusiones del estudio son las siguientes: en niños del Perú entre 6 y 35 meses de edad, la nueva norma OMS 2024 reduce la prevalencia nacional en -6,3%, siendo la reducción variable según región administrativa, ámbito (urbano o rural) y año calendario, en un rango que va entre -40,6 a 11. El porcentaje nacional de niños cuyo diagnóstico cambia con la nueva norma es 11,0%, con nivel variable según región, ámbito y año, de 0,0 a 40,6. Los cambios observados de prevalencia y diagnóstico individual son estadísticamente significativos y de gran importancia sanitaria, en algunos casos modificando el nivel de severidad del problema. La técnica de cálculo, ecuación (que recomendamos) o tabla, afecta la prevalencia estimada en un nivel de -3,8. La literatura sustenta la dirección de la corrección, pero no completamente su magnitud.
